# Porous Organic Polymers: From Molecular Design to Scalable Technologies

**DOI:** 10.1002/smll.74396

**Published:** 2026-07-06

**Authors:** Hossein Mashhadimoslem, Hadi Shayesteh, Simindokht Zarei‐Shokat, Rokhsareh Bakhtiari, Sheyda Abedi‐Banaei, Zahra Sheikhlouiharzand, Parnia Mohebimanesh, Seyed Mohammad Amin Ojagh, Bahareh Bakhtiari, Ali Maleki, Theo G. M. van de Ven, Sohrab Zendehboudi, Ali Elkamel

**Affiliations:** ^1^ Chemical Engineering Department University of Waterloo Waterloo Ontario Canada; ^2^ Waterloo Institute for Nanotechnology University of Waterloo Waterloo Ontario Canada; ^3^ Department of Chemistry and Biomolecular Sciences University of Ottawa Ottawa Ontario Canada; ^4^ Catalysts and Organic Synthesis Research Laboratory Department of Chemistry Iran University of Science and Technology Tehran Iran; ^5^ Faculty of Engineering and Natural Sciences Sabanci University Istanbul Turkey; ^6^ Pulp and Paper Research Centre Department of Chemistry McGill University Montreal Quebec Canada; ^7^ Faculty of Engineering and Applied Science Memorial University St John's Newfoundland and Labrador Canada; ^8^ Department of Chemical Engineering Khalifa University Abu Dhabi UAE; ^9^ Research and Innovation Center on CO_2_ and Hydrogen (RICH Center) Khalifa University Abu Dhabi UAE

**Keywords:** emerging applications, morphology, organic materials, porous organic polymers, synthesis

## Abstract

For over twenty years, porous organic polymers (POPs) have emerged as promising candidates for a wide range of applications, including energy storage, gas sorption, catalysts, adsorbents, biomedical applications, sensors, and other exciting fields. The ability to fine‐tune pore function through pre‐ or post‐synthesis modification enables selective interaction with target molecules, making these materials very effective for all of the mentioned applications. This review offers a critical, up‐to‐date analysis of emerging strategies for the synthesis and post‐synthesis modification of POPs, highlighting how structural topology and chemical composition affect performance. The goal is to offer a comprehensive understanding of their scope and strategic uses. We categorize recent advances in polymeric and composite POP architectures, discuss the relationship between fabrication pathways and material functionality, and highlight the challenges that hinder their transition from laboratory discovery to scalable technologies. Additionally, we explore emerging applications, along with associated challenges and limitations, to guide future research. By connecting the fabrication processes with the performance of porous organic polymers, we can deepen our understanding of ongoing challenges and clarify future research directions.

## Graphical abstract

This review introduces porous organic polymers (POPs) for a wide range of applications, including energy storage, gas adsorption, catalysts, adsorbents, biomedical applications, sensors, and other exciting fields, and critically analyzes the synthesis and modification, and how structural topology influences the composites' architecture of POPs, along with the challenges they face during the transition from fabrication and design to scalable technologies.

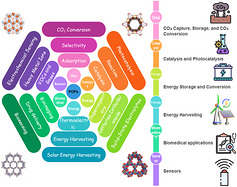

## 1 Introduction

### 1.1 Background on Porous Organic Polymers (POPs)

Porous materials have long been integral to both natural systems and engineered technologies. From the honeycomb structures used by bees to store nutrients to marine sponges filtering water through surface pores, nature offers numerous examples of efficient porous systems [1, 2]. Inspired by these biological archetypes, scientists have developed artificial porous structures tailored for various applications. Among them, porous organic polymers (POPs) have emerged as an exceptionally versatile and promising class of materials due to their tunable architectures, exceptional thermal and chemical stabilities, and high surface areas [3, 4, 5].

POPs are defined as organic solids composed entirely of light elements (e.g., C, H, N, O, B) and featuring permanent, often interconnected pores that facilitate molecular transport and surface interactions [6, 7]. According to IUPAC classification, these pores are categorized based on size: micropores (<2 nm), mesopores (2–50 nm), and macropores (> 50 nm) [8]. Furthermore, pores can be classified as either open (interconnected with external surfaces) or closed (isolated within the structure), with open porosity being particularly critical for dynamic processes such as gas adsorption and fluid transport. The design of POPs relies on monomers with inherent concavities and inefficient packing, which results in porous, interconnected structures. Beyond traditional solid‐state POPs, recent years have shown the development of porous organic polymers that are soluble in the aqueous phase. They have different structural characteristics, solution processability, and application potential. With these differing characteristics, polymers soluble in organic solvents have gained interest in the fields of biological systems, sensing, and catalysis, as well as in molecular separations. Recently published dedicated reviews have covered the extensive work in the field of aqueous phase polymers [9].

The synthetic development of POPs has significantly accelerated since the early 2000s. These materials can be amorphous or crystalline depending on their structural order and polymerization strategy. Amorphous POPs include hyper‐crosslinked polymers (HCPs), polymers of intrinsic microporosity (PIMs), conjugated microporous polymers (CMPs), and porous aromatic frameworks (PAFs), all of which lack long‐range periodicity yet exhibit significant internal surface area and tunable chemical functionalities [10, 11]. Crystalline POPs, such as covalent organic frameworks (COFs) and covalent triazine frameworks (CTFs), exhibit regular pore channels and are synthesized under thermodynamic control using modular bottom‐up strategies [12, 13, 14].

The origins of POPs can be traced back to the pioneering work of Hermann Staudinger in the 1920s, whose macromolecular hypothesis set the foundation for polymer science [4]. Over time, the design of polymers shifted toward increasingly sophisticated architectures. By incorporating multifunctional monomers and leveraging covalent organic chemistry, researchers have been able to construct POPs with controlled pore size, geometry, and chemical reactivity [14, 15, 16].

### 1.2 Importance in Modern Materials Science

The versatility of POPs positions them at the forefront of modern materials research. Their design flexibility and performance advantages over traditional porous materials such as activated carbons, zeolites, and even metal–organic frameworks (MOFs) have made them attractive for an expanding range of technological applications [17, 18]. In contrast to MOFs, where coordination bonds hold together linkers, the robust covalent bonding in POPs confers superior chemical and thermal stability, even in harsh environments such as high temperatures, humidity, or acidic conditions [19, 20]. POPs overcome several limitations associated with MOFs and COFs. Unlike MOFs, which often suffer from hydrolysis due to their coordination bonds [21], POPs are made of strong covalent bonds that have acceptable chemical and thermal stability even in humid environments [22, 23, 24]. Furthermore, POPs allow for the incorporation of different functional groups through precise monomer design, allowing for the creation of customized porous environments based on the desired application, which is challenging to achieve in crystalline minerals such as zeolites or mesoporous silica [25, 26]. In comparison to MOFs and COFs, POPs may have a lower specific surface area, which can be compensated for by their structural robustness and long‐term operational stability [1, 14]. All these features collectively make POPs a suitable and competitive option among porous materials and justify the focus of this review. Table 1 presents a qualitative comparison between POPs, MOFs and COFs in terms of material structure and performance.

**TABLE 1:** Comparison of porous organic polymers (POPs) with other porous materials.

Property	POPs [35, 36, 37, 38, 39, 40, 41, 42]	MOFs [37, 43, 44, 45, 46, 47, 48, 49]	COFs [37, 50, 51, 52, 53, 54]
Framework composition	Fully organic, covalent bonds	Metal ions + organic linkers	Fully organic, covalent bonds
Structural order	Mostly amorphous	Highly crystalline	Crystalline
Surface area	Moderate to high	Very high	High
Chemical & thermal stability	High	Moderate (often moisture‐sensitive)	Moderate to high
Presence of metal ions	None	Yes	None
Synthetic complexity	Low to moderate	Moderate to high	High
Functional tunability	Very high	High	Moderate
Pore size control	Moderate	Excellent	Excellent
Scalability	High	Limited	Limited

POPs have gained particular attention in the field of environmental remediation, where their high surface area, selective adsorption behavior, and tailored pore environments make them excellent candidates for capturing gaseous pollutants, including CO_2_ and volatile organic compounds (VOCs) [27, 28]. The ability to fine‐tune pore functionality via pre‐ or post‐synthetic modification enables selective interaction with target molecules, making POPs highly effective for gas separation and sensing applications [29]. In energy‐related domains, POPs are increasingly employed in hydrogen storage, supercapacitors (SCs), and lithium‐sulfur batteries, where their lightweight structure, ion‐accessible porosity, and redox‐active sites offer performance enhancements over conventional materials [30, 31]. Photocatalytic POPs have also emerged as sustainable alternatives for solar energy conversion, water splitting, and organic transformations. CMPs and COFs with conjugated backbones exhibit strong light‐harvesting capabilities, while triazine‐rich POPs provide nitrogen‐doped environments favorable for catalytic activity [32, 33]. The biomedical field has likewise seen growing integration of POPs, particularly in drug delivery and biosensing. The biocompatibility, modularity, and functional diversity of these frameworks enable efficient encapsulation, release, and detection of biologically relevant molecules [24, 34]. Beyond individual applications, hybrid materials combining POPs with nanoparticles or other frameworks have opened new pathways for multifunctional systems with synergistic performance [4].

### 1.3 Scope and Objectives of the Review

Research on POPs is growing rapidly, with many review articles published on different aspects of these materials. As summarized in Table 2, previous reviews have concentrated on individual topics, including gas separation, CO_2_ capture, environmental remediation, sensing, catalysis, biomedical applications, supercapacitors, and radionuclide removal. Even though these reviews have offered important perspectives on the applications of POPs, a thorough and timely analysis that systematically links synthesis strategies, structural design, functionalization approaches, emerging applications, and scalability challenges is still missing. Recent advances in fabrication technologies, post‐synthetic modifications, hybrid POP architectures, and AI‐assisted materials discovery, in particular, have not been collectively reviewed in a single review. Given the significant progress in POP research, the review aims to provide a comprehensive yet focused overview of the current state of the science of POPs. The central goal is to elucidate the relationships between synthetic strategy, framework structure, functionalization routes, and final application, thereby offering a holistic view of the field and guidance for future exploration. We begin by categorizing POPs by crystallinity and molecular design, distinguishing between amorphous systems, such as HCPs, CMPs, and PIMs, and crystalline frameworks, including COFs and CTFs. In the fabrication section, attention is given to traditional polymerization techniques as well as emerging synthetic approaches, mechanochemistry, and microwave irradiation. Functionalization strategies are discussed in detail, with an emphasis on how chemical tailoring, particularly the incorporation of amines, modulates the adsorption capacity and selectivity of POPs toward CO_2_ and other gases. Pre‐synthetic and post‐synthetic modification methodologies are compared, and emerging functional groups are evaluated for their potential in advanced applications. The applications section surveys the performance of POPs in gas adsorption and separation, catalysis, energy storage, environmental treatment, and biomedicine. Special attention is given to hybrid and composite POPs and their integration into a nanotechnology‐driven solution. Finally, the review addresses the current challenges hindering broader adoption of POPs, such as difficulties in achieving precise morphology control, limited processability, and questions surrounding long‐term environmental safety and scalability. We outline future directions that emphasize green synthesis, modular frameworks, and industrial integration strategies.

**TABLE 2:** Recent review on porous organic polymers.

Year	Topic	Refs.
2026	Porous organic polymers based on carbon–carbon coupling reaction	[55]
2026	Environmental applications of porous organic polymers	[56]
2025	Synthesis and applications of hyper‐crosslinked porous organic polymers	[57]
2024	Porous organic polymers for radionuclide metal ions separations	[7]
2024	Porous organic polymers for sustainable gas separations	[18]
2024	Porous organic polymers for energy, environmental, and biomedical applications	[58]
2023	Porous organic polymers for environmental remediation	[59]
2022	Porous organic polymers for Li‐chemistry‐based batteries	[35]
2022	Water‐soluble and dispersible porous organic polymers	[9]
2022	Porous organic polymers for sensing applications	[60]
2022	Porous organic polymers for CO_2_ capture	[61]
2022	Porous organic polymers for biomedical applications	[36]
2022	Advances in porous organic polymers: syntheses, structures, and diverse applications	[14]
2022	Porous organic polymers for high‐performance supercapacitors	[62]
2022	Porous organic polymers for CO_2_ capture, separation, and conversion	[63]
2022	Amine‐functionalized porous organic polymers for CO_2_ capture	[28]
2019	Porous organic polymer for efficient water capture	[64]
2012	Functional porous organic polymers for heterogeneous catalysis	[65]

## 2 Classification of POPs

**FIGURE 1:**
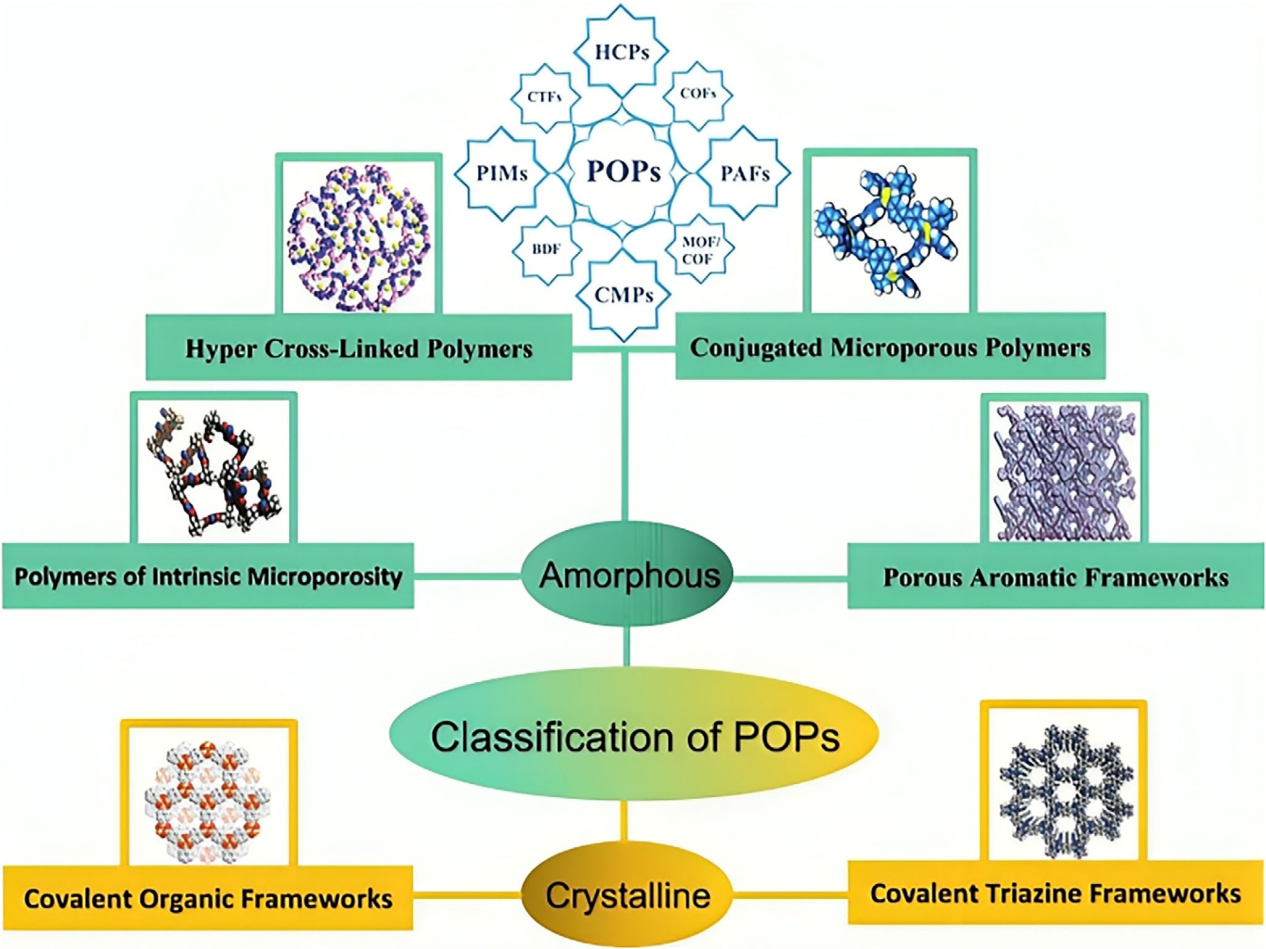
Schematic classification of PIMs, PAFs, HCPs, CMPs, COFs, and CTFs based on their morphology.

In an endeavor to systematically study each family of advanced materials, categorizing them based on their morphological characteristics into distinct subgroups helps to discern the connection between molecular architecture and macroscopic properties. Diligent partitioning of POPs not only elucidates the structural diversity inherent in POPs but also provides a framework for predicting and manipulating their porosity, surface area, and mechanical stability [59, 66]. Through this classification, POPs are divided into two groups: amorphous POPs and crystalline POPs (Figure 1). The macroscopic morphology of porous materials has a profound influence on their adsorption capabilities with respect to organic solvents, resulting from the interplay between the material's structural architecture and the physicochemical properties of the adsorbates, including molecular size, polarity, and interaction strength with the adsorbent surface [14].

### 2.1 Amorphous POPs

Amorphous morphology is observed in materials that possess a short‐range periodicity inside their structure, which engenders substantial heterogeneity in pore size and shape, plus the non‐crystalline configuration. The majority of POPs fall into this subset, which arises from the stochastic nature of polymerization processes, the primary factor for preventing the formation of a periodic lattice [67]. This inherent disorder can be advantageous in scenarios where a rigid, uniform pore structure is not required, as it allows for greater adaptability in accommodating guest molecules of varying sizes and shapes. Moreover, the amorphous nature often imparts enhanced mechanical flexibility and resilience, making these materials suitable for dynamic environments where structural integrity under stress is critical. Within this group, polymers of intrinsic microporosity (PIMs), CMPs, hyper crosslinked polymers (HCPs), and PAFs are included, while COFs are excluded since they have a crystalline form. CTFs play a role in between, since they can crystallize under thermodynamically‐controlled conditions and show an amorphous form, by following different parameters of synthesis. Over this part, a deep explanation of each kind, along with the recent works, has been presented in detail [66, 67].

#### 2.1.1 PIMs

**FIGURE 2:**
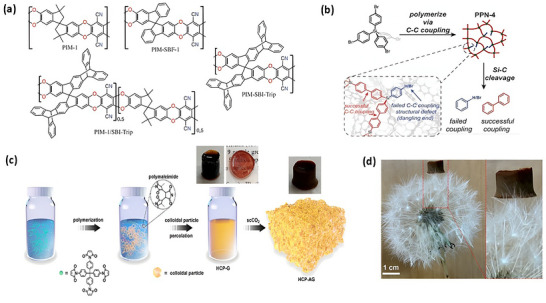
(a) Chemical structures of PIM‐1, PIM‐SBF‐1, and PIM‐SBI‐Trip, along with their copolymer PIM‐1/SBI‐Trip. Reproduced with permission from ref. [73]. Copyright 2024, Wiley‐VCH. (b) network disassembly analysis strategy for the quantification of defects in tetra‐aryl silane PAFs. Dangling end defects will manifest as phenyl fragments once they undergo exhaustive desilylation, while biphenyl fragments are indicative of successful monomer coupling. Reproduced with permission from ref. [76]. Copyright 2020, Wiley‐VCH. (c) synthesis of HCP gels and aerogels via gelation processes, comprising (i) dispersion solution of the monomer in N, N‐dimethylformamide (DMF), (ii) formation of the colloidal particles, (iii) aggregation and percolation of the colloidal particles to form the HCP gel, and (iv) solvent exchange with acetone and scCO_2_ drying to yield the HCP aerogel. The insets are photographs of the HCP gel and aerogel. (d) photograph of a chunk of 35‐mg HCP standing on top of dandelion hairs. Reproduced with permission from ref. [81]. Copyright 2017, American Chemical Society.

This type of polymer was first disclosed by McKeown and Budd in 2004, which focused on the synthesis of PIM‐1, a specific PIM material, through a reaction between a tetraol (5,5′,6,6′‐tetrahydroxy‐3,3,3′,3′‐tetramethylspirobisindane) and tetrafluoroterephthalonitrile [68]. Their defining feature, permanent microporosity, is a direct outcome of the rigid and contorted nature of their polymer chains, preventing them from collapsing into a densely packed structure [69]. By limiting bond rotation and conformational freedom, the polymer chains cannot rearrange themselves to eliminate voids or pores, and this restriction is manageable through entering rigid and often fused ring systems that connect the structural units during polymerization. Linking groups such as dibenzodioxin, imide, or Tröger's base are designed to prevent rotational freedom between adjacent structural units, thereby maintaining the microporous architecture [70, 71, 72]. Reducing the extent of cohesive interactions between chains helps solvents to penetrate into the polymer matrix, resulting in the good solubility of PIMs in common organic solvents such as tetrahydrofuran (THF) or chloroform. This good solubility brings advantages such as enabling their solution processing into various forms (e.g., self‐standing films, hollow fiber membranes, thin films) and using solution‐based techniques for characterization (e.g., nuclear magnetic resonance (NMR) spectroscopy and gel permeation chromatography (GPC))[69, 73]. A deliberate strategy, created by C. Grazia Bezzu et al., to produce PIM‐SBI‐Trip and PIM‐1/SBI‐Trip as ultrapermeable and highly rigid polymers with enhanced gas separation performance, is the use of spirobisindane (SBI) and triptycene (Trip) units in the monomer design to combine the synergistic effects of both units (Figure 2a). The contorted spirobisindane unit and the bulky triptycene unit work together to create a highly porous structure. The spiro‐center introduces contortions in the polymer backbone and prevents dense chain packing, while the triptycene units with a paddle‐wheel‐like structure increase the interchain distance, resulting in a large free volume and microporosity. The polymers PIM‐SBI‐Trip and PIM‐1/SBI‐Trip were synthesized via the dioxin‐forming aromatic nucleophilic substitution (SNAr) reaction between SBI‐Trip (6) and 2,3,5,6‐tetrafluoroterephthalonitrile (TFTPN). PIM‐SBI‐Trip and PIM‐1/SBI‐Trip exhibited high Brunauer–Emmett–Teller (BET) surface areas of 930 m^2^/g and 858 m^2^/g, respectively, which are higher than those reported for PIM‐1 and PIM‐SBF‐1, confirming the enhanced microporosity. Moreover, both of the PIMs demonstrated ultrapermeability and high selectivity for various gas pairs (e.g., CO_2_/N_2_, CO_2_/CH_4_), making them suitable for carbon capture and biogas upgrading [73].

#### 2.1.2 PAFs

PAFs are constructed from aromatic building units linked through irreversible carbon‐carbon covalent bonds, a hallmark of their structural integrity and resistance to harsh chemical environments. Despite their amorphous nature, as evidenced by powder X‐ray diffraction (PXRD), PAF‐1 was produced using tetrakis (4‐bromophenyl) methane as a starting monomer via a Yamamoto‐type Ullmann coupling reaction and revealed a record‐breaking BET surface area of 5600 m^2^/g [74]. This monumental achievement, as reported by the authors, demonstrates that amorphous materials can rival, and even surpass, the porosity of their crystalline counterparts. The term “aromatic” in PAFs refers to the abundant benzene rings within their structure, which give rigidity and exceptional chemical resistance to acidic, alkaline, and humid environments, making them suitable for industrial applications [75, 76]. Moreover, the abundance of aromatic compounds enables sufficient reactivity for post‐synthetic modifications (PSMs) through the incorporation of specific functional groups. The failed or incomplete coupling reactions during polymerization result in defects that contribute to the amorphous nature of most PAFs. These defects can be manifest in several ways, such as when monomers fail to couple and leave behind gaps in the polymer network, or when they partially react, and some functional groups remain unreacted. These defects disrupt the regularity of the polymer network, affecting its porosity, surface area, and pore architecture. For instance, they lead to collapsed pores, non‐porous regions, irregular pores, or pore blockages. However, one of their potential benefits is the addition to the number of adsorption sites, which enhances gas sorption capacity in some cases. Anthony J. Porath and his colleagues explored the role of defects in regulating PAF properties and introduced a network disassembly approach to quantify defects and their impact on porosity. A schematic illustration of this approach is shown in Figure 2b [77]. PPN‐4, a prototypical high‐surface‐area PAF, was synthesized via three different types of coupling reactions from tetra‐aryl silane monomers and degraded into smaller fragments to study the connection between synthetic reactions and structural defects and microporosity. Using this approach, PPN‐4 was fabricated through Yamamoto‐Type Ullmann (YTU) polymerization, Negishi coupling, and Murahashi‐like homocoupling, which was subsequently broken down via chemical methods (e.g., hydrolysis of specific bonds).

#### 2.1.3 HCPs

Hypercrosslinked systems reflect the continuous quest for materials with enhanced porosity, stability, and functionality that the previous ones lacked. Among all POPs, the development of this class dates back to the 20th century, when cross‐linked polystyrene networks, based on divinyl benzene (DVB), were first reported in 1934 by Staudinger and Heuer [78], densely crosslinked 3D framework results in an interconnected network of pores throughout the whole structure, which resists collapse even after solvent removal and other harsh conditions. Besides, the robust covalent bonds within HCPs endow excellent chemical and thermal stability, by allowing them to withstand acidic, basic, and high‐temperature environments, which makes them super advantageous for industrial applications [79, 80, 81]. Traditional HCPs are typically synthesized as insoluble powders, are difficult to shape into macroscopic objects (e.g., monoliths, films, or membranes), and combine with other materials (e.g., COFs) to create hybrid systems [82]. The self‐polymerization of 1,1′,1″,1″′‐(methanetetrayltetrakis(benzene‐4,1‐diyl)) tetrakis (1H‐pyrrole‐2,5‐dione) (TPM‐4MI) as starting monomer (Figure 2c), was initiated by its aggregation into colloidal particles with sizes of ∼200–500 nm, which upon thermal induction (90°C) were solubilized and polymerized from the side of maleimide groups without the need for catalysts or any initiators. After continuously heating the mixture for a day, solvent exchange with acetone and supercritical CO_2_ (scCO_2_) drying converted the obtained transparent brown gel, in which solvent molecules were trapped inside its network, into an aerogel (HCP‐AG) while retaining its 3D network. The porous and interconnected network resulted in a low‐density material, making the gel as light that it could stand on top of dandelion hairs, as shown in Figure 2d.

#### 2.1.4 CMPs

CMPs are given prominence by scientists since they were first introduced in 2007 by Cooper as a specific group of POPs [83], characterized by the extended π‐conjugated systems of double and triple bonds that are integral to their polymeric backbone. As a result, they exhibit inherent microporosity and electronic delocalization, which imparts unique electronic and optical properties to CMPs [84, 85, 86]. To produce the skeleton of CMPs in 3D form, the starting monomers must possess at least two reactive groups to form covalent bonds either by crosslinking or homocoupling, which again depends on the monomers. The tunable band gap and semiconducting property can be adjusted by modifying the monomeric units or the degree of conjugation, making them particularly suitable for applications in organic electronics and photocatalysis [25]. Although PAFs and CMPs share many similarities, the lack of continuous π‐conjugated systems across their polymeric backbones results in insulating or weakly conducting behavior in them. Applying tetrahedral monomers disrupts the continuous π‐conjugation system due to the sp^3^ hybridization of the central atom in tetrahedral structures, which prevents the delocalization of π‐electrons across the framework [87]. The cross‐linked structure of CMPs endows them with a highly rigid skeleton, which makes their processing into practical forms, such as membranes, somewhat challenging. A novel subclass of CMPs, titled as conjugated microporous thermoset (CMT), was developed by Liu et al., has the capability of solution processability and a narrow pore size distribution of 0.4–0.5 nm, and was adopted for the separation of H_2_ gas from mixtures [88]. The starting monomer of the CMT (3,6,12,15‐Tetrabromotetrabenzo[a,c,h,j]phenazine (3‐TBTBP)) is tetrabrominated planar molecule which is insoluble in water but undergoes sublimation (at ∼450°C), melting (at ∼509°C, forming a molecular liquid), and endogenous liquid‐state polymerization (at ∼520°C) as confirmed by Thermal Gravimetric Analysis (TGA) and Differential Scanning Calorimetry (DSC). The CMT can be processed into 3D, 2D, and 1D forms, easing the fabrication of crack‐free films, nanotubes, and large‐area membranes with incredible tolerance to high temperatures (150°C) for 700 h.

### 2.2 Crystalline POPs

Crystalline POPs refer to the periodic arrangement of molecules or atoms in an ordered manner that extends through the whole structure. Such structural uniformity confers consistent pore dimensions and geometries, permitting a refined exploration of structure‐property correlations. Linking molecular building units with strong, irreversible covalent bonds is thermodynamically favored; thus, amorphous structures, such as cross‐linked polymers, are easier to produce. By meticulously controlling the thermodynamic and kinetic factors that govern crystallization, correcting defects through break and reform will be possible in order to obtain crystalline structures [89, 90, 91].

#### 2.2.1 COFs

**FIGURE 3:**
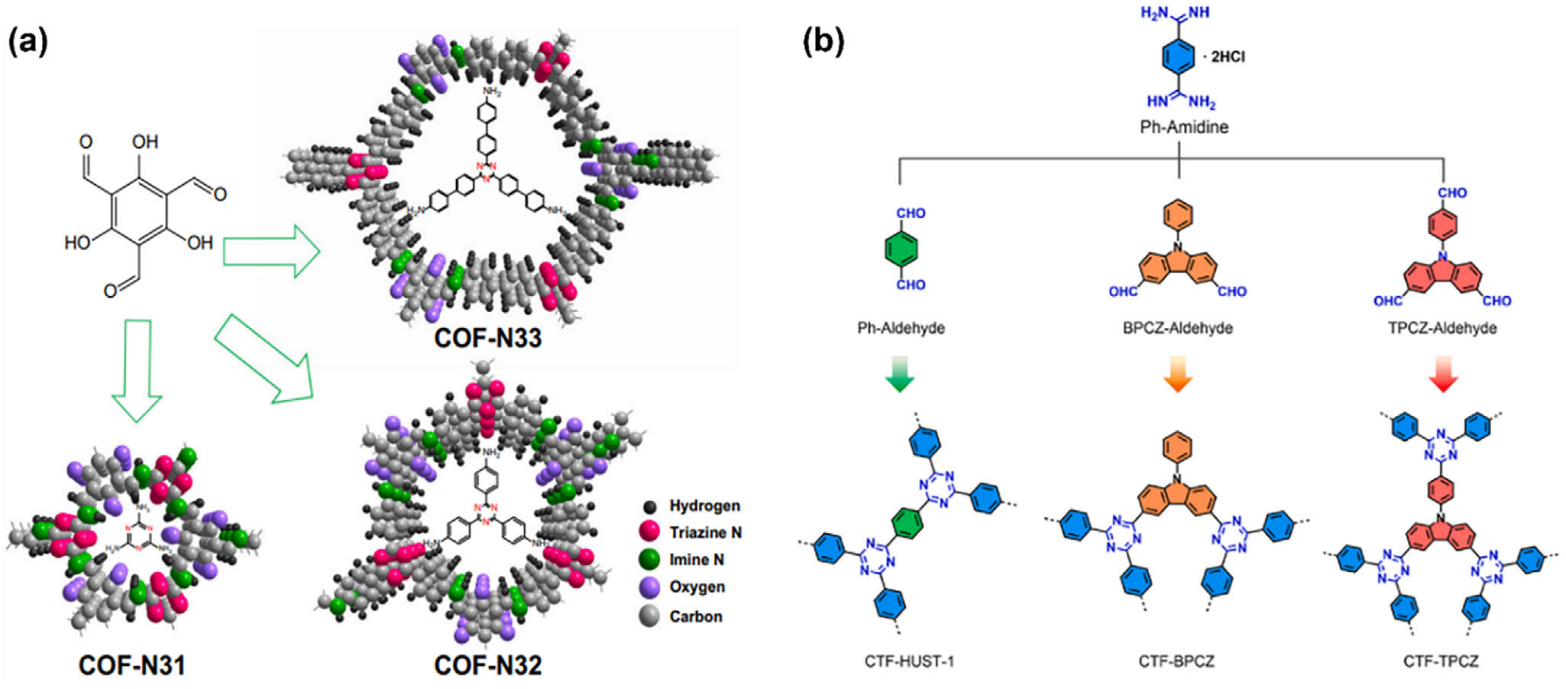
(a) Schematic illustration of the synthesis process of COF‐N31, COF‐N32, and COF‐N33. Reproduced with permission from ref. [100]. Copyright 2023, Nature. (b) schematic representation of the synthesis of CTF‐HUST‐1, CTF‐BPCZ, and CTF‐TPCZ. Reproduced with permission from ref. [104]. Copyright 2024, Elsevier.

COFs, pioneered by Yaghi and colleagues in 2005 [12], are indeed the most outstanding class of organic materials composed of light, non‐metallic elements (e.g., carbon, hydrogen, nitrogen, oxygen, and boron) with a reticular porous structure. The pore size (typically ranging from micropores (<2 nm) to mesopores (2–50 nm)) and total surface area (up to several thousand m^2^/g) can be tailored by choosing appropriate building blocks and linkers [92, 93]. A distinguishable property of COFs is their dimensional architectures, which are central to their structural and functional characteristics. Two‐dimensional (2D) COFs like COF‐1 and COF‐5 [93]. are defined by their stratified, sheet‐like structures, where individual layers are cohesively bounded by weaker interactions of *π*–*π* stacking and van der Waals forces to yield in‐plane porosity [94, 95]. In contrast, three‐dimensional (3D) COFs such as COF‐300 [96] and COF‐102 [97], extend their covalent connectivity across all spatial dimensions, culminating in intricate, interwoven networks of interconnected pores. These frameworks exhibit more elaborate architecture and generally possess superior surface areas compared to the former ones. Donor‐acceptor COFs are a specialized class containing electron‐rich moieties (e.g., phenyl groups, enamine groups) that donate electrons when excited by an external stimulus, and electron‐deficient moieties (e.g., triazine rings, carbonyl groups) that accept electrons [98, 99]. This asymmetric distribution of electrons between the donor and acceptor units creates intramolecular polarity, which drives charge separation and exciton dissociation. A set of D‐A COFs (COF‐N31, COF‐N32, and COF‐N33) was designed by incorporating varying numbers of phenyl groups (n = 0, 1, 2) as electron donors and triazine rings as electron acceptor groups for efficient exciton regulation and photocatalytic H_2_O_2_ production (Figure 3a) [100]. Analyzing the data reveals the inverse relationship between polarity and the number of phenyl groups, indicating that spatial separation of charges reduces electron‐hole recombination in the process, which is supported by the results: COF‐N31 (0.072 e Å^−1^) > COF‐N32 (0.032 e Å^−1^) > COF‐N33 (0.020 e Å^−1^). The weak polarity of COF‐N31 restricts exciton dissociation due to strong π‐conjugation, but inhibits exciton formation. In contrast, the strong polarity in COF‐N32 acts oppositely and reduces the efficiency. Observations indicated the stable H_2_O_2_ yield and excellent reusability of COF‐N33 and COF‐N32 after several cycles because of direct oxidation of water to H_2_O_2_ by h^+^, which prevents self‐attack. However, a dramatic performance decline of COF‐N31 over cycles was observed as h^+^ attacks the COF itself, leading to self‐decomposition and low stability.

#### 2.2.2 CTFs

Covalent triazane frameworks (CTFs), introduced by Thomas and colleagues in 2008 [101], are a seminal series of polymers containing aromatic C═N linkages (triazine units) in their structure. The robust framework chemistry confers exceptional chemical and thermal stability, ensuring resistance to aging, thereby offering extended service life for a wide array of applications, including photocatalysis and heterogeneous catalysis [102]. Additionally, the elevated nitrogen content enhances the material's affinity for polar molecules and gases, supporting their use in gas separation and storage. CTFs occupy a unique intermediate position in the spectrum of POPs because of their dual nature, which sets them apart from both COFs and CMPs in terms of their crystalline and amorphous characteristics. As discussed previously, the crystalline structure of COFs is attributed to their reversible covalent bonds, which come at the cost of chemical stability, as weaker bonds make them susceptible to hydrolysis and degradation. On the other hand, the amorphous morphology of CMPs stems from the irreversible covalent bonds in their skeletons. CTFs bridge this gap by offering a semicrystalline or amorphous nature due to their ultra‐strong triazine linkages, which hinder the formation of ordered structures. The synthesis of CTFs often involves trimerization reactions of nitrile‐containing monomers, resulting in a highly cross‐linked network with uniform porosity and high surface area [103]. COFs are not the exclusive D‐A structures for photocatalytic H_2_O_2_ production; CTFs functionalized by phenyl carbazole (electron donor) and triazine (electron acceptor) groups reached a higher grade of solar‐to‐chemical efficiency (SCC). In a project led by Ruixue Sun and her colleagues, D‐A type CTFs, namely CTF‐BPCZ (containing two phenyl carbazole units per triazine ring) and CTF‐TPCZ (containing three phenyl carbazole units per triazine ring), are illustrated in Figure 3b, were synthesized via polycondensation of bimolecular benzamidine hydrochloride with monomolecular double or triple aldehyde‐substituted phenyl carbazole [104]. DFT calculations revealed the negative impact of spatial separation between HOMO, localized on electron‐donor groups, and LUMO orbitals, localized on triazine rings, on the electron‐hole recombination rate, resulting in lower energy barriers and improved photocatalytic efficiency. Compared to the COF‐N32, which was mentioned in the previous part, CTF‐TPCZ achieves an SCC of 0.68%, which is more than double the SCC of COF‐N32 (0.31%), highlighting the superior efficiency of CTF‐TPCZ in converting solar energy into chemical energy (H_2_O_2_).

## 3 Fabrication Strategies of POPs

### 3.1 Synthesis Techniques

The selection of an optimal synthesis technique for POPs depends on the characteristics of the target material and application. Accordingly, the relative merits of each method should be evaluated by considering key factors such as structural control, physicochemical properties, scalability, cost‐effectiveness, and process efficiency. In cases where high crystallinity and well‐defined pore characteristics are required, the sol‐hydrothermal method is an excellent choice, and acceptable stability can be achieved [105, 106]. It is also more suitable when particle size and morphology are specifically required [107], for instance, in biomedical applications, nanoscale particles have a better chance of entering cells and are better distributed in the bloodstream [36]. However, the practicality of this technique is limited because it is inherently energy‐intensive due to prolonged high temperatures, difficult to scale up, and sometimes hazardous due to the use of toxic solvents. On the other hand, the green competitor mechanochemistry is the solvothermal method because it is solvent‐free [108]. This technique results in the formation of amorphous or less crystalline materials, and there is always the possibility of contamination by pellets or the inner layer of the glass [109]. Interfacial polymerization (IP) is another technique for preparing films and coatings at the interface of two immiscible phases under ambient conditions, but some crosslinking (e.g., thermal polymerizations) cannot be carried out at the interface and is therefore only applicable to a limited range of POPs. Fast interfacial reactions help in the formation of thin films and can trap defects, poor lattice order, and lower crystallinity [110]. Microwave‐assisted synthesis has many advantages, such as good crystallinity, reduced reaction time, uniform heating, and, since it usually does not require high pressure or temperature, it is safer to use [111]. These techniques are discussed in detail in separate sections, and recent work using each is presented in detail. Collectively, these sections elucidate the relationships between POP structures, synthesis methods, and functionalization strategies, and clarify how multiple parameters collectively influence the rational selection of POPs for targeted applications.

#### 3.1.1 Condensation Reactions

Condensation reactions engender covalent linkages between two molecules through the loss of smaller molecular entities, such as water or alcohol, in the process. Condensation reactions follow different paths, even though they are essentially the same. The top condensation reactions used in the synthesis of POPs are as follows:

##### 3.1.1.1 Schiff Base Condensation

**FIGURE 4:**
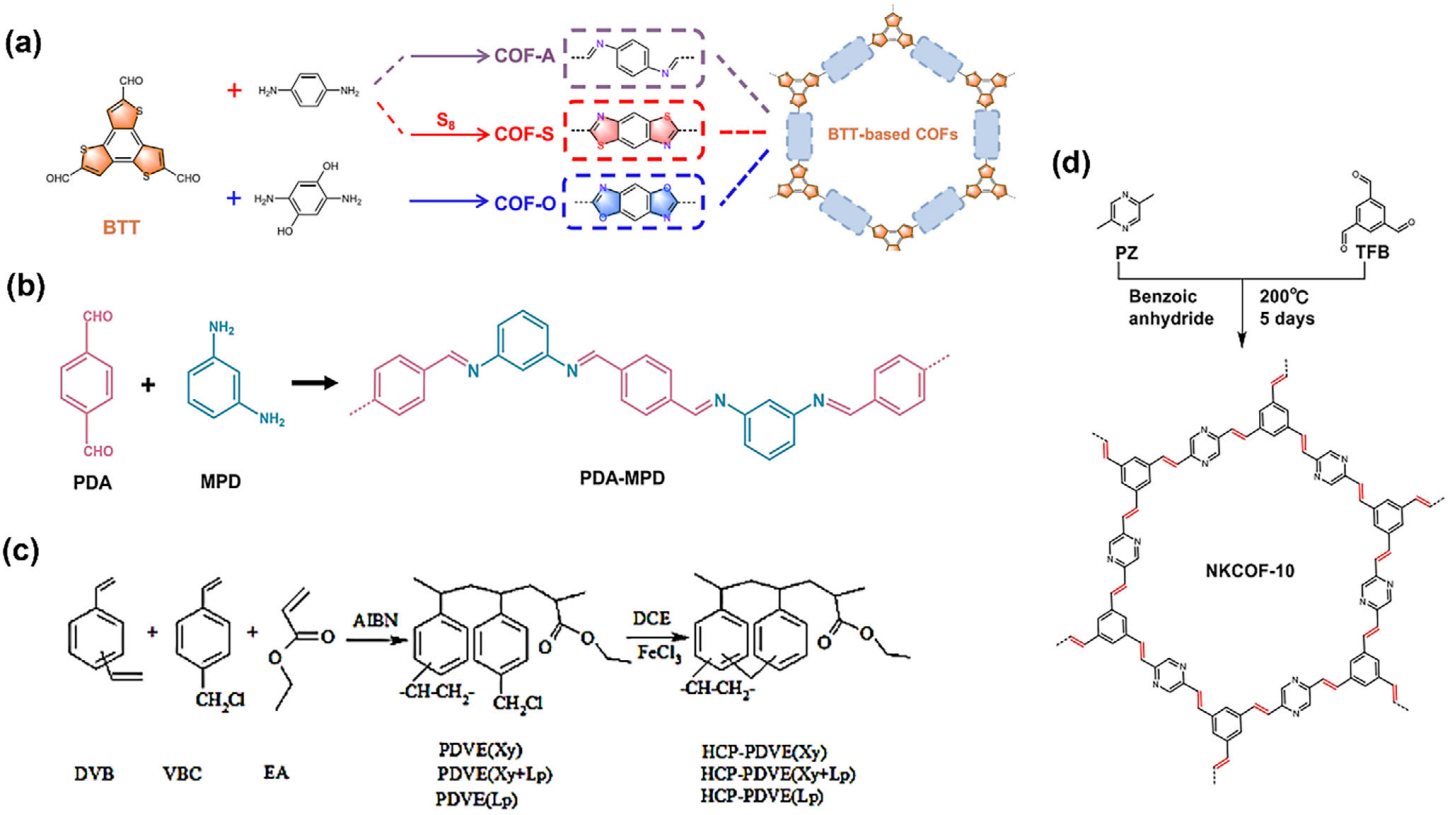
(a) Preparation scheme of COF‐A, COF‐O, and COF‐S via Schiff‐base condensation reaction. Reproduced with permission from ref. [113]. Copyright 2024, Nature. (b) synthesis of CMP through the condensation polymerization of the aldehyde monomer of PDA and the amine monomer of MPD through a Schiff‐base condensation reaction. Reproduced with permission from ref. [114]. Copyright 2024, Elsevier. (c) synthetic procedure of ethyl acrylate‐functionalized hyper‐cross‐linked polymers through the Friedel‐Crafts reaction. Reproduced with permission from ref. [116]. Elsevier. (d) a Structure and synthesis of NKCOF‐10. Reproduced with permission from ref. [118]. Copyright 2021, Nature.

Through this reaction, a primary amine (as a nucleophile) attacks a carbonyl group (aldehyde or ketone) to form an imine linkage (C═N), followed by the elimination of a water molecule at the end. This reaction is beneficial since it enables us to incorporate different functional groups into the polymer backbone and serves as an active site for further modification or functionalization [112]. The reversibility of imine bonds under elevated temperatures or acidic/aqueous conditions allows error correction during crystallization, but this hydrolysis in photocatalytic applications can degrade imine‐linked COFs back to the initial precursors. To resist hydrolysis, Hou et al. replaced the imine bonds with rigid aromatic heterocycles (oxazole/thiazole) via a facile, one‐pot process that eliminates the need for PSMS, and obtained COFs with higher photocatalytic activity [113]. In this experiment, a Schiff base condensation between 1,3,5‐benzenetricarbaldehyde (BTT) and benzene‐1,4‐diamine in a mixture of mesitylene/dioxane with acetic acid catalyst resulted in the COF‐A. Using the same precursors (BTT + benzene‐1,4‐diamine) but applying different conditions (elemental sulfur (S_8_) in DMSO/o‐dichlorobenzene), this time, the imine linkage converted into a thiazole ring (C_3_NS) via sulfur incorporation. The key benefits of this cyclization are the enhanced π‐conjugation, which leads to improved charge separation and a lower O_2_ activation barrier. By substituting the initial reagents from benzene‐1,4‐diamine to 2,5‐Diaminobenzene‐1,4‐diol and reacting them in NMP/mesitylene at 185°C, the hydroxyl groups facilitate oxazole ring (C_3_NO) formation (COF‐O) (Figure 4a). Schiff‐base condensation reactions are not confined to solvent‐mediated systems, remarkably, they can be operatable efficiently in the gas phase without requiring catalytic assistance, while maintaining excellent reaction yields and selectivity, as Kai Mi and others used it in a molecular layer deposition (MLD) method to synthesize a CMP membrane from terephthalaldehyde (PDA) and m‐phenylenediamine (MPD) precursors (Figure 4b) [114]. Through this polycondensation, the aldehyde (–CHO) groups of PDA react with the amine (–NH_2_) groups of MPD, forming imine (C═N) linkages with the elimination of water. The mild 100°C deposition temperature critically ensures the compatibility of the CMP membranes on various substrates by preserving the structural integrity of temperature‐sensitive polymer substrates, and allows their deposition on diverse substrates, including plastics and pre‐fabricated membrane modules.

##### 3.1.1.2 Friedel‐Crafts Alkylation/Acylation

In this type of reaction, alkyl or acyl groups in the alkyl or acyl halides are added to the aromatic compounds in the presence of a Lewis acid catalyst (e.g., AlCl_3_), where an HCl molecule is released [115]. Some of the POPs, such as CMPs, HCPs, and PAFs, are synthesized through this reaction. Aiping Hao and his co‐workers fabricated an HCP polymer via a two‐step process: (i) suspension polymerization of ethyl acrylate (EA), divinylbenzene (DVB), and vinylbenzyl chloride (VBC) to form an initial copolymer (PDVE), followed by (ii) FeCl_3_‐catalyzed Friedel‐Crafts alkylation to create a rigid, porous network, as depicted in the Figure 4c [116]. In the initial step, o‐xylene and paraffin were employed as porogens, where the former (o‐xylene) dissolved both monomers and polymer while simultaneously delaying phase separation, and the latter induced early polymer precipitation. In the radical addition of EA, DVB, and VBC to form PVDE, VBC introduces –CH_2_Cl pendants for later Friedel‐Crafts reactions, while DVB cross‐links the chains. For Friedel‐Crafts cross‐linking, 1,2‐dichloroethane (DCE) as solvent and FeCl_3_ as catalyst were employed.

##### 3.1.1.3 Aldol Condensation

Aldol condensation is referred to as a two‐step reaction between two carbonyl groups, either in ketone or aldehyde form, which reversibly produces a β‐hydroxyaldehyde or ketone (the aldol product) in the first step and subsequently undergoes irreversible dehydration to create an α, β‐unsaturated carbonyl compound [117]. Zhifang Wang and co‐researchers assembled a proton‐conductive olefin‐linked COF termed NKCOF‐10 via a solvent‐free, benzoic‐anhydride‐catalyzed Aldol reaction [118]. The Aldol Condensation formed a β‐hydroxy ketone intermediate out of 2,5‐dimethylpyrazine (PZ) and 1,3,5‐triformylbenzene (TFB) precursors under benzoic anhydride catalysis, which subsequently dehydrates to yield an α, β‐unsaturated ketone (olefin linkage), as illustrated in Figure 4d. The intrinsic proton conductivity of this COF is rooted in the adsorption of water molecules onto pyrazine nitrogen sites via hydrogen bonding (O–H···N), resulting in a value of 1.08 × 10^−5^ S cm^−1^ at 80°C (90% RH) in anhydrous conditions.

##### 3.1.1.4 Knoevenagel Condensation

The Knoevenagel condensation involves the nucleophilic addition of a β‐hydrogen‐containing compound (e.g., malononitrile, ethyl cyanoacetate) to an aldehyde or ketone, where an α, β‐unsaturated carbonyl compound is yielded. A base catalyst, usually a weak amine, is needed in the reaction. This reaction is applicable for the synthesis of various organic compounds, including those with conjugated systems [119]. Ya‐Jie Li1's team synthesized two sets of olefin‐linked COFs with exceptional electrochemiluminescence (ECL) performance, which surpass conventional phosphors in aqueous media. These COFs were constructed via Knoevenagel and aldol condensations using tailored monomers. Piperidine‐mediated deprotonation of the methyl groups in 2,4,6‐trimethylpyridine‐3,5‐dicarbonitrile (DCTP) in Knoevenagel condensation generates a stabilized enolate intermediate. This nucleophilic species undergoes addition to the carbonyl carbon of various aromatic aldehydes (e.g., BDA, TDA, EDA, DAFB, BTTA, TFPB, TPA, TFPT), and forms a β‐hydroxy intermediate. Subsequent dehydration yields an α, β‐unsaturated nitrile (–C = C–CN), establishing a rigid, conjugated backbone. In the Aldol condensation, trifluoroacetic acid (TFA) activates the methyl groups in the 2,4,6‐trimethyl‐1,3,5‐triazine (TMT) structure, producing a β‐hydroxy ketone intermediate. Heat‐driven dehydration then forms C═C bonds, further enhancing conjugation. Strongest ECL signals were observed in DAFB‐DCTP and TFPT‐TMT, attributed to optimal HOMO‐LUMO separation and extended π‐conjugation from rigid olefin linkages, which minimizes non‐radiative decay [120].

#### 3.1.2 Cyclotrimerization Reactions

**FIGURE 5:**
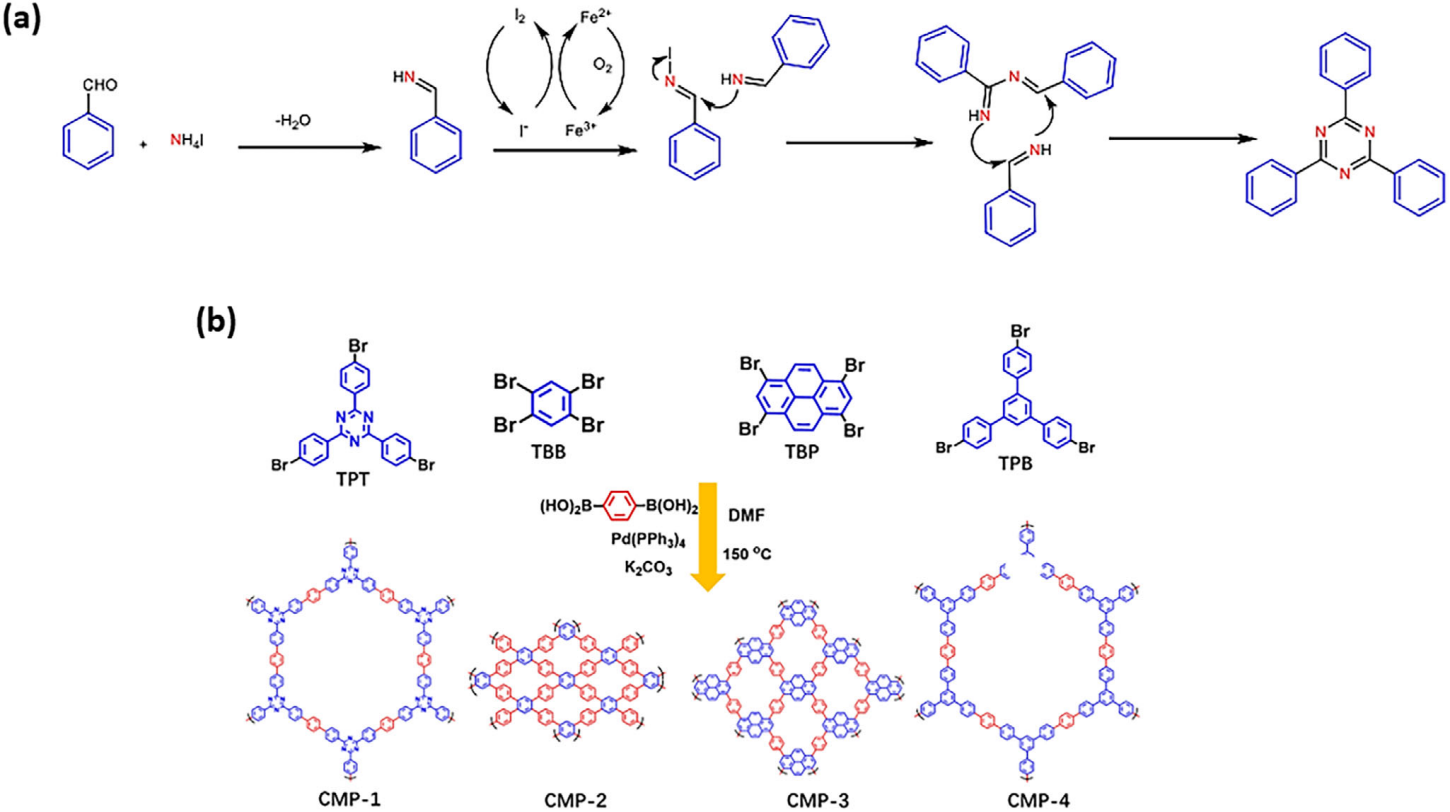
(a) Proposed reaction mechanism for the synthesis of the triazine unit through cyclotrimerization reaction. Reproduced with permission from ref. [122]. Copyright 2023, Nature. (b) synthesis route and molecular structure of CMP precursors via Suzuki coupling reaction. Reproduced with permission from ref. [124]. Copyright 2020, American Chemical Society.

Cyclotrimerization is explained as a chemical ring‐forming reaction where three monomers combine to form a cyclic structure (a ring of three units). The reaction can be performed in different conditions (e.g., solution‐based, molten, solvent‐free) to control the shape and properties of the POPs and is mostly applied for the synthesis of triazine‐based POPs from triazin units [121]. A study by Guan et al. presents a new, mild, and open‐air cyclotrimerization of fluorinated aromatic aldehydes using NH_4_I, which serves as both a nitrogen source and a catalyst precursor, with control experiments showing its necessity for product formation to synthesize high‐fluorine CTFs (Figure 5a) [122]. This novel method uniquely preserves C–F bonds (30.2 wt.% F in CTF‐TF), as verified by C–F IR stretches (983 cm^−1^) and elemental analysis, and also overcomes the C–F cleavage limitations of high‐temperature approaches. The resulting perfluorinated CTF‐TF, when doped with H_3_PO_4_, achieves record proton conductivity (1.82 × 10^−1^ S cm^−1^ at 150°C) through dual hydrogen‐bonding networks between H_3_PO_4_ and both triazine N/F sites.

#### 3.1.3 Coupling Reactions

A set of popular reactions in polymer chemistry is coupling reactions, which yield strong covalent bonds. Coupling reactions often require transition‐metal catalysts, while condensation reactions might use acid or base catalysts. Also, coupling reactions are generally irreversible, whereas condensation reactions can be reversible under certain conditions. As this group differs from the above‐mentioned ones and contains various forms, a separate exploration seems inevitable.

##### 3.1.3.1 Suzuki–Miyaura Coupling

Suzuki coupling is a cross‐coupling reaction between an aryl or vinyl boronic acid (or boronate ester) and a halide, resulting in the formation of a carbon‐carbon (C–C) bond in the presence of palladium as the catalyst. A key advantage of this reaction is its mild reaction conditions, which are normally at low temperatures. Additionally, it is considered to be less harmful to the environment compared to other coupling reactions [123]. This reaction was used to link bromo‐monomers to 1,4‐phenylenebisboronic acid (Figure 5b) to produce CMP precursors for electromagnetic interference (EMI) shielding use. Because of the low electrical conductivity (EC) of CMPs, they don't exhibit good EMI shielding effectiveness (SE); however, this challenge has been surpassed by pyrolyzing CMPs under inert conditions to create conductive carbon materials. After the coupling reaction, anaerobic carbonization under an argon atmosphere at 900°C, changed precursors to porous carbon nanomaterials (PCNs), which, according to thermogravimetric (TG) analysis, happens in the range of 600–800°C due to the big weight loss. The EC measurements of four CMPs showed PCN‐2 and PCN‐4 (derived from polyphenyl precursors) have higher EC efficiency than PCN‐1 (N‐doped) and PCN‐3(pyrene moieties) [124].

##### 3.1.3.2 Sonogashira–Hagihara Coupling

The Sonogashira–Hagihara coupling (commonly referred to as the Sonogashira coupling) occurs when a terminal alkyne reacts with an aryl or vinyl halide to create C≡C covalent linkages, resulting in alkyne‐containing compounds. Same as Suzuki coupling, it is usually conducted at room temperature or slightly elevated temperatures, plus it is suitable for both small‐scale laboratory synthesis and large‐scale industrial applications [125]. In a group study led by Yuan‐Zhao Peng, four types of Bodipy‐based CMPs (CMP‐1 to CMP‐4) were fabricated by introducing anthranyl and perylene polycyclic aromatic hydrocarbons (PAHs) into their structure via the Sonogashira coupling reaction [126]. The coupling reaction happens between ethynyl groups (‐C≡CH) of the 1,3,5‐Triethynylbenzene (TEB), acting as terminal alkynes, and iodo groups of the Bodipy derivatives, forming a three‐dimensional (3D) conjugated network. PAHs act as electron donors, while Bodipy acts as an electron acceptor, and this donor‐acceptor interaction facilitates charge transfer, enhancing photosensitization of CMPs and improving intersystem crossing (ISC) efficiency. Therefore, CMP‐2 and CMP‐4, which contain PAHs, exhibit significantly higher photosensitizing abilities compared to CMP‐1 (the control without PAHs), whose overall photosensitization abilities follow this order: CMP‐4> CMP‐2> CMP‐3> CMP‐1.

##### 3.1.3.3 Ullmann Coupling

**FIGURE 6:**
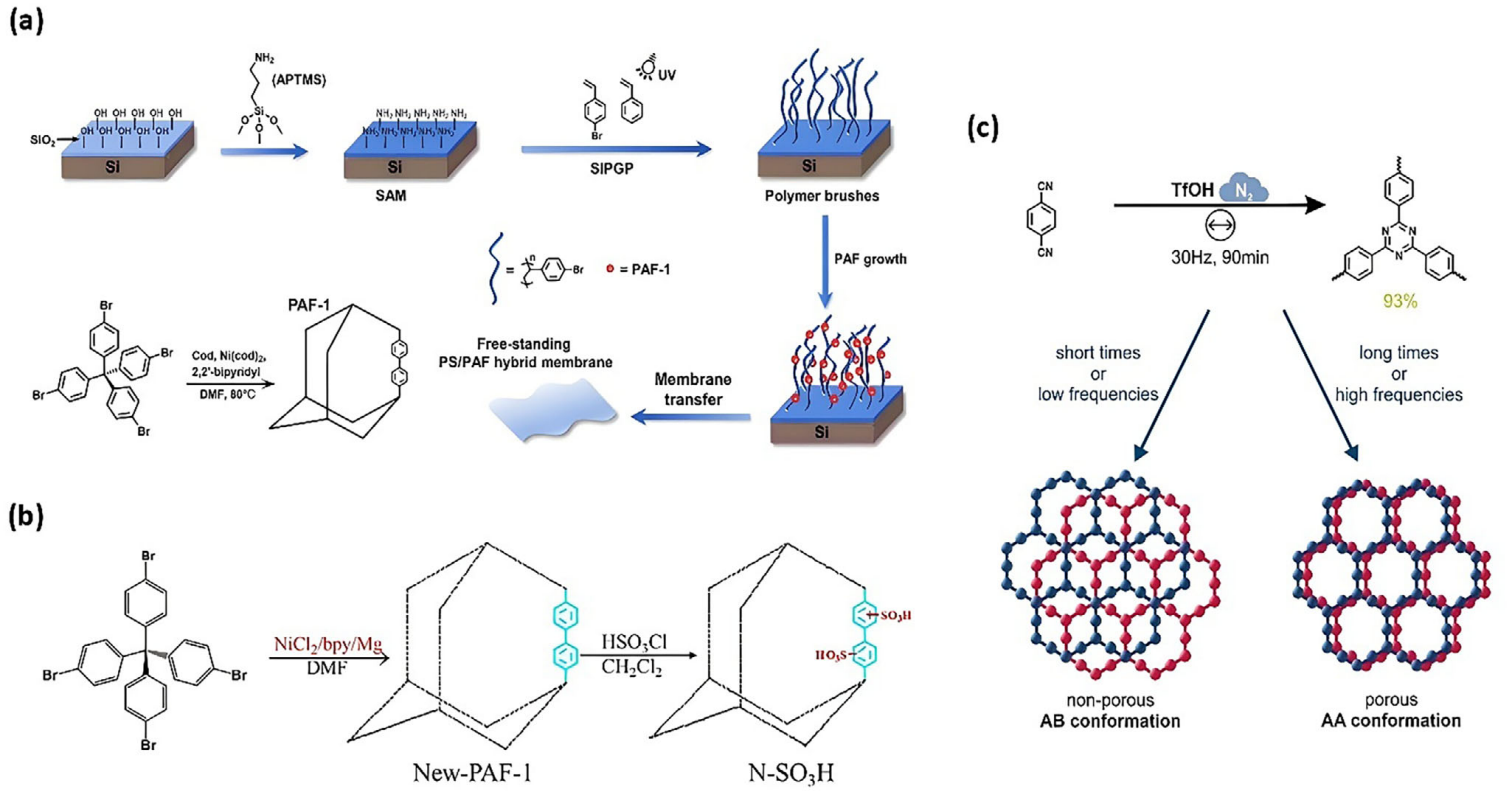
(a) Scheme for the preparation of PS‐Br brushes grafted on silicon substrates, the subsequent PAF growth on the surface, and the fabrication of a hybrid membrane and synthetic routine for PAF‐1. Reproduced with permission from ref. [128]. Copyright 2023, Royal society of chemistry. (b) synthesis of New‐PAF‐1 and N‐SO_3_H via Yamamoto coupling reaction. Reproduced with permission from ref. [130]. Copyright 2022, American Chemical Society. (c) utilizing the mechanochemical reaction of 1,4‐Dicyanobenzene to mechanochemical (mCTF‐1) was found to produce porous and non‐porous material depending on the mechanical energy applied. Reproduced with permission from ref. [133]. Copyright 2024, Wiley‐VCH.

A different mechanism of creating carbon‐carbon bond in a biaryl from two reacting aryl halides, is the Ullman coupling reaction which unlike Yamamoto coupling, copper compounds (e.g., CuI or CuBr) play as a catalyst and a base, often potassium carbonate (K_2_CO_3_) or sodium hydroxide (NaOH) is used in the medium [127]. The Ullmann coupling has been used to synthesize POPs with extended π‐conjugation. For instance, a polymer brush‐directed synthesis strategy for PAFs via surface‐confined Ullmann coupling was developed by Ruihe Yu and colleagues to fabricate free‐standing PS/PAF membranes which merge mechanical flexibility with microporosity [128]. The assembly procedure entails an ordered sequence: at the beginning, 3‐aminopropyltrimethoxysilane (APTMS) self‐assembled monolayers (SAMs) were formed on silicon wafers. Then, UV irradiation (300–400 nm) initiated the growth of poly(4‐bromostyrene) (PS‐Br) brushes on the pre‐functionalized substrate as a reactive template for PAF nucleation. The crowded yet tunable environment of PS‐Br brushes act as a restricted nanoreactors for the PAF growth to sub‐300 nm spheres. After this step, as illustrated in Figure 6a, tetrakis(4‐bromophenyl) methane (TBPM) underwent Ni‐mediated homo‐coupling within the PS‐Br matrix at 80°C to yield PAF‐1 spheres in ∼250 nm range. As bromobenzene groups in PS‐Br chains directly participate in the coupling reaction, the strong integration of PAFs into the brush matrix is ensured. Ultimately, etching the silicon oxide layer with HF yielded flexible, defect‐free membranes (up to 1 cm^2^) with retained porosity.

##### 3.1.3.4 Yamamoto Coupling

In a Yamamoto coupling, two carbon atoms are connected as a result of the reaction between two aryl halides to produce biaryl derivatives or extended polyaryl networks. A nickel (0) catalyst (e.g., Ni(cod)_2_ or Ni (PPh_3_) _4_) is essential to initiate the reaction [129]. In a study leaded by Shanshan Wang, a new HF‐resistant, sulfonic acid‐functionalized PAF‐1 (N‐SO_3_H) using a cost‐effective NiCl_2_/bpy/Mg catalyst system was developed to fulfil the need of recovering electronic special gases (SF_6_, NF_3_, Xe, etc.) from semiconductor waste streams [130]. The reaction mechanism (Figure 6b) follows the reduction of NiCl_2_ to the active Ni (0) by Mg, which catalyzes homocoupling of tetrakis(4‐bromophenyl) methane (TBPM). The reaction proceeds through the insertion of Ni(0) into C–Br bonds, forming aryl‐Ni(II)‐Br intermediates, and ultimately, the coupling of two aryls to form a C–C bond, followed by the regeneration of Ni(0). The brilliance of this protocol lies in replacing Ni (COD)_2_ with a cheap NiCl_2_/Mg reductant system, while maintaining the Yamamoto mechanism's efficiency (yield: 95.2%) for high‐surface‐area polymer synthesis (4076 m^2^/g BET, 5206 m^2^/g Langmuir). Subsequent –SO_3_H functionalization of the framework, enhances the polarity and gas uptake via dipole‐induced interactions, achieving a record in SF_6_ (104.5 cm^3^/cm^3^) and Xe (93.9 cm^3^/cm^−3^) adsorption capacity at 298 K.

### 3.2 Fabrication Techniques

#### 3.2.1 Mechanochemical Synthesis

According to mechanochemistry, mechanical force, typically through ball milling or grinding, has the power to induce chemical reactions between solid precursors, often eliminating the need for solvents or high temperatures. Unlike traditional methods that rely on solvents to operate, which often produces hazardous byproducts, its solvent‐free nature aligns with green chemistry principles, making it an environmentally benign alternative to traditional synthetic routes [131]. Formation of robust covalent bonds in POPs with tailored porosity, high surface areas, and enhanced stability are the most advantageous outcomes of this method [132]. The first successful cyclotrimerization of nitriles to create a CTF using this method was conducted by Stefanie Hutsch and her coworkers in the recent year. This achievement is a very groundbreaking step in the fabrication of CTFs, as they are typically synthesized using solvothermal methods and aligns with the principles of green chemistry as it eliminated the need for any solvents. Different set of monomers were tested in this experiment to examine the versatility of the method, and results prove that using 4‐methylbenzonitrile, 1,4‐dicyanobenzene, and 4,4’‐biphenyldicarbonitrile yields highly porous CTFs, meanwhile, if monomers are functionalized in ortho sites or with more than two functional groups such as 1,3,5‐tricyanobenzene (TCB) or 1,2,4,5‐Tetracyanobenzene, non‐porous polymers or gas formation are observed as product. They also varied the effecting elements of the reaction to see their impact on the conformation of CTFs. It was revealed that low mechanical energy input, either by lowering the reaction time or applying lower frequencies, results to a staggered AB conformation with less porosity, whereas the porous polymers with an eclipsed AA conformation resulted from applying opposite conditions (Figure 6c) [133].

**FIGURE 7:**
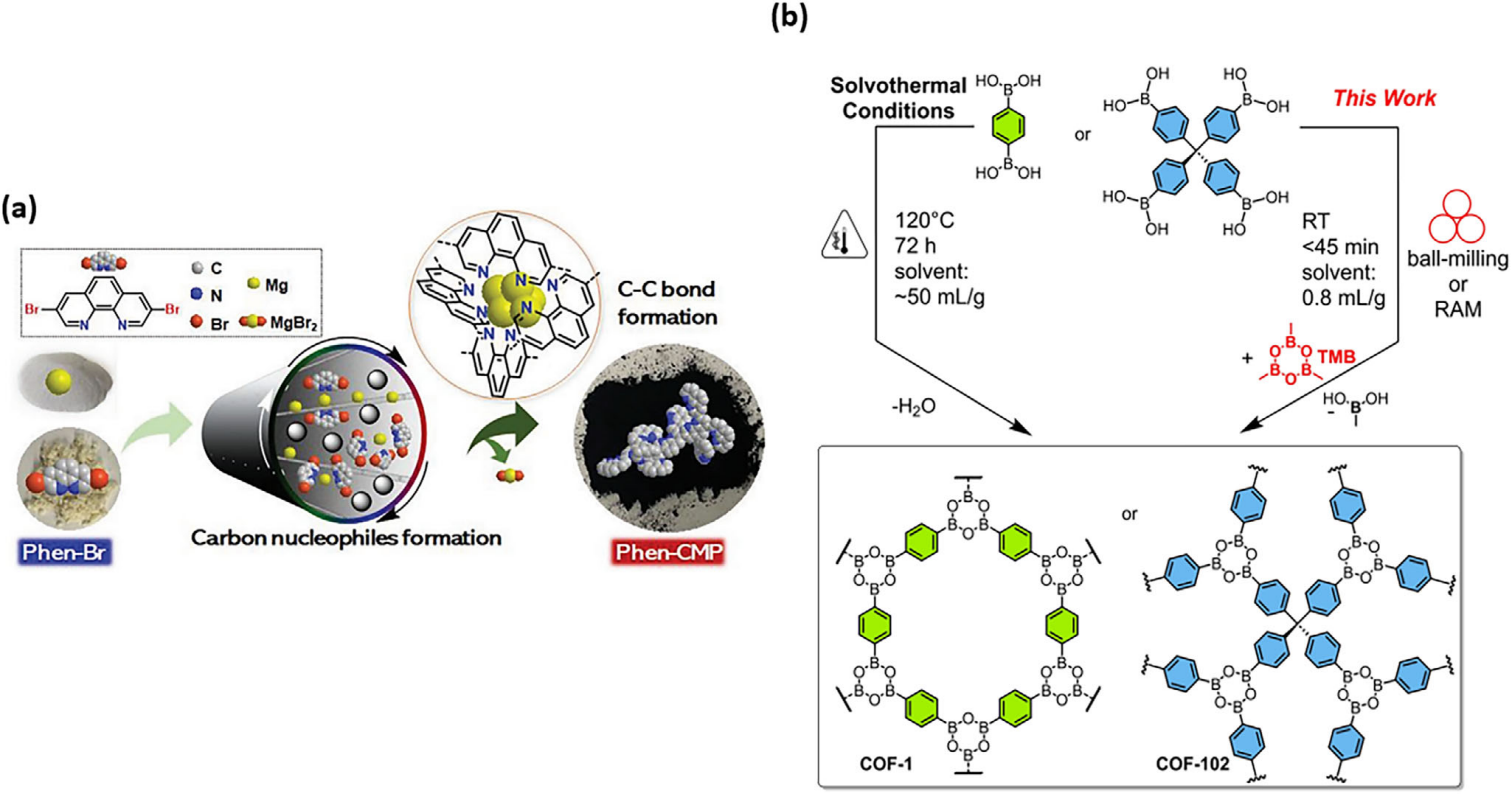
(a) schematic depiction of the Mg‐promoted mechanochemistry‐driven procedure toward the construction of aza‐fused π‐conjugated networks, including the structure of the monomers and corresponding products. Reproduced with permission from ref. [134]. Copyright 2022, Wiley‐VCH. (b) conventional solvothermal vs. herein reported TMB‐mediated mechanochemical synthesis of boroxine COFs, Reproduced with permission from ref. [136]. Copyright 2024, Wiley‐VCH.

Fan et al. [134] achieved a solvent‐free and mild‐condition mechanochemical synthesis that defies conventional constraints. Using 3,8‐Dibromo‐1,10‐phenanthroline (Phen‐Br) or similar linearly structured aromatic bromides with aza‐fused cores, they fabricated CMPs with high nitrogen content (Figure 7a). Priorly, producing CMPs with triazine units often required rigid monomers with multiple reactive sites but in this work, the coordination ability of aza‐fused monomers (e.g., phenanthroline, bipyridine) with magnesium (Mg) was leveraged to form 3D assemblies, which are then polymerized via C–C bond formation. During the experiment, it was observed that changing the Mg to monomer ratio, from the optimized point could lead to lower porosity and decreased surface area. The resulting CMPs boast their exceptional surface areas (up to 789 m^2^/g), hierarchical porosity, and a combination of their micropores and mesopores structure [134]. In some cases, small amounts of liquids are needed to preserve the integrity of structures like crystallinity or enhance the synthetic conditions solid reactants, and it's called liquid‐assisted grinding (LAG) [135]. For example, boroxine‐linked COFs are very hard to synthesize because of the high sensitivity of boroxine linkages (–B_3_O_3_–) to water and the reversibility of the reaction for its near‐unity equilibrium constant. These obstacles had been overcome for the first time by E.Hamzehpoor and his group through adding trimethylboroxine (TMB) as both a water‐scavenging agent and a modulator by removing water and pushing the equilibrium to COF formation, as demonstrated in Figure 7b. Along the TMB, a combination of polar (THF/dioxane) and aromatic (mesitylene) liquids in specific ratios ensures a high level of crystallinity and efficient polymerization.

#### 3.2.2 Microwave‐Assisted Synthesis

**FIGURE 8:**
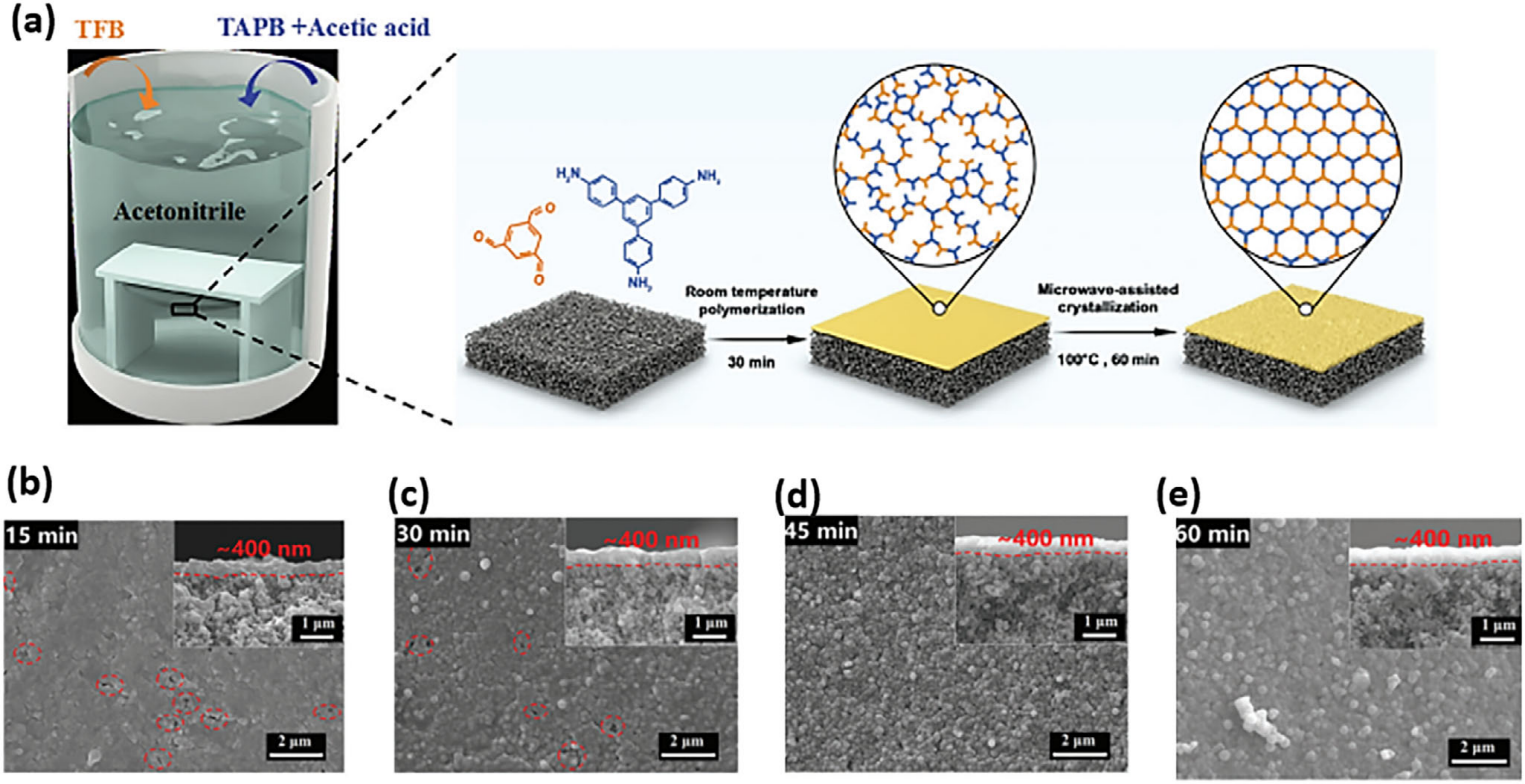
(a) Schematic illustration of the fabrication process of the TFB‐TAPB membrane. (b–e) SEM images of TFB‐TAPB membranes at different microwave treatment times (15–60 min). Reproduced with permission from ref. [138]. Copyright 2025, Wiley‐VCH.

As an energy source, microwave irradiation accelerates reaction kinetics by generating heat, often by orders of magnitude compared to conventional heating. Scientifically, the reason behind it lies in the absorbance of this energy by polar reactants (e.g., monomers, catalysts, or solvents), which leads to the rapid dielectric heating of reactants through molecular friction, providing uniform heating and a lesser number of byproducts. In the field of POPs, microwave‐assisted synthesis is not exclusive to a special group [111]. In a study by Giri et al., an HCP for adsorbing pharmaceutical contaminants from water was prepared via the microwave‐assisted method. The polymerization time of biphenyl groups was reduced from 24 h to an hour. The reaction was carried out in 1,2‐dichloroethene (DCE) as solvent in a closed Teflon tube within a microwave reactor at 50°C for 30 min and 90°C for 30 min. Revealed by FE‐SEM and TEM microscopy, the resultant poly‐biph exhibited merely spherical morphology, with particle sizes in the range of 500–900 nm and considerable tendency to agglomeration [137]. Primarily, microwaves are often thought of as a heating source because they generate heat through dielectric loss, where materials absorb microwave energy and convert it into heat. However, their role goes beyond just heating. By promoting molecular alignment and reducing defects, the microwave field drives amorphous materials toward a thermodynamically stable state. This effect was studied by Xu et al. in a two‐step process of COF fabrication, including in situ polymerization of COF on an Al_2_O_3_ substrate and its further crystallization by microwaves (Figure 8a). At first, on the surface of the Al_2_O_3_ substrate, amine and aldehyde groups were positioned to provide nucleation sites, then it was immersed in a solution mixture of 1,3,5‐Triformylbenzene (TFB) and 1,3,5‐tris(4‐aminophenyl) benzene (TAPB). After half an hour of polymerization at room temperature, microwave irradiation at 100°C for 60 min turned the as‐prepared amorphous pristine membrane into a compact crystalline layer, a strategy known as “disorder‐to‐order”. Figure 8b–e shows the gradual yet quick SEM images of crystallization of the membrane as the reaction time increases [138].

#### 3.2.3 Solvent‐Assisted Methods

Solvent‐assisted methods rely on polar/non‐polar solvents to provide a medium for reactions to happen and consider their serious role in dictating the pore size, distribution, surface area and allowing for modification of POPs with functional groups. These methods are dominantly used in laboratory and industrial scale.

##### 3.2.3.1 Solvothermal Synthesis

**FIGURE 9:**
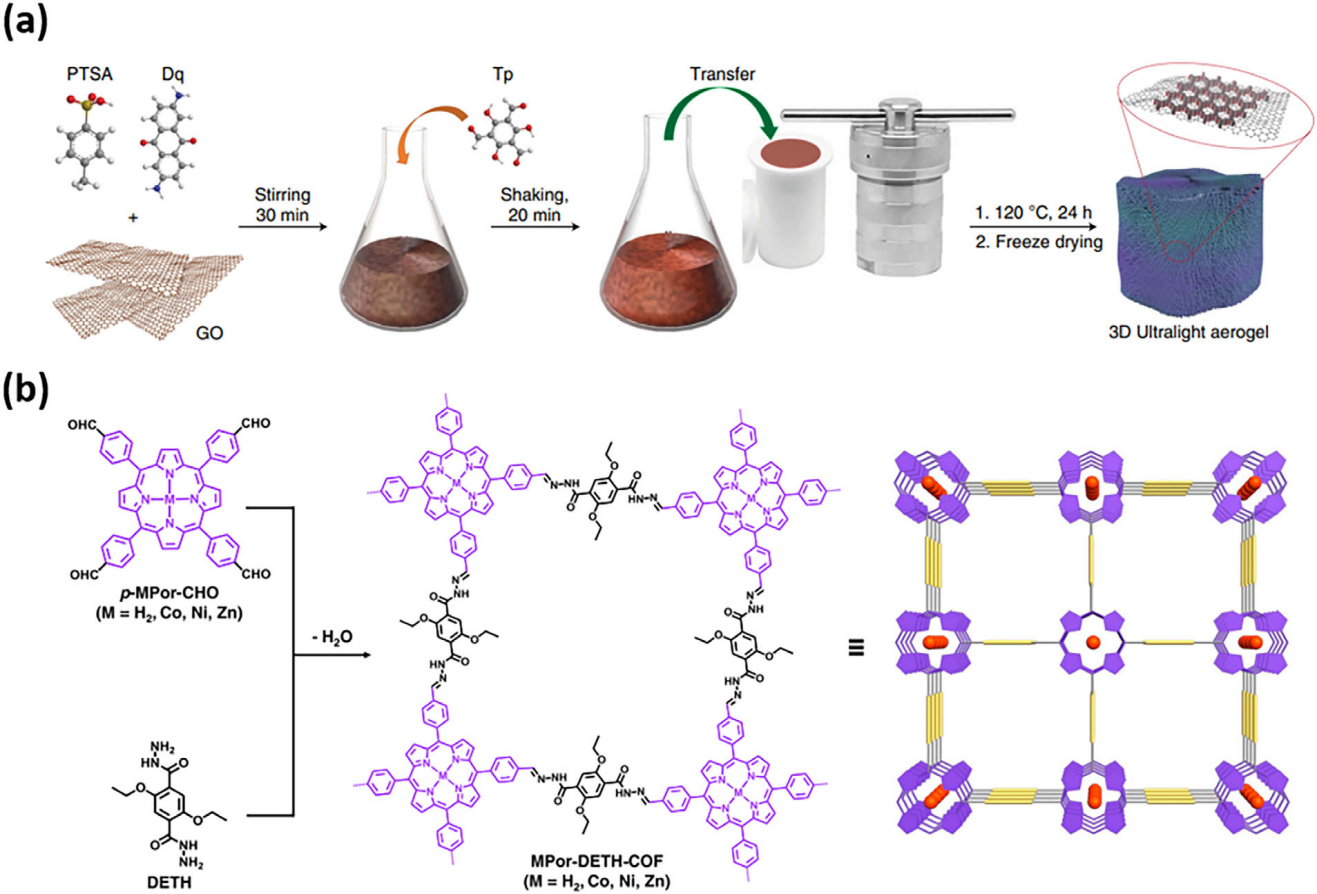
(a) Scheme of the synthetic procedure for the preparation of the COF/rGO aerogel. Reproduced with permission from ref. [139]. Copyright 2020, Nature. (b) schematic representation of the synthesis of MPor‐DETH‐COFs. Reproduced with permission from ref. [142]. Copyright 2021, Nature.

In this method, the reaction of monomers or precursors happens within a solvent medium under high temperatures and pressures, typically inside a sealed autoclave. The solvent serves as a reaction medium, helping to the dissolution of reactants, stabilization of intermediates, and nucleation of polymer networks. The harsh conditions expedite the formation of covalent bonds and more stable crystalline phases. The synthesis of COFs and their composites predominately harness the solvothermal pathway, since it affords integration of COFs with other materials, such as graphene oxide (GO), to create hybrid structures. In a study done by Li et al., the solvothermal method was applied to synthesize TpDq‐COF (a COF formed from 1,3,5‐triformylphloroglucinol (Tp) and diaminoanthraquinone (Dq)) and its composite with reduced graphene oxide (rGO) (Figure 9a) [139]. One more illustration of COF composites involves boron/oxygen/sulfur tri‐doped carbon nanotube (TT‐BOST@CNT) as a sulfur host material, synthesized via the solvothermal method. Carbon nanomaterials like carbon nanotubes (CNTs) and carbon nanofibers are used to host sulfur in lithium‐sulfur batteries due to their large surface area and porous structures, but unfortunately, their non‐polar nature results in weak interactions with polar polysulfides. To this end, CNTs are evenly encapsulated by TT‐BOST‐ a robust COF containing abundant B, O, and S atoms—which provides strong chemisorption sites for polysulfides. TT‐COF@CNT was synthesized from TTBA (thieno[3,2‐b]. thiophene‐2,5‐diyldiboronic acid) and HHTP (2,3,6,7,10,11‐Hexahydroxytriphenylene) monomers in the presence of CNTs in a stainless‐steel autoclave at 150°C for 3 days [140].

Sometimes, the solvothermal conditions, for instance, duration, depending on the small changes in the starting monomers differs from 2 or 3 days to a week. This effect of monomers was observed in the synthesis of four isostructural COFs (MPor‐DETH‐COF, where M = H_2_, Co, Ni, Zn) using a solvothermal method and explore their structure–property–activity relationships in hydrogen evolution reaction (HER) from water [141]. The authors aimed to design COFs from porphyrin aldehydes incorporated with different metal ions (e.g., Co, Ni, Zn) into their porphyrin rings as building blocks to further tune the optoelectronic properties of each COF and its impact on HER performance, as it has been illustrated in Figure 9b. While the general procedure is similar, the reaction conditions (solvent ratios, acetic acid concentration, and reaction time) differ for each COF to account for differences in the solubility, reactivity, and metal coordination properties of the porphyrin precursors.

##### 3.2.3.2 Interfacial Polymerization Method

In a technique known as interfacial polymerization, two immiscible liquids such as water (aqueous phase) and an organic solvent (e.g., hexane, toluene), are contacted with each other, where the dissolved reactants in separate phases diffuse to the interface and undergo a polymerization reaction, forming a thin polymer film or membrane. Unlike homogeneous reactions, which spread crystals evenly in all directions to create bulky, uniform materials, the liquid‐liquid interfacial synthesis method cleverly guides growth to the narrow space between layers, crafting ultra‐thin, delicate films. By focusing the reaction right at the interface, it ensures that molecules assemble in a controlled, one‐directional way, resulting in exquisitely precise, nanoscale structures that are both uniform and remarkably strong [143, 144]. A research group fabricated a self‐standing 3D COF film from hydrophobic diketopyrrolopyrrole derivative (DPP‐CHO) and hydrophilic tetrapod‐amine (TAPM) building blocks at the cyclohexane/acetic acid interface. First solutions of each monomer in dioxane and cyclohexane (as solvent) were added to a vial, followed by the addition of acetic acid as a catalyst and incubation in a vibration‐free environment for 24 h, so the separation of two phases happen. After that, more catalyst is added, and incubation is continued for 5 more days to finally obtain a 13 nm thick film which covers areas of ∼2.5 cm^2^, demonstrating exceptional mechanical strength and flexibility [145]. The interfacial polymerization is often visualized as a binary system between two phases, but it is not confined to such simplicity. Instead, it can transcend this duality and evolve into a multi‐ phase configuration comprised of distinct layers, such as the top organic phase, the aqueous intermediate phase, and the bottom organic phase, each contributing uniquely to the reaction dynamics.

A tri‐phase interfacial polymerization method was employed by Wang et al., to prepare a COF membrane for the elegant separation of monovalent cations from water. This oil–water–oil is composed of a top organic phase (containing diamine monomers with acid groups (e.g., Pa–PO_3_H_2_, Pa–SO_3_H, Pa–CO_2_H)), a middle aqueous phase, which is a pure water, acid, or basic solution, and a bottom organic phase where aldehyde monomers are dissolved. Through this process, monomers diffuse and react in the aqueous phase to form COF nanosheets, which are subsequently assembled into a membrane via filtration. The influence of using amines with acid groups reflects itself in the inner surface of the channels, which are divided into acidic and acid‐free domains. In the former regions, the binding of acidic groups to water molecules to form hydration shells reduces the effective channel size plus their alignment along the channels enhances the sieving ability, while the acid‐free domains maintained high permeability [146]. In interfacial polymerization, the second phase does not necessarily have to be a solvent, whether it could be a gas or air depending on the desired application and material properties.

#### 3.2.4 Electrospinning Methods

Electrospinning is an electrohydrodynamic technique that can be used to fabricate polymer nanofibers. It has recently emerged as a highly useful strategy for the formation and synthesis of POPs [147]. Electrospun polymer nanofibers can be considered as a functional structural form of POPs. In this method, a polymer‐containing solution is subjected to a high electric field, resulting in the formation of a stable jet that is drawn into nanofibers due to electrostatic forces and solvent evaporation. Electrospinning facilitates the formation of one‐dimensional fibrous structures and is particularly effective when combined with in situ polymerization reactions after spinning. These processes result in the formation of permanent porous organic networks while maintaining the fibrous morphology [147, 148, 149]. This process, while not producing continuous fibers, uses similar electrohydrodynamic principles to create particulate or nonfibrous polymer nanostructures. This process produces charged droplets from low‐viscosity solutions that solidify after the solvent evaporates [150]. The electrospinning technique has evolved from traditional single‐fluid methods to more complex multi‐fluid configurations. Single‐fluid electrospinning is the simplest method, in which all components are supplied from a single solution, allowing for uniform distribution of POP precursors in the nanofibers [151]. Coaxial electrospinning uses two concentrically formed fluid streams, allowing for the creation of fiber structures with core–shell designs that have distinct functionalities [152]. Side‐by‐side electrospinning increases structural control by creating Janus‐type fibers with two adjacent phases [153]. Triaxial electrospinning uses three concentric fluid streams to fabricate multilayer nanofibers, allowing for the creation of more complex architectures [154]. Recently, tri‐fluid side‐by‐side electrospinning and other complex electrospinning processes have been developed [155]. These advances allow for the precise spatial programming of multiple functional components within a single fiber. With the development of the electrospinning technique, it is possible to provide flexible and effective POP nanostructures for various applications [156].

### 3.3 Topology and Structure Control

A critical factor in determining the structural geometry and properties is “topology”, and controlling it in the context of POPs and other materials refers to the ability to design and manipulate the spatial arrangement and connectivity of the molecular building blocks that make up the material. This involves figuring how the monomers or nodes are linked together to form a specific network structure, which ultimately determines the material's porosity, surface area, pore size distribution, and functional properties [157]. The existing factors that are fundamental to the topology design of POPs are variant, for instance, the geometry and symmetry of the monomers (e.g., trigonal, tetragonal, tetrahedral) as a major contributing factor could be mentioned. For example, Trigonal monomers often form hexagonal networks, while tetrahedral monomers favor diamond‐like topologies.

#### 3.3.1 Crystalline vs. Amorphous Structures

**FIGURE 10:**
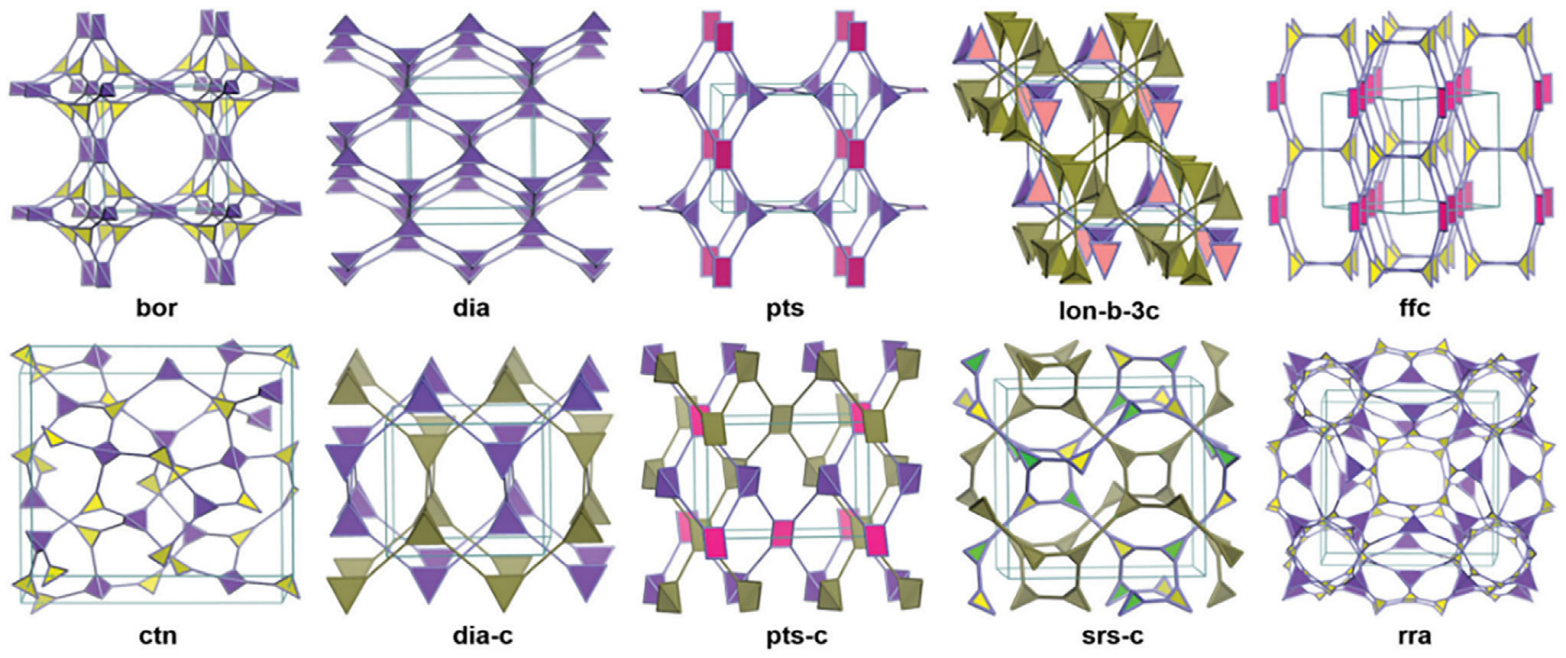
Topologies in 3D COFs. Reprinted with permission from ref. [158]. Copyright 2020, Royal society of chemistry.

Each type of POPs (e.g., COFs, CTFs, HCPs) has its own characteristic topologies, ranging from crystalline and ordered to amorphous and disordered. Looking at different POPs, COFs, and partial number of CTFs own ordered topologies for their crystalline structure, which takes various spatial forms and differs in 2D frameworks comparing to 3D ones. Most common reported topologies of 3D COFs are illustrated in Figure 10 [158]. Regardless of these two groups, in the rest of POPs, because of the amorphous structure, disordered topologies due to the random linking of monomers are dominantly observed, but in very rare cases like PIMs, interpenetrated networks have been detected [94, 158, 159].

#### 3.3.2 Post‐Synthetic Modifications (PSMs)

The chemical modifications performed after initial synthesis enable the incorporation of functionalities that may be incompatible with the harsh conditions often required for the synthesis of POPs. For crystalline POPs, PSMs can introduce new functional groups (e.g., amines, carboxylates, sulfonates, or metal complexes) without disrupting the framework, while for amorphous POPs, PSMs can enhance porosity or surface area. PSMs via covalent bonds are usually permanent and highly stable, whereas in the opposite strategy, non‐covalent bonds are formed through interactions such as hydrogen bonding, *π*–*π* stacking, or electrostatic interactions. Often, to improve compatibility with specific environments or to introduce reactive sites, the outer surface gets refined by techniques such as grafting or coating, depending on the need [160]. A summary of recent POPs, including their structures, functions, and applications, is listed in Table 3.

**TABLE 3:** Summary of recent POPs: Structures, functions, and applications.

Type of POP	Structure	Amorphous/crystalline	Synthesis technique	Functionalization	BET surface area (m^2^. g^−^^1^)	Application	Refs.
Amidoxime‐PIM‐1	3D, hierarchical porous structure	Amorphous	Non‐solvent‐induced phase separation (NIPS)	Amidoxime	356	Removal of uranium from seawater	[161]
Tetrazole‐PIM‐1	3D, microporous polymer with interconnected voids	Amorphous	Solution‐phase polymerization (PIM‐1) + thermal post‐modification (PIM‐Tz at 120°C).	‐CN	431	catalyst binders in high‐temperature PEM fuel cells (HT‐PEMFCs)	[162]
Amidoxime‐PIM‐1	3D, rigid, micropores from inefficient chain packing	Amorphous	Solution‐phase polycondensation (PIM‐1) + thermal post modification (AOPIM‐1 at 69°C)	___	552	Adsorptive removal of charged small molecules from water	[163]
Ketone‐PIM‐1 Alcohol‐PIM‐1 Methylimine‐PIM‐1 Hydrazone‐PIM‐1 Polyethylenimine‐PIM‐1	3D, rigid, contorted backbone	Amorphous	Non‐solvent approach (using a volatile reagent low boiling solvent where PIM‐1 is insoluble)	Methylmagnesium Bromide (Memgbr), Ethylmagnesium Bromide (Etmgbr), Isobutylmagnesium Bromide (I‐Bumgbr), Phenylmagnesium Bromide (Phmgbr)	701 629 776 371 44	Gas adsorption and separation	[164]
NPBI/PIM‐BM	3D Crosslinked network of poly(2,2′‐(1,4‐naphthalene)‐5,5′‐bibenzimidazole) (NPBI) and bromomethylated PIM (PIM‐BM)	Amorphous	Conventional solvent method	___	171 (for PIM‐BM‐70)	High‐temperature proton exchange membranes (HT‐PEMs) for fuel cells	[165]
PAF‐361 PAF‐362 PAF‐363	3D, rigid, donor‐acceptor structure	Amorphous	Solvothermal	___	1124 1619 535	photocatalytic H_2_O_2_ production	[166]
PAF‐AN_x_T_y_‐AO	3D, rigid, multivariate PAF with adsorption and Photocatalytic groups	Amorphous	Conventional solvent method (including Sonogashira coupling + post modification)	___	Not reported	Photocatalytic removal of uranium from water	[167]
PAF‐154 PAF‐155	3D, fully conjugated PAFs with Core Units: PAF‐154: Tetraphenylene PAF‐155: Pyrene	Amorphous	Gilch Reaction (condensation polymerization)	___	610 255	Used in perovskite solar cells for boosting power conversion efficiency (PCE) and device stability	[168]
C‐PAF Si‐PAF	3D, rigid, ultralight, C‐PAF: carbon‐centered monomer and Si‐PAF: silicon‐centered monomer	Amorphous	Yamamoto C─C coupling reaction between trigonal prismatic monomers	___	4857 6099	Hydrogen storage for fuel cells	[169]
PAF‐1	3D, rigid, with diamond‐like topology, abundant benzene rings as polyiodide anchor sites	Amorphous	Yamamoto‐type Ullmann Coupling (C─C bond formation) using Tetrakis(4‐bromophenyl) methane monomer	___	5600	Iodine host in aqueous zinc‐iodine batteries	[170]
PAF‐150 PAF‐151 PAF‐152 PAF‐153	amino acid‐functionalized PAFs with varied 2D and 3D topologies	Amorphous	N−H Insertion Polymerization (Ru‐catalyzed) between Diazocarbonyl + aromatic amine monomers	α‐phenylglycine fragments	168 172 98 165	Biomedical (drug delivery, photodynamic therapy).	[171]
PAF‐XJTU‐1 PAF‐XJTU‐2 PAF‐XJTU‐3 PAF‐XJTU‐4	3D, uniform micropores	Amorphous	Friedel‐Crafts Alkylation between Polyaromatic hydrocarbons + CH_2_Cl_2_ using: AlCl_3_ catalyst.	___	2024.62 1923.78 1498.80 1419.02	Separation and absorption of sulfur hexafluoride (SF6) gas	[172]
PAF‐1‐SO3H	3D, rigid, micropores with interconnected channels	Amorphous	Friedel‐Crafts polymerization of aromatic monomers with crosslinkers + post‐synthetic sulfonation	Chlorosulfonic acid	790	Energy storage in Lithium–Sulfur batteries	[173]
HCP‐NAP HCP‐DPh HCP‐DPM HCP‐DPE HCP‐DPP	3D, containing double‐benzene‐ring building units which alkyl chain length between benzene rings controls structural rigidity vs. flexibility.	Amorphous	Conventional Friedel‐Crafts alkylation between double‐benzene rings with ethylene/propylene linkers.	___	778 891 1158 1031 927	Methane adsorption and storage	[174]
carbazole−bismaleimide‐based‐HCP	3D, rigid, micro and mesopores, electron‐rich pore surface	Amorphous	Radical polymerization of Bis‐Cz(BMI) without metal catalyst	Carbazole, Bismaleimide	57.2	Iodine capture from nuclear waste for Environmental remediation	[175]
benzonquanmine‐HCP‐1 benzonquanmine‐HCP‐2 benzonquanmine‐HCP‐3	3D, hierarchical porosity, N‐rich, π‐conjugated framework, pore size/ distribution tunes with linker rigidity	Amorphous	Friedel‐Crafts alkylation between Benzonquanmine monomers using 1,4‐Bis(chloromethyl)benzene and 9,10‐Bis(chloromethyl)anthracene crosslinkers	Benzonquanmine	24.2 840.6 678.2	Iodine adsorption and removal from water	[176]
Perylene diimide‐HIP‐1 Perylene diimide‐HIP‐2 Perylene diimide‐HIP‐3	3D, solvent‐responsive assembly/disassembly, dynamic porosity with flexible methylene bridges which enable swelling in water	Amorphous	One‐Pot Synthesis (Friedel‐Crafts alkylation and between α, α′‐dibromo‐p‐xylene (DBX) and perylene diimide (PDI) monomers + Quaternization)	α, α′‐dibromo‐p‐xylene (DBX) and a perylene diimide (PDI)	397 233 106	CO_2_ adsorption	[177]
HCP	3D, rigid, micropores structure	Amorphous	Friedel‐Crafts acylation between triptycene (monomer) + 1,3,5‐benzenetricarbonyl Trichloride (crosslinker).	keto groups	889.19	CO_2_ capture and separation, radioactive iodine capture	[178]
N‐phenylphenothiazine‐HCP	3D, interconnected mesopores network	Amorphous	One‐step Friedel‐Crafts reaction (formaldehyde dimethyl acetal (FDA) as crosslinker + N‐Phenylphenothiazine (PTH) as monomer)	N‐phenylphenothiazine.	305	heterogeneous photocatalyst for difluoromethylation/cyclization reactions	[179]
CMP‐based thin film composite (TFC) membranes	3D, interconnected micropores network	Amorphous	Electropolymerization (EP) of 1,3,5‐tri(9H‐carbazol‐9‐yl) benzene (TCB)	Electropolymerization of 1,3,5‐tri(9H‐carbazol‐9‐yl) benzene (TCB)	1389.9	CO_2_ capture and separation	[180]
Ts‐ThTh‐CMP Bi‐ThTh‐CMP Py‐ThTh‐CMP	3D, micro/mesoporous structure with D‐A configuration	Amorphous	Condensation Reaction: Thiazolo[5,4‐d]. thiazole (ThTh, acceptor) + Donor Units (thiophene, biphenyl, pyrene) via reaction with dithiooxamide and aromatic aldehydes (Ts‐Ph‐CHO, Bi‐Ph‐CHO, Py‐Ph‐CHO)	Thiazolo[5,4‐d]. thiazole (ThTh)	70 316 125	Photocatalytic H_2_ production from water	[181]
CTATP‐DHBA TATP‐DHBA	3D, rigid, micro/mesopores structure	Amorphous	wo‐Step Synthesis Schiff‐Base condensation and Pictet‐Spengler cyclization	Pyridine units, Hydroquinone units	341.93 4.27 m2 /g	Removal of uranium from highly acidic nuclear wastewater	[182]
poly(aniline)‐mix (CMPA‐m)	3D, Hybrid micro/mesopores (short linkers for storage, long linkers for fast diffusion	Amorphous	Sequential addition of linkers (short → medium → long) to Br_3_‐TPA (core) and Buchwald−Hartwig coupling reaction to form C−N	Tris(4‐aminophenyl) amine, Phenylenediamine, 1,4‐Diaminodiphenylamine	Various (˷ 500–1100)	Removal of mercury (II) from waste water	[183]
CoPc‐O‐COF CoPc‐S‐COF	Layered 2D, micro/mesopores	Crystalline	Nucleophilic Aromatic Substitution (CoPcF16 (hexadecafluorophthalocyaninato cobalt (II)) + THB (1,2,4,5‐tetrahydroxybenzene)/ BTT (1,2,4,5‐benzenetetrathiol))	1,2,4,5‐Benzenetetrathiol (BTT)	183 285	H_2_O_2_ production	[184]
COF‐LZU1 film COF‐LZU1 Particles	2D, asymmetric structure combining an amorphous network states with crystalline porous states	crystalline	Modulator‐Solvent Strategy using benzoic acid as a modulator to slow imine bond formation, enabling control over crystallization	Benzoic acid, Benzophenone (BP), L (+)‐Tartaric acid (L(+)‐TA), Grubbs‐II catalyst	121 410	Gas Separation/Membranes	[185]
TTF‐COF TPE‐COF	Vinyl‐linked COFs (VCOFs) with orthorhombic pore networks and eclipsed (AA) stacking layers with an interlayer distance	crystalline	Solvothermal [C4 + C2]. Knoevenagel condensation using tetrazine, TTF (tetrathiafulvalene), and TPE (tetraphenylethylene) building blocks	Tetraformyl tetrathiafulvalene (TFTTF), Tetraformyl tetraphenylethylene (TFTPE)	1743 m^2^/g 1840	Gold recovery from e‐water, photocatalysis	[186]
TPDA‐TDTA‐COF TPDA‐BT‐COF TPDA‐ViBT‐COF	2D, rigid, micropores, eclipsed (AA) stacking	crystalline	Solvothermal Knoevenagel condensation of tetra‐amine (TPDA) with tetra‐aldehyde monomers (TDTA, BT, ViBT)	Bis(4‐aminophenyl)‐[1,1′:2′,1″‐terphenyl]‐4,4″‐diamine (TPDA), ((((1E,1’E)‐benzo[c][1,2,5]. thiadiazole‐4,7‐diylbis(ethene‐2,1‐diyl)) bis(4,1‐phenylene)) bis(azanetriyl)) tetrabenzaldehyde) (VIBT)	1040 1230 1610	Cancer Immunotherapy	[187]
CuSA/Ace‐COF CuSA/Obq‐COF	2D, imine‐linked COFs with eclipsed (AA) stacking, microporous	crystalline	Schiff‐base & Knoevenagel condensation of triazine‐based tetra‐amine (TTA) with acenaphthenequinone (Ace) and o‐Benzoquinone (Obq) linkers+ anchoring atomically dispersed Cu single‐atoms (CuSA) onto COFs.	Acenaphthene, o‐benzoquinone, single‐atom Cu	Not reported	Removal of organic pollutants from water	[188]
COF‐300	3D, diamondoid (dia) topology with 7 interpenetrated networks.	crystalline	Solvothermal Schiff‐base condensation (imine linkage)	___	Not reported	selective adsorption and separation of gases and fluids	[189]
3D CTF‐TPM 3D CTF‐TPA	3D network with 4‐connected tetrahedral nodes and 3‐connected triazine rings, ctn (cubic carbon nitride	crystalline	Schiff base formation, Michael addition, and irreversible cyclization/dehydrogenation in DMSO/o‐DCB (1:2) with Cs_2_CO_3_catalyst	Re (CO)_5_Cl, Mn (CO)_5_Br	2002 1804	Gas Storage/Separation	[190]
CTF‐BP CTF‐FL	2D, layered 2D sheets with interlayer spacing,	crystalline	Cyclotrimerization of 1,1′‐biphenyl‐4,4′‐dicarbonitrile (BP‐CN) and 9H‐fluorene‐2,7‐dicarbonitrile (FL‐CN)	___	686 818	photocatalytic H_2_O_2_ evolution	[191]

## 4 Functionalization of POPs

### 4.1 Pre‐Synthetic Functionalization

The pre‐synthetic modification of POPs involves altering the monomers prior to polymerization to add specific chemical groups, which improves the properties of the final polymer [65]. This technique allows for precise management of the structure and functionality of the resulting POP, making it suitable for targeted applications such as gas storage, catalysis, or drug delivery [192]. Common techniques employed include functional group modification, protection/deprotection, and click chemistry to introduce functional sites like –NH_2_, –OH, or azides [193]. Pre‐synthetic modification yields greater stability, uniformity, and enhanced performance in applications compared to PSMS. These methods facilitate the design of POPs with specific reactivity, porosity, and surface interactions for specialized applications.

**FIGURE 11:**
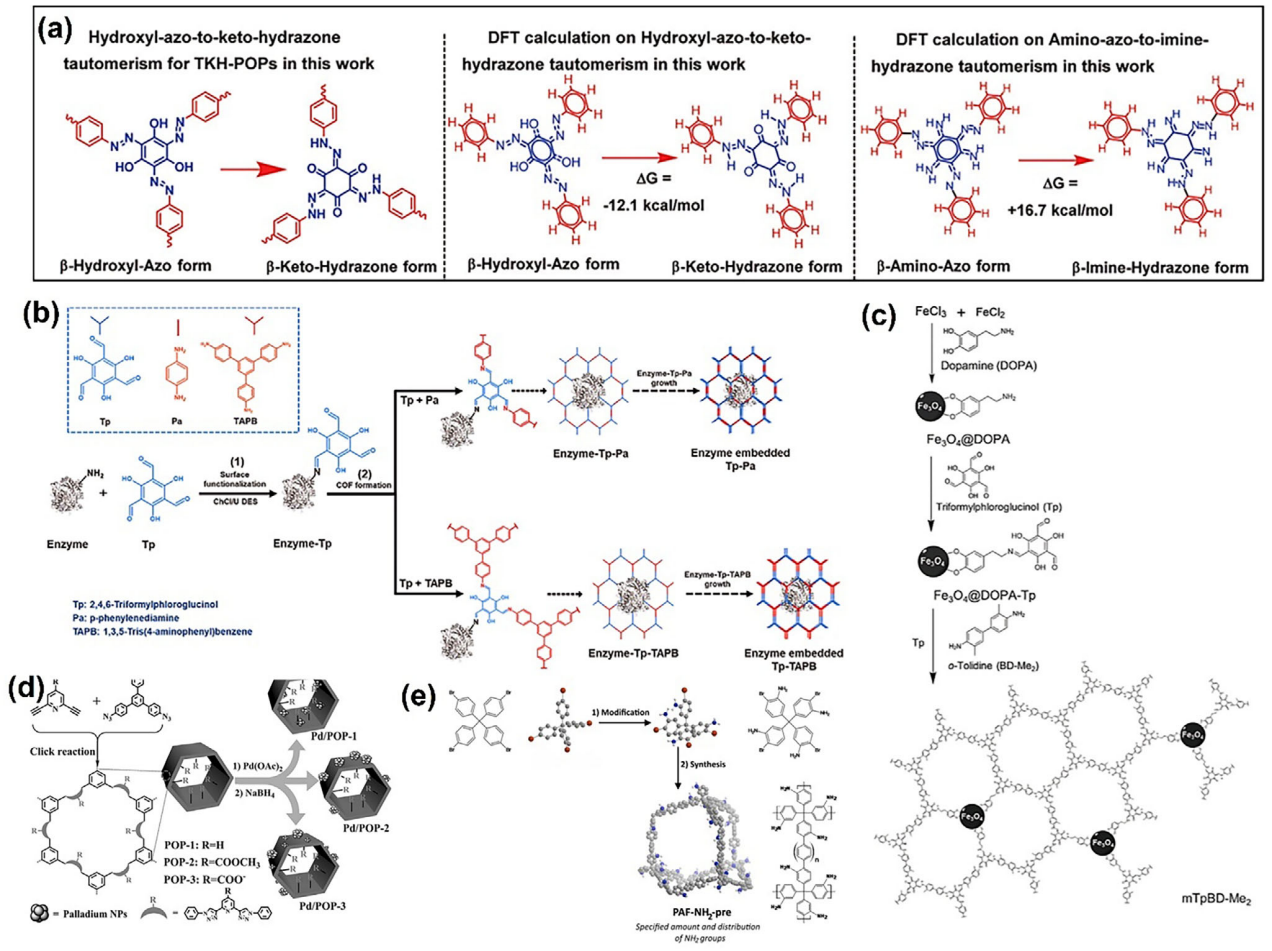
(a) The irreversible conversion of tris(b‐hydroxyl‐azo) benzene to tris(b‐keto‐hydrazo) cyclohexane tautomer is intended for TKH‐POPs. Reproduced with permission from ref. [194]. Copyright 2020, Royal Society of Chemistry. (b) Preparation of enzyme@COF fabrication utilizing ChCl/U deep eutectic solvent. Reproduced with permission from ref. [198]. Copyright 2023, Elsevier. (c) Synthesis of mTpBD‐Me2. Reproduced with permission from ref. [196]. Copyright 2023, Royal Society of Chemistry. (d) Preparation of POP‐1, POP‐2, POP‐3, Pd/POP‐1, Pd/POP‐2, and Pd/POP‐3. Reproduced with permission from ref. [195]. Copyright 2016, Wiley‐VCH. (e) Preparation of PAF‐20‐NH_2_‐pre. Reproduced with permission from ref. [199]. Copyright 2022, Elsevier.

The researchers created TKH‐POPs using a method that involves pre‐functionalization and irreversible tautomerization. The synthesis process begins with two key pre‐functionalized monomers: aromatic diamines, which are transformed into bis(diazonium) salts featuring reactive –N_2_^+^ groups, and phloroglucinol, a trihydroxybenzene compound rich in electron‐donating –OH groups. These functionalized monomers utilize azo‐coupling polymerization to generate a tris(β‐hydroxyazo) benzene framework (Figure 11a). This structure then undergoes an automatic and irreversible tautomerization, rearranging the –OH and –N = N– (azo) bonds to form β‐keto‐hydrazo units (–NH–N = C–) within a cyclohexane‐like subunit. Utilizing these functionalized monomers, the researchers created distinctive frameworks incorporating specific polar groups (keto‐hydrazo) in the backbone, significantly increasing the VOC adsorption capacity. Adding polar keto‐hydrazo groups directly into the polymer framework enhances the chemical environment within the pores, leading to improved interactions with guest molecules. As a result, the TKH‐POPs demonstrate exceptionally high BET surface areas, ranging from 398 to 639 m^2^/g, along with adjustable pore size distributions encompassing ultramicropores, micropores, and mesopores. The twisted conformations of specific monomer units, such as fluorene derivatives, further improve control over pore architecture. Regarding their applications, these functionalized TKH‐POPs show remarkable efficacy in adsorbing VOCs. Notably, TKH‐POP‐1 achieves a record‐high acetonitrile vapor adsorption capacity of 66.3 mmol/g at 273 K, exceeding all previously reported porous materials. It also demonstrates significant uptake of ethanol (22.2 mmol/g) and benzene (18.2 mmol/g), underscoring the synergistic effects of the polymer's porosity and the polar functional groups introduced via pre‐functionalization [194].

In a separate study, Zhong et al. describe the creation of pre‐functionalized POPs that effectively support palladium nanoparticles (Pd NPs), aiming to improve catalytic performance, especially in dehalogenation reactions. They synthesized three different types of POPs: POP‐1, POP‐2, and POP‐3, which contain hydrogen (H), methoxycarbonyl (COOMe), and carboxylate (COO^−^) groups at the 2,6‐bis(1,2,3‐triazol‐4‐yl) pyridyl (BTP) units, respectively (Figure 11d). A significant element of the synthesis process is the pre‐functionalization, which entails adding the COOMe and carboxylate groups to the BTP units before introducing the Pd NPs. The COOMe group functions as an electron‐withdrawing unit, while the carboxylate group (COO^−^) acts as an electron‐donating ligand with strong coordination capability to metal species. These functional groups are incorporated through click chemistry and hydrolysis reactions. The COOMe group creates ester bonds (C═O), while the carboxylate group forms ionic or coordination bonds with Pd NPs, thus stabilizing the nanoparticles and improving their dispersion. Following the incorporation of palladium, the BET surface areas decline, which can be explained by partial pore filling and mass increase due to the attached Pd NPs. POP‐1 maintains its microporous nature after Pd loading, but POP‐2 and POP‐3 transition to mesoporous structures. This reduction in surface area primarily results from pore blockage caused by the functional groups and Pd NPs, leading to a conversion from microporous to mesoporous characteristics, especially in the COOMe and COO^−^ functionalized POPs. The prepared Pd/POP catalysts were tested for their catalytic efficiency in dehalogenating chlorobenzene to benzene. Among the three catalysts, Pd/POP‐3 demonstrated the highest catalytic efficiency and recyclability, achieving complete conversion in 20 h at 25°C. The enhanced performance of Pd/POP‐3 is linked to the electron‐donating influence of the carboxylate group, which elevates the electron density of Pd^0^ species, thereby facilitating the oxidative addition of C–X bonds [195].

Historically, COF composites are formed by initially modifying solid substrates, like magnetic nanoparticles, with particular functional groups, typically amino or aldehyde groups that serve as attachment sites for the development of COFs. This pre‐functionalization phase is essential for maintaining the composite's crystallinity and structural stability. Nonetheless, the extra purification steps also add considerable time, expense, and complexity. To overcome these challenges, Romero et al. created a streamlined synthetic method that negates the requirement for prior functionalization of the magnetic substrate. They employed dopamine‐functionalized magnetic nanoparticles (Fe_3_O_4_@DOPA), which possess surface‐exposed amino groups (–NH_2_). First, they modified the magnetic nanoparticles (Fe_3_O_4_) with dopamine (DOPA) to incorporate these amino groups. Next, these amino groups were treated with a sub‐stoichiometric amount of a diamine monomer (o‐tolidine, or BD‐Me_2_) combined with an excess of an aldehyde monomer (1,3,5‐triformylphloroglucinol, or Tp). The surplus Tp reacts in situ with the amino groups on the DOPA‐coated nanoparticles, establishing imine bonds (─C═N─) as nucleation sites for developing COFs. These imine bonds subsequently undergo tautomerization to form stable β‐ketamine linkages, known for their robustness in COFs (Figure 11c). The COF composite demonstrated a BET surface area of 772 m^2^/g, considerably more significant than the analogous material created using traditional pre‐functionalization techniques. Furthermore, TG analysis revealed a high organic content of 81% and thermal stability reaching around 400°C. Magnetic assessments confirmed the superparamagnetic properties of the material, enabling easy separation through an external magnet. The researchers showcased the COF composite's ability to absorb okadaic acid (OA), a hydrophobic marine toxin linked to harmful algal blooms (HABs). The material exhibited an adsorption efficiency of 71% at 10 µM OA, with uptake rising at higher concentrations, reaching up to 61 mg/g. The material maintained over 84% of its adsorption capacity after three successive adsorption/desorption cycles using 2‐propanol as the eluent. This underscores its reusability and stability in water environments, positioning it as a promising candidate for environmental monitoring and water purification, specifically for detecting and capturing marine biotoxins in seawater [196]. The same research outlines the creation of a recyclable magnetic COF composite called mTpBD‐Me_2_. This advancement includes the pre‐modification of magnetic nanoparticles (Fe_3_O_4_) with dopamine (DOPA) to produce Fe_3_O_4_@DOPA nanoparticles. Dopamine acts as a capping ligand during the co‐precipitation of Fe_3_O_4_, which results in the introduction of primary amino groups (‐NH_2_) onto the nanoparticles' surface. The interaction between dopamine and Fe_3_O_4_ is established via Fe–O bonds formed between the dopamine's catechol groups and the iron oxide core. After this initial modification, the primary amino groups interact with terephthalaldehyde (Tp) to generate imine bonds (C═N) between the amino groups and the aldehyde groups of Tp, which promotes the attachment of COF building blocks. The BET surface area for the optimized composite, 0.2‐mTpBD‐Me_2_, was recorded at 538 m^2^/g, which is similar to the bulk COF's surface area of 468 m^2^/g. The pore size distribution indicates a maximum pore size of around 1.1 nm. When utilized as an adsorbent for marine biotoxins, mTpBD‐Me_2_ showed exceptional effectiveness. The composite revealed maximum adsorption capacities of 812 mg/g for OA and 830 mg/g for DTX‐1, indicating approximately 500‐fold and 300‐fold enhancements, respectively, compared to standard commercial resins like HP20. Its rapid adsorption kinetics allowed for equilibrium to be reached in just 60 min, a factor linked to the composite's elevated surface area and structured pore design, which aids the quick diffusion of biotoxins into the pores. Furthermore, the Tp groups enhance the high adsorption capacity through hydrogen‐bond interactions with the target molecules [197].

Enzyme encapsulation has predominantly faced challenges due to the limited pore sizes of COFs, which restrict the movement of larger enzyme molecules into the already formed structures. The advancement made by Talekar et al. in this research pertains to the pre‐modification of the enzyme surface, specifically glucose oxidase (GOx), by utilizing aldehyde‐containing COF monomers, like 1,3,5‐triformylphloroglucinol (Tp). This aldehyde modification is accomplished through Schiff‐base condensation that occurs between the aldehyde groups of Tp and the amino groups present on the enzyme surface. The modified enzyme (GOx‐CHO) then acts as a nucleation point for the growth of COF networks in situ, facilitated by further Schiff‐base condensation with diamine linkers such as p‐phenylenediamine (PPD) or TAPB (a triamine), all within an environmentally friendly solvent system constituted of a deep eutectic solvent made from choline chloride and urea. This method results in the formation of imine bonds (C═N) between Tp and Pa/TAPB, enabling the concurrent covalent bonding of the COF network to the previously modified enzyme surface (Figure 11b). The reversible nature of Schiff‐base chemistry allows for structural adjustments and Ostwald ripening, aiding in the development of various stable COF morphologies, including fibers, petals, and hollow spheres. This novel approach permits enzyme embedding, regardless of COF pore size, thus overcoming traditional size constraints while retaining high catalytic activity (up to 62% for GOx). Moreover, this technique facilitates high enzyme loading (up to 40%) and immobilization efficiency (up to 90%), significantly surpassing conventional methods of post‐synthesis adsorption. The COFs largely maintained their porosity and crystallinity, exhibiting only a slight decrease in BET surface area (from 295 to 284 m^2^/g for Tp‐Pa COF and from 364 to 349 m^2^/g for Tp‐TAPB COF). Both pore size and volume showed only minor reductions, suggesting that enzyme embedding took place without obstructing the internal COF pores, thereby preserving effective substrate accessibility. In conclusion, the enzyme‐embedded COFs exhibited significant antibacterial properties by generating hydrogen peroxide (H_2_O_2_) through the GOx‐catalyzed oxidation of glucose. These composites achieved more than 99% inhibition of both Staphylococcus aureus and Pseudomonas aeruginosa, and they retained their bactericidal effectiveness after five reuse cycles, illustrating their durability and reusability [198]. Makeeva et al. demonstrated an effective strategy for pre‐functionalizing PAFs by integrating amino groups into PAFs during the monomer stage before polymerization. The team created an amino‐functionalized monomer known as tetrakis‐(4‐bromo‐3‐aminophenyl) methane. This monomer underwent Suzuki cross‐coupling with either 1,4‐phenylenediboronic acid or 4,4'‐biphenyldiboronic acid to form the PAFs (Figure 11e). Consequently, two pre‐functionalized frameworks were produced: PAF‐20‐NH_2_‐pre and PAF‐30‐NH_2_‐pre. A significant benefit of this approach is the accurate and uniform distribution of amino groups, which are covalently incorporated into the polymer backbone during synthesis. This method contrasts with conventional post‐functionalization techniques, which frequently resulting in a random distribution of functional groups and partial obstruction of pores. The pre‐functionalized PAFs displayed increased porosity, greater accessibility, and enhanced interactions with metal nanoparticles. These characteristics ultimately led to improved catalytic selectivity and stability in hydrogenation reactions [199].

### 4.2 Post‐Synthetic Functionalization

The intricate surface chemistry and elevated porosity of POPs provide considerable opportunities for PSMS, improving their properties and functionalities. POPs are synthesized by polymerizing organic monomers, leading to a rich surface of organic functional groups. Because of insufficient conversion in organic reactions, specific polymerization sites on the monomers may stay unreacted and end up included in the POPs [200, 201, 202]. Furthermore, specific organic monomers possess inactive side groups during the polymerization process, enabling these groups to be passed on to the resulting POPs. The residual organic groups on the POPs can serve as binding sites for modifications after synthesis [203, 204]. In addition, the porous nature of the POPs allows for the incorporation of guest molecules through a process termed impregnation, offering a different approach for modification. Consequently, chemical tethering and physical impregnation are the primary methods employed to alter POPs [205, 206, 207, 208]. We will examine three approaches for post‐synthetic modification: Functionalization through organic reactions, Pore surface modification, and Surface alteration.

#### 4.2.1 Functionalization Through Organic Reactions

Covalently connecting well‐known to POPs leads to the development of a functionalized material that not only retains its original properties but also exhibits enhanced stability and selectivity due to additional synergistic effects. Effective functionalization through covalent bonds necessitates available reactive sites for further reactions, which can be divided into two distinct types. The first type involves the deliberate creation of anchoring sites within the framework that were not part of its initial construction. The second type entails reactions occurring at bonding defect sites after the framework has been established [209, 210, 211].

**FIGURE 12:**
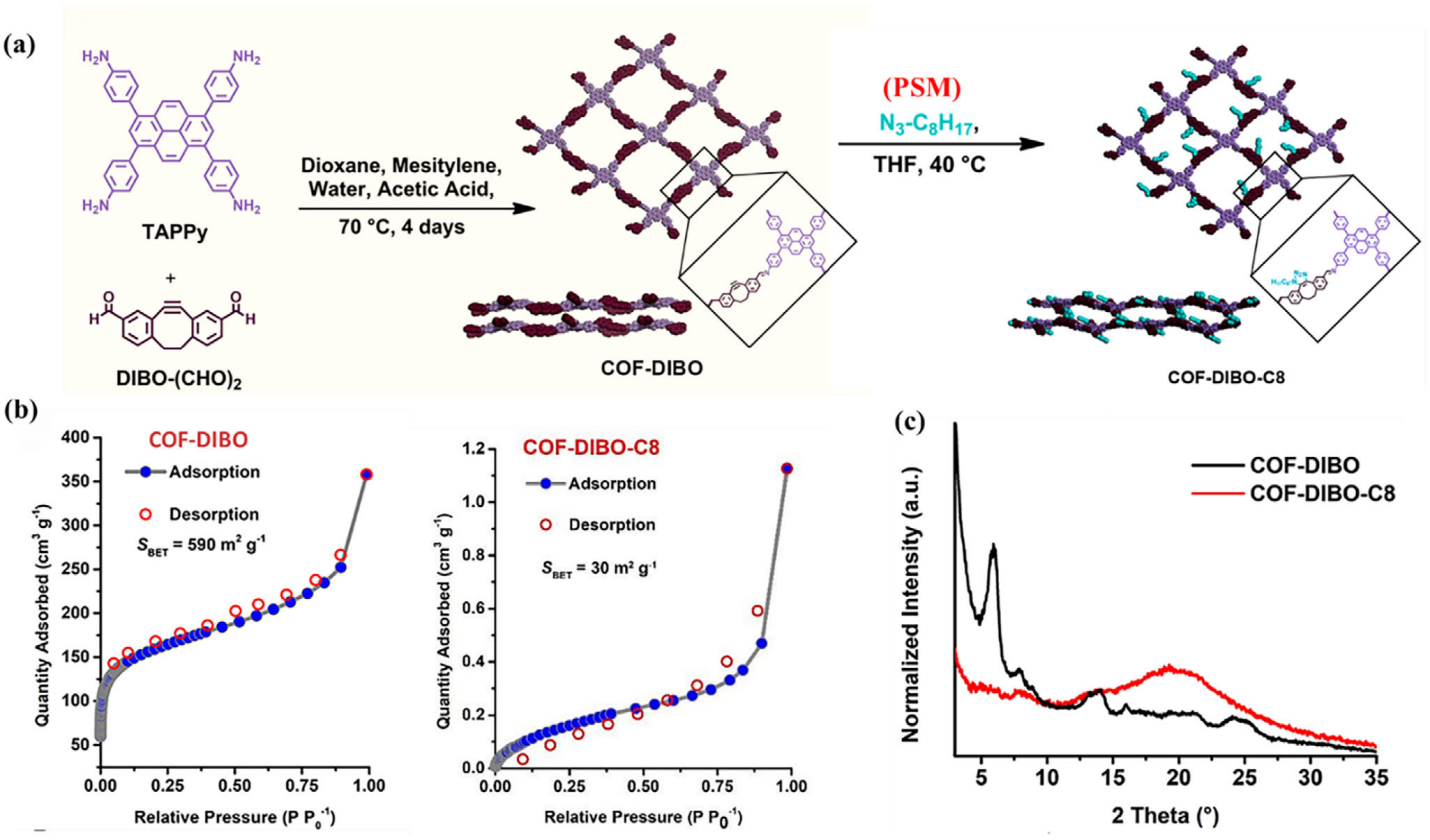
(a) Schematic illustration showing the preparation of COF‐DIBO and PSM of COF‐DIBO‐C8. (b) The isotherm of COFs for nitrogen adsorption is represented by blue dots, while red circles indicate desorption. (c) PXRD pattern of COFs. Reproduced with permission from ref. [202]. Copyright 2021, American Chemical Society.

Li et al. developed a two‐dimensional COF‐DIBO by linking dibenzocyclooctyne with imine using 2,7‐dialdehydedibenzocyclooctyne (DIBO‐(CHO)_2_) and 1,3,6,8‐tetrakis(4‐aminophenyl) pyrene (TAPPy). This synthesis undergoes strain‐promoted azide‐alkyne cycloaddition (SPAAC) reactions without a catalyst, under mild and dilute conditions. Since the alkyne triple bond is still present in the dibenzocyclooctyne (DIBO) structure, post‐synthetic modification is executed through the SPAAC reaction with octyl azide, resulting in the formation of COF‐DIBO‐C8 (Figure 12a). The crystallinity of COF‐DIBO diminishes upon modification to COF‐DIBO‐C8, as evidenced by the disappearance of diffraction features in X‐ray diffraction analysis (Figure 12c). Additionally, the porosity is significantly reduced, with the BET surface area dropping from 590 m^2^/g for COF‐DIBO to just 30 m^2^/g for COF‐DIBO‐C8. Although COF‐DIBO‐C8 loses porosity, it gains flexibility, which can be advantageous in specific applications such as membranes or coatings (Figure 12b). The modified COF also loses its interlayer interactions, leading to increased solubility in organic solvents, which facilitates dispersion and processing. This is particularly beneficial for applications that require solution‐processable COFs. COF‐DIBO‐C8 still retains reactive sites that allow for further chemical modifications beyond the initial reaction with octyl azide. This modularity makes it a more effective platform for customized applications in areas such as sensing, catalysis, or drug delivery, compared to the unmodified structure [212].

**FIGURE 13:**
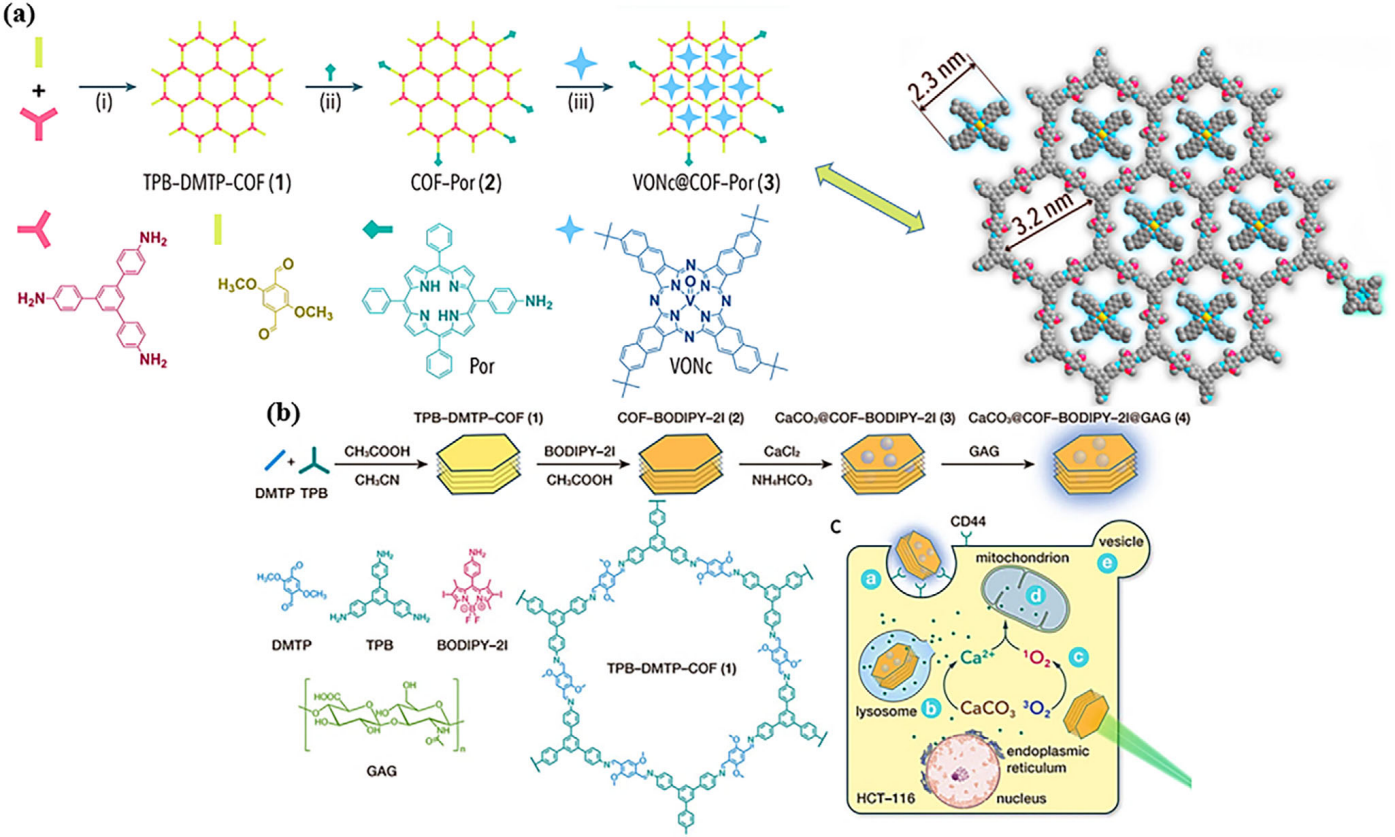
(a) The synthesis of VONc@COF‐Por. Reproduced with permission from ref. [214]. Copyright 2019, American Chemical Society. (b) A diagrammatic representation of the synthesis and BDF of COF and its use in cancer treatment. Reproduced with permission from ref. [215]. Copyright 2020, Wiley‐VCH.

The covalent bonding defect functionalization (BDF) method is among the most frequently utilized PSM techniques for POPs. As POPs are formed through the condensation of dipodal, tripodal, or multipodal monomers, reactive functional groups are left at the ends of the POPs. This allows for the generation of new bonds at these terminal sites, providing an alternative PSM approach that does not disrupt the pore environment. This method of surface modification preserves the internal pore dimensions of traditional POPs, as it takes place on the exterior rather than inside the pores [212, 213, 214, 215]. Guan et al. created a nanoscale (NCOF) intended to function as a dual‐modal agent for both photodynamic therapy (PDT) and photothermal therapy (PTT) in the treatment of cancer. They began by synthesizing TPB‐DMTP‐COF through a Schiff‐base condensation reaction involving 1,3,5‐tris(4‐aminophenyl) benzene and 2,5‐dimethoxyterephthaldehyde. Next, they covalently attached a photosensitizer based on 5‐(4‐aminophenyl)‐10,15,20‐triphenyl porphyrin (Por) to the COF. This modification allowed for the effective production of singlet oxygen (^1^O_2_) when exposed to red light, thereby improving the efficacy of PDT. To enhance this procedure, BDF was utilized, enabling the attachment of porphyrin molecules to the COF while preserving its pore structure. In the final step, vanadyl 2,11,20,29‐tetra(tert‐butyl)‐2,3‐naphthalocyanine (VONc) was incorporated into the framework, resulting in the formation of VONc@COF‐Por via a guest encapsulation method (Figure 13a). This phase resulted in a significant decrease in surface area due to the physical embedding of VONc molecules within the pores, which reduced the pore diameter from 3.2 to 2.5 nm, leaving the BET at 115 m^2^/g. The surface modification achieved through BDF enhanced light absorption, biocompatibility, and the stability of the therapeutic agents in biological environments, marking this study as a promising advancement in nanomedicine for breast cancer therapy, highlighting a biocompatible, metal‐free, and highly efficient system for combined PDT and PTT [214]. Another group of researchers created an nCOF material employing a strategy for synergistic tumor treatment. The foundational COF, TPB‐DMTP‐COF, was produced through the condensation of TPB (1,3,5‐tris(4‐aminophenyl) benzene) and DMTP (2,5‐dimethoxyterephthalaldehyde) via the formation of imine (─C═N) bonds. This synthesis resulted in a BET surface area of 1400‐1600 m^2^/g, with pore sizes between 2.5 and 3.2 nm. Amino‐functionalized BODIPY‐2I underwent a reaction with the COF through imine condensation, establishing C═N bonds between the COF's unreacted aldehyde groups and the amino groups of BODIPY‐2I. This led to the creation of COF‐BODIPY‐2I, which enabled the generation of singlet oxygen (^1^O_2_) for photodynamic therapy (PDT). The linking of BODIPY‐2I partially obstructed some of the pores, resulting in a reduced available surface area; nonetheless, the overall porosity remained relatively high, permitting additional modifications. The BET surface area subsequently declined to 800–1000 m^2^/g. To complete the COF structure, it was immersed in a CaCl_2_ solution and reacted with NH_4_HCO_3_, resulting in the creation of CaCO_3_ nanoparticles within the COF structure (Figure 13b).

#### 4.2.2 Pore Surface Modification

The modification of the pore surfaces in POPs generally involves altering the polymer's surface to improve its characteristics for targeted applications. A successful approach is to incorporate various substances such as metal ions, drugs, and enzymes, which can enhance biocompatibility, provide therapeutic benefits, and boost catalytic activity in the polymers [216]. Various chemical reactions can be used for modifications that introduce particular functional groups or enhance the interaction between the POP and other substances or materials.

Post‐synthetic metalation of POPs with accessible chelating groups can incorporate softer, low‐valent ions and metals. This procedure results in materials possessing unique and valuable structures. Notably, certain POPs include free heteroatom‐containing motifs capable of acting as metal‐chelating groups. This enables post‐synthetic metalation to produce uniform and well‐defined edges with metallation [216, 217]. Doremus et al. reported the catalytic desulfurization capabilities of a nickel‐doped dehydrobenzoannulene‐based POP (Ni‐DBA‐TMT‐POP). It was first synthesized by employing an olefin‐linkage polymerization method, using dehydrobenzoannulene (DBA) to offer π‐conjugation and metal‐coordination sites, along with 2,4,6‐trimethyl triazine (TMT) to improve structural stability and porosity. The final product is a dark yellow powder with an average pore size of 1.7 nm. The polymer was then metallated with a nickel (0) 1,5‐cyclooctadiene complex (Ni (COD)_2_) to introduce Ni (0) catalytic sites, enabling the nickel to bond with the alkyne groups present in the DBA structure. Inductively coupled plasma atomic emission spectroscopy (ICP‐AES) verified a 5 wt.% nickel incorporation, suggesting that around 45% of the DBA units underwent metalation. The BET surface area reduced from 766 m^2^/g (DBA‐TMT‐POP) to 292 m^2^/g (Ni‐DBA‐TMT‐POP), indicating successful metal incorporation. After metalation, the pore size slightly decreased to 1.6 nm. Ni‐DBA‐TMT‐POP has been specifically engineered for heterogeneous catalysis, especially in desulfurization reactions. Its porous configuration, metal‐active sites, and potential for reuse make it a promising candidate for other applications. The Ni (0) active sites are evenly spread throughout Ni‐DBA‐TMT‐POP, improving the efficiency of C‐S bond cleavage for desulfurization and aiding in the extraction of sulfur from dibenzothiophenes (DBTs) and benzothiophenes (BTs), which are notable sulfur contaminants in crude oil and diesel. Depending on the sulfur compound, the desulfurization efficiency varies from 55% to 91%. Additionally, minimal nickel leaching (approximately 0.02%) reinforces the catalyst's long‐term usability over five cycles. The metalation of DBA‐TMT‐POP with nickel yields a highly stable, reusable, and effective heterogeneous catalyst for fuel desulfurization [218].

Another research team explored the catalytic applications and post‐metalation of a vanadium‐metalated POP known as VO@TPA‐POP. They chose TPA‐POP, which they synthesized by combining triphenylamine (TPA) and 1,3‐bis(4‐methoxyphenyl)‐1,3‐propanedione (MPD) through a Friedel‐Crafts alkylation reaction. The resulting structure features acetylacetonate (ACAC) groups that act as anchoring sites for the incorporation of oxo‐vanadium (V═O) into the pre‐synthesized TPA‐POP polymer. This incorporation utilized bis(2,4‐pentanedionato)‐vanadium (IV) oxide as the metal precursor, resulting in the formation of VO@TPA‐POP. In this process, vanadium is introduced as a V(IV) species, which generate VO(O)_4_ active sites. The strong coordination of these sites helps to prevent metal leaching, providing long‐term stability. Following the addition of vanadium, a slight reduction in porosity was noted, with specific surface areas changing from 354 to 298 m^2^/g, which confirms successful incorporation. They created a highly stable and recyclable heterogeneous catalyst for the selective oxidation of thioanisole (TA) to sulfoxide (MPS), achieving a 96% conversion rate with 99.5% selectivity toward sulfoxide under mild conditions. Furthermore, the catalyst effectively detoxified sulfur mustard simulants (HDs) by facilitating their oxidation into less toxic sulfoxide derivatives, offering a safer method for the decontamination of chemical warfare agents. The catalyst showed remarkable recyclability, remaining functional for up to four cycles with minimal vanadium leaching. In summary, the post‐synthetic metalation of the POP with oxo‐vanadium (V═O) successfully introduced VO(O)_4_ active sites, allowing for efficient, recyclable, and stable catalytic oxidation of both thioanisole and sulfur mustard simulants under mild conditions [219].

A Zn@BP‐POP composite from a poly‐triphenyl amine‐based POP was prepared using the post‐metalation method, reported by Boruah et al. They utilized [2,2′‐Bipyridine]‐5,5′‐dicarbonyl dichloride (BPDC) along with the redox‐active phenylamine‐derived monomer, N,N,N′,N′‐Tetrakis(4‐aminophenyl)‐1,4‐phenylenediamine (TBD), via a one‐pot Friedel–Crafts acylation process. This approach enabled the formation of C−C and C−O bonds, resulting in BP‐POP. This expanded hyper‐crosslinked conjugated framework demonstrated a CO_2_ adsorption capacity of 12.2 cm^3^/g and a 412 m^2^/g porosity. BP‐POP was later subjected to treatment with zinc acetate [Zn(acac)_2_]. to introduce Zn (II) sites into the bipyridine structures of the polymer, leading to the formation of Zn@BP‐POP. The coordination of Zn (II) enhances interactions between Lewis's acids and bases, thereby increasing the affinity and activation for CO_2_. Consequently, the CO_2_ adsorption capacity increased to 16.1 cm^3^/g, although the porosity slightly reduced to 319 m^2^/g. The composite exhibited remarkable photocatalytic capabilities, achieving a CO production rate of 215 µmol/g CO under visible‐light irradiation over 5 h, with a selectivity of 94%, thus effectively minimizing hydrogen evolution reactions (HERs). Moreover, the presence of Zn (II) sites improves charge transfer and photostability by facilitating electron mobility and hindering recombination. In summary, Zn@BP‐POP exemplifies an advanced category of metallated POPs, showcasing high selectivity, effectiveness, and durability for solar‐driven CO_2_ reduction. Coordinating single‐site Zn (II) significantly enhances catalytic activity and charge transfer, making it a promising candidate for sustainable energy and environmental applications [181].

**FIGURE 14:**
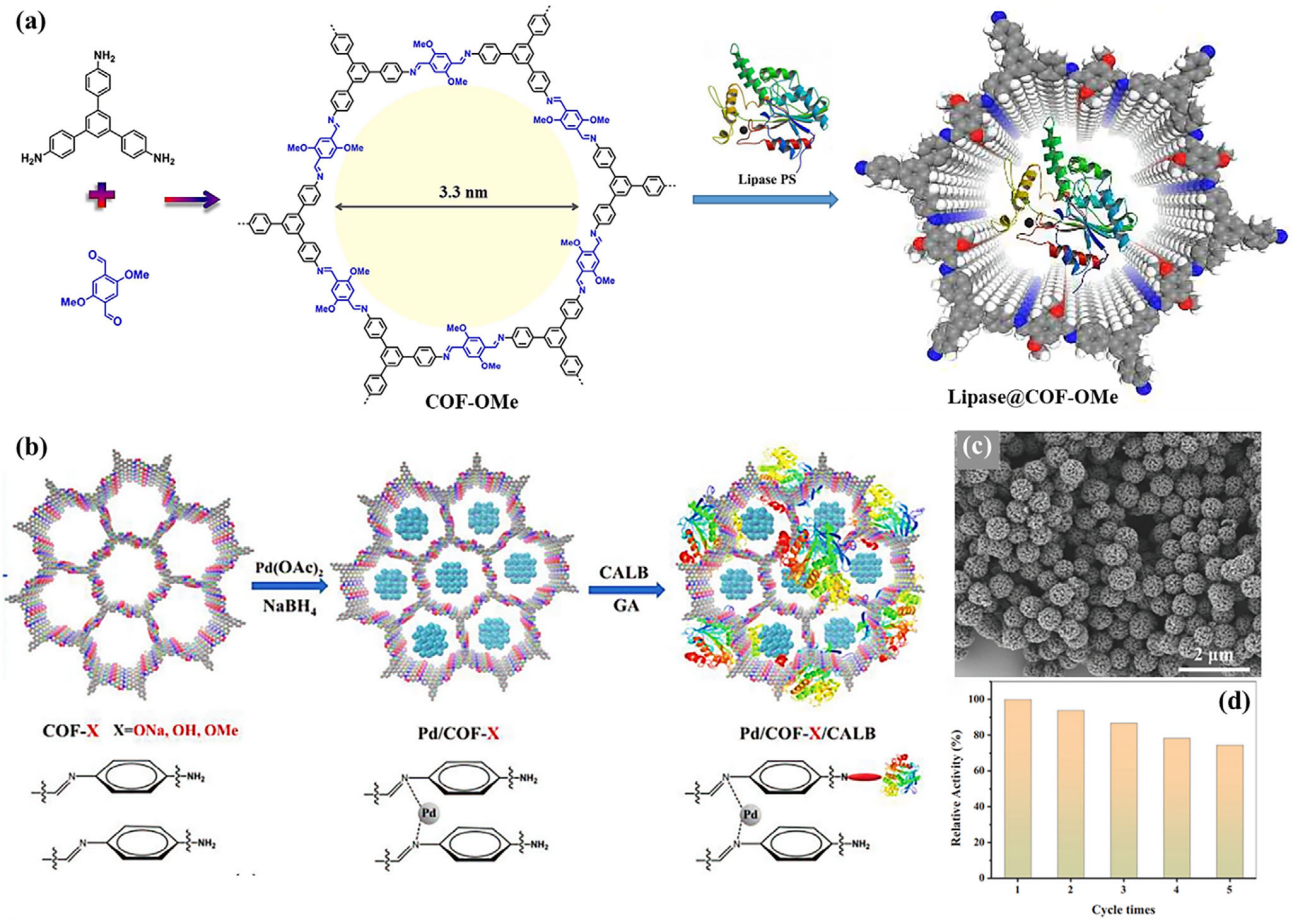
(a) Synthesis of COF‐OMe and Lipase@COF‐OMe. Reproduced with permission from ref. [220]. Copyright 2018, Journal of the American Chemical Society. (b) Preparation of Pd/COF‐X/CALB. (c) SEM images of Pd/HCOFOMe/CALB. (d) Recyclability of Pd/HCOF‐OMe/CALB for DKR. Reproduced with permission from ref. [221]. Copyright 2024, Wiley‐VCH.

The unique design of the through channel and pore structure enables a seamless flow of guest molecules between the inner and outer surfaces of the shell. This clever feature significantly boosts mass transfer efficiency, making the process more effective [220, 221]. Sun et al. investigated the use of COFs for immobilizing enzymes. They initially created COF‐OMe (TPB‐DMTP‐COF) through a Schiff‐base condensation reaction involving 1,3,5‐tris‐(4‐aminophenyl) benzene (TPB) and 2,5‐dimethoxyterephthalaldehyde (DMTP) under solvothermal conditions. The methoxy (‐OMe) groups are left accessible on the inner pore surfaces, resulting in a more hydrophobic and nonpolar atmosphere within the COF. According to the researchers' goals, COF‐OMe exhibited enhanced enzyme loading and stability because of the hydrophobic surroundings, which stabilize lipase PS, an enzyme optimized for nonpolar conditions. This arrangement encourages interfacial activation, aiding in the preservation of the enzyme's catalytically active structure. Consequently, COF‐OMe demonstrates improved enzyme activity retention in organic solvents like hexane compared to hydrophilic COFs. Lipase PS was prepared in a phosphate buffer solution to create the enzyme‐COF composite. The enzyme solution was then mixed with COF‐OMe and stirred at room temperature for 6 h to facilitate the enzyme's diffusion into the COF's pores (Figure 14a). The enzyme molecules adhere to the COF surface and penetrate the pore network through hydrophobic interactions and hydrogen bonding. The BET surface area reduced from 1740 to 784 m^2^/g due to the presence of the enzyme, while the pore size remained constant at 3.3 nm but was partially obstructed by the enzyme molecules. The pore volume decreased from 1.62 to 0.59 cm^3^/g because the pores were filled by the enzyme. The resulting Lipase@COF‐OMe composite maintains more than 90% of its activity after six cycles, indicating outstanding recyclability. This composite merges the high stability and porosity of COF‐OMe with the catalytic performance of lipase PS, making it ideal for biocatalysis, pharmaceutical applications, and industrial processes [220].

In similar work, Hollow COF‐OMe (HCOF‐OMe) was examined by Zhao et al. The team synthesized HCOF‐OMe through a template‐free controlled precipitation technique involving TAPB and DMTA. This method led to the creation of hollow spherical COF structures featuring channels that enhance substrate diffusion and catalytic performance. The researchers subsequently filled the pores and cavities of HCOF‐OMe with Pd nanoparticles (Pd NPs) and immobilized Candida antarctica lipase B (CALB) on the exterior surface, yielding a catalyst referred to as Pd/HCOF‐OMe/CALB (Figure 14b,c). Some of the pores are occupied by Pd NPs, which slightly limits the available space. Additionally, the immobilization of CALB obstructs both internal and external pores, further reducing the accessible pore size. The immobilization of CALB results in the most substantial decrease in the BET surface area, dropping from 1864 to 705 m^2^/g, as the enzyme molecules obstruct several pores, hindering accessibility. Pd NPs act as catalytic sites for the racemization of amines. The research demonstrated that the addition of CALB achieved a 94% yield in the kinetic resolution (DKR) of primary amines, highlighting excellent catalytic efficiency. Furthermore, the catalyst exhibited sustained high activity across five cycles, demonstrating remarkable reusability and stability (Figure 14d). This study provides a framework for the design of highly active metal‐enzyme integrated catalysts (MEICs) by merging molecular engineering and morphology control in COFs [221].

#### 4.2.3 Surface Modification

Through post‐synthetic modification, the surface of POPs can be functionalized with enzymes, Apt, and other biological molecules. This functionalization enhances the interaction between POPs and target biomolecules, enabling biosensing, drug delivery, and biocatalysis applications. The high surface area and tunable chemistry of POPs provide a suitable environment to retain the activity and stability of the immobilized species [222, 223, 224, 225].

**FIGURE 15:**
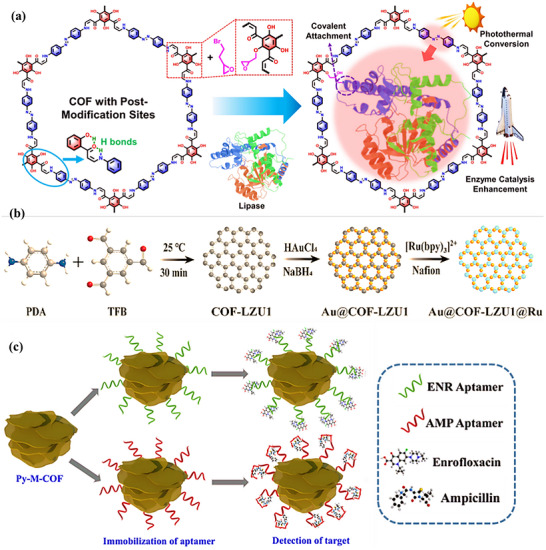
(a) The enzyme is immobilized on the COF's surface, which exhibits photothermal properties. Reproduced with permission from ref. [226]. Copyright 2025, Wiley‐VCH. (b) The process for synthesizing luminescent nanomaterials composed of Au@COF‐LZU1@Ru. Reproduced with permission from ref. [227]. Copyright 2024, Elsevier. (c) Illustration of the electrochemical detection method for ENR and AMP employing aptasensors based on Py‐M‐COF. Reproduced with permission from ref. [228]. Copyright 2019, Elsevier.

Hao et al. successfully created NKCOF‐73 using an intramolecular hydrogen bond (IMHB) activated Michael addition‐elimination reaction. The synthesis process consisted of two main components, TDOEP (2Z,2′Z,2″Z)‐1,1′,1″‐(2,4,6‐trihydroxybenzene‐1,3,5‐triyl)tris(3‐(dimethylamino)prop‐2‐en‐1‐one) and ADDA (E)‐4,4′‐(diazene‐1,2‐diyl)dianiline, which already contained azo (‐N = N‐) groups in their structures. These azo groups were intentionally included in the COF to provide a photothermal effect, allowing the COF to absorb solar energy and generate localized heat. The structure of the COF exhibited high crystallinity, desirable mesoporous properties, and a high density of hydroxyl functional groups, making it a promising candidate for enzyme immobilization (Figure 15a). Lipase was covalently bound to NKCOF‐73 through post‐modification with epibromohydrin, which introduced reactive glycidyl groups for enzyme attachment. This post‐modification notably enhanced enzyme loading and catalytic efficiency. The presence of the azo group enabled the stable attachment of lipase while benefiting from the existing azo groups that boosted enzyme activity through localized heating. This covalent immobilization strategy provided improved stability, minimized enzyme leaching, and enhanced reusability compared to conventional physical adsorption methods. After several reaction cycles, the immobilized enzyme displayed increased stability and sustained high catalytic activity. It achieved nearly theoretical conversion rates (around 50%) for various racemic substrates, surpassing the performance of free enzymes. Initially, the BET surface area of NKCOF‐73 was 1271 m^2^/g, which slightly decreased to 1203 m^2^/g following post‐modification. The pore size of NKCOF‐73 was initially 3.3 nm, reducing to 3.2 nm after modification. These minor reductions suggest that the modification process did not significantly impact the COF's porous structure, enabling effective enzyme immobilization and retention of catalytic activity [226].

In a separate investigation, COF‐LZU1 was created through a Schiff‐base condensation reaction involving 1,3,5‐triformylbenzene (TFB) and PPD under solvothermal conditions. This material was employed as a novel electrochemiluminescent (ECL) cytosensor to capture and identify rare circulating tumor cells (CTCs). To promote the attachment of biomolecules, COF‐LZU1 underwent modification by adding functional groups that enhance aptamer binding. This process involved the in situ deposition of gold nanoparticles (AuNPs) onto COF‐LZU1 to boost conductivity and provide sites that react with thiol groups (Figure 15b). The surface of COF‐AuNP allows for the binding of biomolecules through thiol (‐SH) modifications via Au–S bonds. Moreover, a Nafion solution was applied as a cross‐linking agent to impart a negative charge to the COF surface, thereby improving electrostatic interactions with positively charged biomolecules. The design for immobilizing the aptamer on COF‐LZU1 was meticulously structured to guarantee robust and stable attachment, facilitating the efficient capture and detection of CTCs. This approach integrates biotin‐streptavidin chemistry, covalent bonding methods, and electrostatic interactions. Two (Apt1 and Apt2) were specifically designed to target the EpCAM and MUC1 biomarkers present on CTCs. The biotin‐streptavidin linkage ensures the aptamer's strong, irreversible attachment, while gold‐thiol (Au–S) bonds and electrostatic interactions further contribute to stability. COF‐LZU1 boasts a high BET surface area, indicating a porous structure well‐suited for immobilizing enzymes or Apt. The pore dimension is roughly 1.8 nm, which aids in efficiently loading biomolecules. Although a minor decrease in the BET surface area was noted due to the binding of Apt, the pore size remained largely unaltered, ensuring effective diffusion of biomolecules. This technique provides high sensitivity, specificity, and clinical relevance, making it an effective tool for cancer diagnostics [227]. Functionalized COFs have attracted considerable interest in sensor applications because of their adjustable structures and high porosity. In addition to their role as fluorescent sensors modified with DNA, COFs have also been investigated for electrochemical sensing. For example, Wang et al. described the creation of a π‐conjugated, mesoporous COF (Py‐M‐COF) featuring a pore size of 3.9 nm. This material was synthesized through a condensation polymerization reaction between 1,3,6,8‐tetrakis(4‐formylphenyl) pyrene and melamine, resulting in imine linkages at 150°C over 48 h. To improve the selectivity of electrochemical sensors based on COFs, the authors modified the surface of Py‐M‐COF with Apt synthetic single‐stranded DNA or RNA molecules that specifically bind to targets through unique three‐dimensional shapes (Figure 15c). Apt provide benefits such as high specificity, stability, and ease of synthesis, which make them particularly effective for bioassay applications. In this research, the scientists developed an aptamer‐functionalized Py‐M‐COF sensor (aptasensor) to detect commonly used antibiotics like enrofloxacin (ENR) and ampicillin (AMP) [228].

The creation of organic porous structures paired with inorganic nanocomposites has garnered considerable interest because of the high surface area offered by POPs, which act as efficient reaction sites for the integration of inorganic materials. Once the desired POP is synthesized, its surface is subsequently modified with inorganic substances. The addition of inorganic nanocomposites is essential for preventing the aggregation of nanoparticles while also enhancing the synergistic interactions among the individual components, ultimately boosting their overall functionality [229, 230, 231].

**FIGURE 16:**
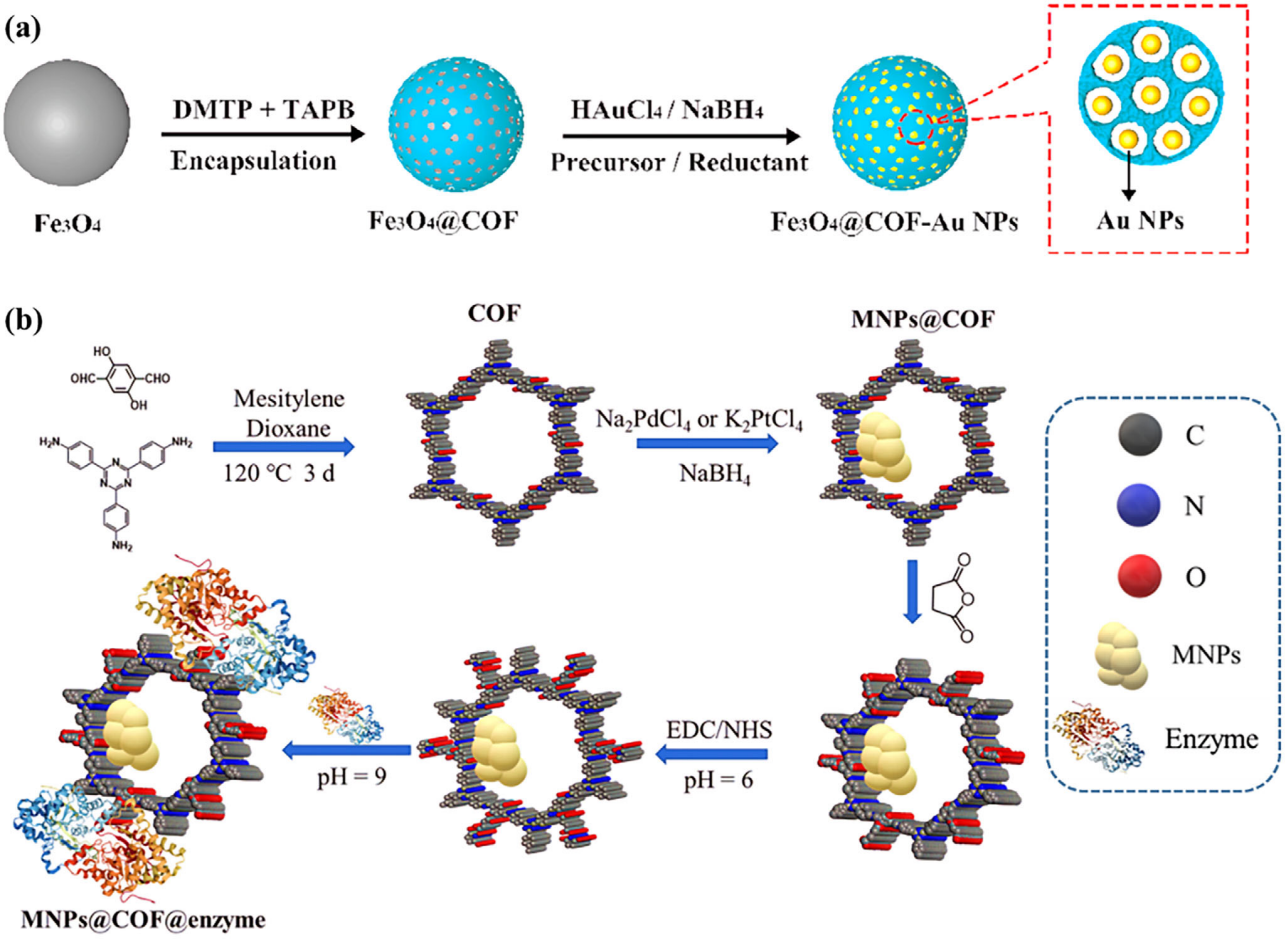
(a) Preparation method for Fe_3_O_4_@COF–Au NPs. Reproduced with permission from ref. [232]. Copyright 2022, Elsevier. (b) Preparation method of MNPs@COF@enzyme. Reproduced with permission from ref. [233]. Copyright 2020, American Chemical Society.

Li et al. presented a porous Fe_3_O_4_@COF immobilized with (Fe_3_O_4_@COF–Au NPs) as a functional nanocarrier for the sensitive electrochemical detection of ATP. The Fe_3_O_4_@COF nanocomposite was created through a hydrothermal method that employs a Schiff base reaction involving 1,3,5‐tris(4‐aminophenyl) benzene (TAPB) and 2,5‐dimethoxyterephthaldehyde (DMTP), along with Fe_3_O_4_ nanoparticles. This composite serves as a platform for immobilizing (Au NPs), enhancing their catalytic effectiveness and reducing aggregation likelihood (Figure 16a). The porous nature of the COF encapsulates some Au NPs within its nanochannels while anchoring other particles on the outer surface of the COF shell. This dual arrangement increases catalytic efficiency and fosters a stable microenvironment for electrochemical applications. A post‐reduction process utilizing NaBH_4_ was applied to attach ultrafine Au NPs to the COF surface firmly. The hydrophobic, porous framework of the COF delivers a steady microenvironment, improving the dispersibility of the Au NPs. Strong interactions between the Au NPs and the COF surface, facilitated by coordination bonds and electrostatic forces, further enhanced their stability. The Fe_3_O_4_@COF–Au NPs composite showed increased charge transfer efficiency, making it ideal for electrochemical sensing. Although the COF's porosity was preserved, incorporating the Au NPs into the pores probably caused a slight decrease in pore size due to partial occupation of the channels. The system employs a branched hybridization chain reaction (b‐HCR) to detect high‐sensitivity ATP, achieving an extensive linear range from 5 pm to 50 µm and a low detection limit of 1.6 pM. The COF's functionalization with Fe_3_O_4_ and Au NPs effectively improved its catalytic, magnetic, and electrochemical characteristics, establishing a stable and efficient ATP detection platform with high sensitivity and selectivity [232].

A nanocatalyst was created by modifying a COF with metal nanoparticles (MNPs) and enzymes (Figure 16b). The COF, designated as Tz‐Da, was produced through a Schiff base reaction involving 2,4,6‐tris(4‐aminophenyl)‐1,3,5‐triazine (Tz) and 2,5‐dihydroxy‐1,4‐benzenedicarboxaldehyde (Da) under solvothermal conditions. First, the Tz‐Da was altered using succinic anhydride (SA) to add carboxyl groups. These carboxyl groups were then activated with EDC (1‐ethyl‐3‐(3‐dimethylaminopropyl) carbodiimide) and NHS (N‐hydroxysuccinimide), allowing for the covalent attachment of the enzyme organophosphorus hydrolase (OPH) to the surface of the COF. Palladium nanoparticles (Pd NPs) were incorporated into the pores of the COF (Tz‐Da), and OPH was covalently bound to the surface. This approach of spatially separating the co‐immobilization improved both catalytic efficiency and stability, supporting the cascade degradation of organophosphate nerve agents. The researchers also explored the adaptability of this method by using different COFs and metals, such as platinum nanoparticles (Pt NPs), along with glucose oxidase (GOx), for applications including phenol detection. In comparison to free enzymes, the immobilized enzyme demonstrated higher resistance to thermal changes and pH fluctuations. The unmodified COF (Tz‐Da) had a surface area of 2125 m^2^/g. After the incorporation of Pd NPs (Pd@Tz‐Da), the BET surface area reduced to 920 m^2^/g, indicating occupation of the internal pores by the Pd NPs. Following the immobilization of OPH (Pd@Tz‐Da@OPH), the surface area decreased further to 549 m^2^/g as the enzyme covered part of the surface area and blocked more pores. The original COF had a pore size of 3.05 nm. After loading with Pd, the pore volume was diminished as the pores became partially filled with the Pd NPs. The pore size reduced additionally after enzyme immobilization, confirming the successful binding of OPH to the COF surface. The separation of MNPs and enzymes effectively safeguarded against enzyme inactivation caused by metal toxicity. The functionalized COF preserved its catalytic function over several cycles. This strategy was effectively broadened to different COFs and catalytic systems, showing extensive applicability [233].

### 4.3 Amine Functionalization of POP for Enhanced Performance

The functionalization of POPs with amines is an effective method to enhance their capabilities in CO_2_ capture, catalysis [28, 234, 235, 236]. This can be accomplished through four primary methods, directly synthesizing amine‐functionalized monomers [237, 238, 239], grafting amine groups to the polymer backbone [240, 241, 242], incorporating amines into the porous structure [243, 244, 245]. These modifications add nitrogen‐rich active sites, improving the material's affinity, selectivity, and reactivity. Consequently, amine‐functionalized POPs exhibit superior performance, especially in selective gas separation and environmental cleanup.

#### 4.3.1 Direct Synthesis

The method of directly synthesizing amine groups into POPs involves the incorporation of amine‐containing monomers or linkers during the polymerization process. This approach ensures that the amine groups are uniformly distributed throughout the material and are securely attached, resulting in high stability and accessibility. This technique is especially effective for capturing CO_2_, as the integrated amine groups interact strongly with CO_2_ molecules through acid‐base interactions or carbamate formation [240, 246, 247]. As a result, POPs produced with this method typically exhibit high capacities for CO_2_ uptake, good selectivity, and excellent recyclability in practical applications.

Li et al. synthesized a Reference COF using TFPT (2,4,6‐tris(4‐formylphenyl)‐1,3,5‐triazine) and PPD, a simple and symmetrical diamine. The linkages formed in this framework are imine linkages (─C═N─), which arise from a Schiff base reaction between the aldehyde and the amine. The RefCOF does not contain free amino groups and exhibits poor stability in acidic conditions. It demonstrated a CO_2_ uptake of 30.73 cm^3^ g^−1^ and achieved a uranium removal efficiency of 73.3%. The researchers then directly amino‐functionalized the RefCOF using Bandrowski's base (BB) through a novel method incorporating a one‐step regioselective cyclization and aromatization strategy. The synthesis of BB involves using 1,4‐phenylenediamine (PDA) as a precursor, which undergoes controlled oxidative coupling to form a molecule containing two types of amino groups, highly reactive (cyclohexadienyl‐linked) and low‐reactivity (phenyl‐linked). The resulting COF features benzimidazole linkages and free amino groups, which are rare to preserve during direct COF synthesis. High‐activity amino groups react with aldehydes to form imine linkages, which subsequently cyclize into benzimidazole rings that stabilize the framework. Meanwhile, low‐activity amino groups on the phenyl ring remain unreacted and accessible for subsequent functionalization. BBCOF is stable in strong acids and bases, with an improved CO_2_ uptake of 43.83 cm^3^ g^−1^, achieving a uranium removal efficiency of 96.6%. Its BET surface area is increased to 364.4 m^2^/g. This direct functionalization strategy enables the synthesis of COFs with built‐in functional groups, offering superior chemical and thermal stability along with enhanced CO_2_ capture capacity. BBCOF outperforms traditional imine‐based COFs like RefCOF in nearly all performance metrics, demonstrating the effectiveness of this regioselective approach [248].

In another study, a COF featuring aromatic amine functionalities was created to improve CO_2_/N_2_ gas separation while maintaining its crystallinity and porosity. The synthesis of Me_3_TFB‐BD COF was achieved using Me_3_TFB (2,4,6‐trimethylbenzene‐1,3,5‐tricarbaldehyde), a trialdehyde, and BD, a diamine, through the imine condensation reaction of the aldehyde (–CHO) and amine (–NH_2_) groups. After this, a dynamic linker exchange was conducted, substituting BD with (NH_2_) _2_BD via a dynamic imine bond exchange, resulting in the formation of Me_3_TFB‐(NH_2_) _2_BD. This approach successfully preserved high crystallinity and generated a substantial surface area of 1624 ± 89 m^2^/g, while the original COF had a BET surface area of approximately 1600 m^2^/g. Following functionalization, the capacity for CO_2_ adsorption increased by around 47%–77%. The CO_2_ capacity at 273 K and 1 bar grew from 0.76 to 1.12 mmol/g. Moreover, the CO_2_/N_2_ selectivity improved more than six times at both temperatures, a significant enhancement that underscores the COF's impressive performance. The interactions are defined by physisorption rather than chemisorption, facilitating low‐energy regeneration. This research showcases an effective method for direct COF functionalization through dynamic linker exchange, enabling the introduction of aromatic amine groups. These functional groups significantly enhance CO_2_ adsorption and selectivity while preserving excellent crystallinity and porosity. In contrast to aliphatic amines that bind CO_2_ through chemisorption, this COF interacts via physisorption, making it energy‐efficient and suitable for recycling in carbon capture applications [249].

Fayemiwo and colleagues investigated a series of amine‐functionalized HCPs, known as HCP‐MAAMs, to evaluate their effectiveness in capturing CO_2_ at low pressures. This research is particularly relevant for applications such as post‐combustion gas streams from coal‐fired power plants. The polymers were synthesized using a free‐radical copolymerization process that involved methacrylamide (MAAM) and ethylene glycol dimethacrylate (EGDMA), with azobisisobutyronitrile (AIBN) serving as the initiator. The polymers incorporate amine groups directly from the MAAM component and were prepared with varying molar ratios (S) of MAAM to EGDMA ranging from 0.3 to 0.9. These materials exhibited strong CO_2_ binding capabilities under low partial pressures (0.02–0.15 bar), improving CO_2_/N_2_ selectivity. The calculated isosteric heat of adsorption (Q_st_) values were as high as 35 kJ/mol, indicating that CO_2_ interacts with the amine groups through electrostatic forces. However, increasing the MAAM content reduced the surface area, decreasing CO_2_ uptake. Notably, the sample with a ratio of S = 0.3 demonstrated the highest CO_2_ adsorption capacity, reaching 1.56 mmol/g at 273 K. Further enhancements may be possible by using alternative amine‐rich crosslinkers, such as N, N′‐methylene bis (acrylamide). The recyclability of the HCP with S = 0.6 was assessed over five adsorption–desorption cycles, conducted under 0.15 bar CO_2_ at 298 K for adsorption and 393 K with N_2_ for desorption. The material maintained most of its capacity, showing only a 1.9% decrease in CO_2_ uptake over the five cycles. Nevertheless, additional research is needed to evaluate these materials' long‐term stability and regeneration performance thoroughly [238].

#### 4.3.2 Grafting

Attaching amine groups to the backbone of polymers from the POP categories enhances their functionality and selectivity. This process generally involves the reaction of amine‐containing compounds with the reactive sites of the POP, resulting in the formation of stable covalent bonds [250, 251, 252, 253]. Such modifications increase the material's affinity for particular molecules, rendering it suitable for applications in gas separation, catalysis, and adsorption [28, 254, 255, 256, 257]. Amine‐functionalized POPs display heightened surface polarity and improved interactions with target molecules [258]. The adjustable nature of amine grafting enables the optimization of POPs for a range of industrial and environmental applications.

**FIGURE 17:**
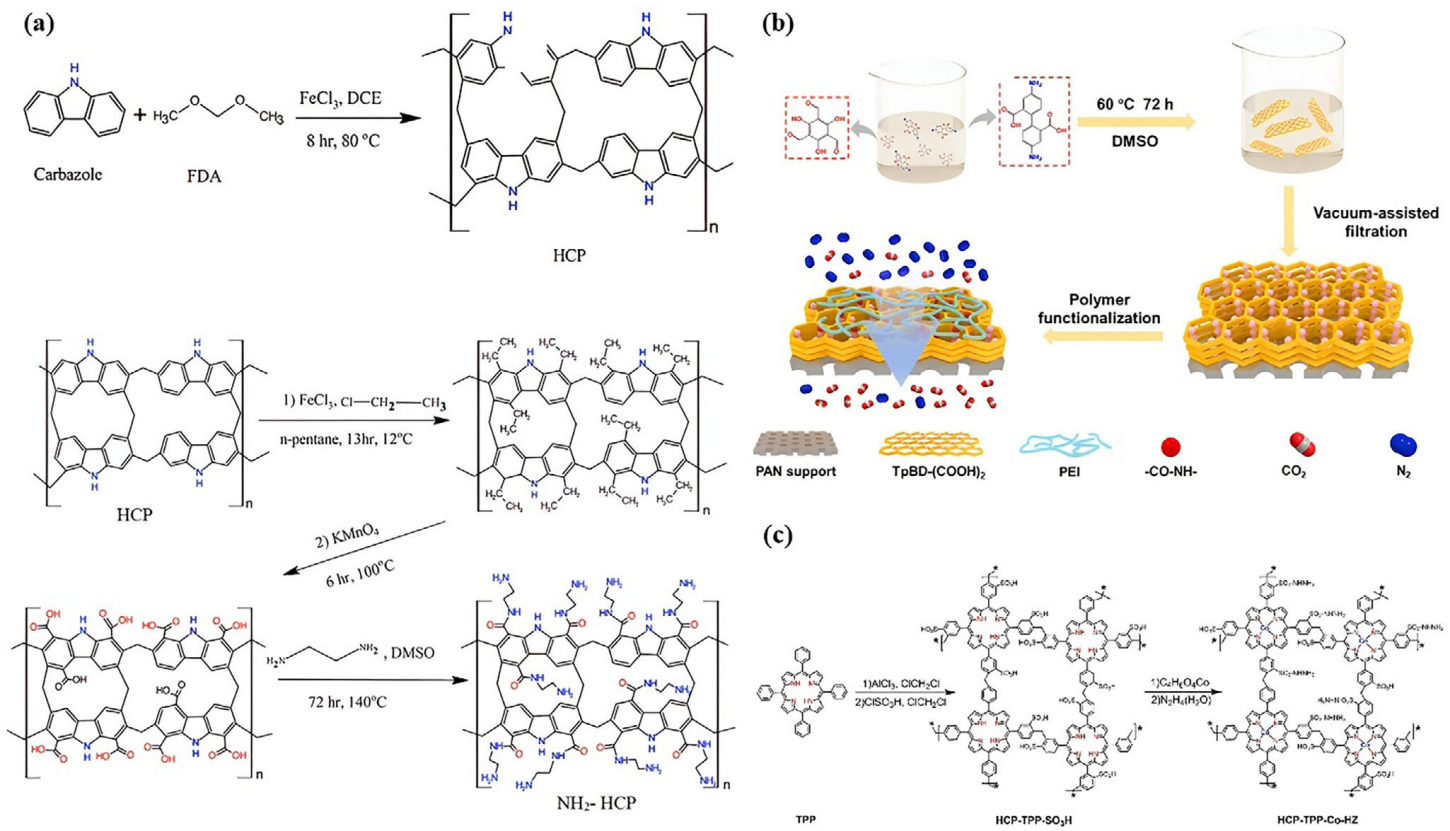
(a) HCP adsorbent and NH_2_‐HCP adsorbent synthesis scheme. Reproduced with permission from ref. [250]. Copyright 2023, Elsevier. (b) Preparation of the cCOF‐PEI membrane for fabrication. Reproduced with permission from ref. [254]. Copyright 2024, Elsevier. (c) Synthesis of HCP‐TPP‐Co‐HZ. Reproduced with permission from ref. [252]. Copyright 2022, Elsevier.

A new approach to improve CO_2_/N_2_ gas separation membranes involves altering ultrathin COF membranes, as outlined by Zeng et al. They create cCOF nanosheets through a Schiff base condensation reaction involving an amine‐containing monomer, 4,4′‐diamino‐[1,1′‐biphenyl]‐2,2′‐dicarboxylic acid (BD‐(COOH) _2_), and an aldehyde monomer, 1,3,5‐triformylphloroglucinol (Tp). This process forms a β‐ketamine‐linked COF that incorporates carboxyl functional groups within its structure. The COF is subsequently functionalized with polyethyleneimine (PEI), a polymer‐rich in amine groups, employing EDC/NHS coupling chemistry to bond it covalently to the carboxyl groups on the COF (Figure 17b). The resulting carboxylated (cCOF) nanosheets showcased a BET surface area of 812 m^2^/g and an average pore size of 2.02 nm before modification. These characteristics highlight the highly porous and crystalline nature of the β‐ketamine‐linked COF structure. Following the functionalization with PEI, the surface area and pore size significantly decreased due to partial pore obstruction and dense grafting of PEI chains. Specifically, the BET surface area fell to 315 m^2^/g, and the pore size was reduced to 1.54 nm. This decrease benefits selective gas separation, as the narrowed pore channels and adding amine groups enhance the membrane's affinity for CO_2_. Regarding CO_2_ capture efficiency, the unmodified cCOF membrane demonstrated an impressive CO_2_ permeance of approximately 5500 GPU. Still, it displayed a low CO_2_/N_2_ selectivity of merely 0.96, indicating non‐selective diffusion caused by the larger pore size. Upon PEI functionalization, especially in the optimized membrane (cCOF‐PEI‐1), CO_2_ permeance decreased to 1004 GPU, while CO_2_/N_2_ selectivity rose significantly to 33.7. This notable improvement is due to the facilitated transport mechanism, where the abundant amine groups from PEI temporarily interact with CO_2_, enhancing its selective permeation. These figures not only meet but surpass the benchmarks for industrial post‐combustion CO_2_ capture (i.e., CO_2_ permeance >1000 GPU and selectivity >20), showcasing the effectiveness of the PEI‐functionalized COF membranes [254].

Torkashvand et al. synthesized an HCP using carbazole monomers via a Friedel–Crafts alkylation process, utilizing the functional groups of the monomers, which include aromatic rings, C–H, and N–H bonds. To improve the polymer's adsorption properties, they employed a three‐step amine grafting technique that resulted in NH_2_‐HCP. The initial step was alkylation, during which ethyl groups were added using chloroethane along with FeCl_3_. In the second stage, these ethyl groups were oxidized to form carboxylic acid groups using potassium permanganate (KMnO_4_). Finally, in the amidation phase, the resulting carboxyl groups were treated with ethylenediamine (EDA) in DMSO under reflux conditions, effectively embedding primary and secondary amine functionalities into the polymer structure (Figure 17a). This modification altered the internal architecture of the polymer by transforming passive physical pores into chemically active adsorption sites enriched with amines derived from EDA. BET surface analysis indicated a significant decrease in surface area from 705.35 m^2^/g (HCP) to 471.21 m^2^/g (NH_2_‐HCP), suggesting that the amine groups took up space in the porous structure. Likewise, the micropore volume decreased from 0.161 to 0.117 cm^3^/g, and the micropore area diminished from 167.91 to 109.11 m^2^/g. The total pore volume fell from 0.954 to 0.517 cm^3^/g, and the average pore width slightly reduced from 5.212 to 4.463 nm. Despite the decline in physical surface characteristics, the CO_2_ adsorption capacity significantly increased due to the chemical affinity provided by the amine groups. At 298 K and 5 bar, the unmodified HCP could adsorb 182.13 mg/g of CO_2_, while the amine‐grafted NH_2_‐HCP reached 236.37 mg/g, representing a ∼30% improvement. This enhancement was linked to the surface area and the establishment of strong chemical interactions between CO_2_ and the amine sites, including carbamate and ammonium species. The transition from entirely physical adsorption to a mix of physisorption and chemisorption highlights the effectiveness of amine grafting in enhancing CO_2_ capture under practical conditions [250].

Another study examines the development of chemisorption sites to improve CO_2_ chemical conversion activity through the amine modification of metalloporphyrin‐based HCPs. The synthesis of HCP‐TPP starts with the dispersion of tetraphenyl porphyrin (TPP) in dichloromethane (CH_2_Cl_2_) within a nitrogen environment. The HCP‐TPP is subsequently treated with chlorosulfonic acid (Cl‐SO_3_H) to add sulfonic groups, resulting in HCP‐TPP‐SO_3_H. After that, cobalt acetate is combined with the sulfonated HCP‐TPP to form a cobalt‐functionalized porous polymer known as HCP‐TPP‐Co‐SO_3_H (Figure 17c). For the amine grafting process, hydrazine hydrate (HZ) is reacted with HCP‐TPP‐Co‐SO_3_H. The nucleophilic attack of the ‐NH_2_ group present in HZ on the electrophilic sulfur atom of the ‐SO_3_H results in forming C‐N bonds, converting ‐SO_3_H into ‐SO_2_‐NH‐NH_2_. Following the amine grafting, the surface area is substantially decreased from 1314 to 189 m^2^/g, likely due to pore obstruction caused by the large amine groups. The pore volume also declines from 1.29 to 0.22 cm^3^/g. Despite the reduction in surface area, CO_2_ uptake remains relatively high due to the increased chemisorption affinity brought about by the amine groups. The isosteric heat of adsorption (Q_st_) rises from 28.3 to 34.7 kJ/mol, suggesting stronger interactions between CO_2_ and the modified HCP. The coordination of cobalt ions with the amine groups stabilizes the structure, facilitating effective reuse and improving catalytic performance. The catalytic efficiency for converting CO_2_ to cyclic carbonates shows a significant enhancement after grafting, achieving yields of up to 98% compared to 64% before grafting. The structural changes resulting from amine grafting mainly involve the introduction of amino groups, an increase in nitrogen content, a reduction in pore size, and a slight decrease in thermal stability. Despite these alterations, the enhanced chemisorption and catalytic performance render the modified HCP considerably more efficient for CO_2_ capture and conversion [252].

#### 4.3.3 Impregnation

The amine impregnation of POPs entails incorporating amine functional groups into the polymer's framework, enhancing its chemical reactivity and capacity for adsorption. This modification is generally accomplished by immersing the POP in compounds containing amines or chemically reacting it with those compounds. The amine‐functionalized POPs exhibit enhanced performance in various applications, including CO_2_ capture, catalysis, and the extraction of heavy metal ions. The amine groups act as active sites for the binding or interaction with targeted molecules [259, 260, 261].

Li et al. created a COF modified with PEI, referred to as COF‐709. The approach involves introducing and grafting amines through a stable C−S covalent bond between thiol‐modified branched polyethyleneimine (SH‐bPEI) and the COF skeleton. The synthesis of COF‐709 occurs via imine condensation between tetrafluoro‐terphenyl dicarbaldehyde (TFTDA) and tetrakis(4‐aminophenyl) pyrene (TAPPy). The resultant imine‐COF‐709 is subsequently oxidized to amide‐COF‐709 using sodium chlorite, which boosts its chemical stability for further modifications. During the PEI impregnation process, SH‐bPEI is introduced into the pores of the COF through SNAr facilitated by sodium hydride. This method forms C−S covalent bonds between the thiol groups of bPEI and the COF framework. As a result, the surface area of COF‐709 significantly declines from 1880 to 290 m^2^/g after the impregnation with PEI. The pore size decreases from 3.0 to 0.8 nm due to the substantial SH‐bPEI chains occupying the pores. Despite the reduced surface area, COF‐709 shows improved CO_2_ adsorption, especially in humid conditions, reaching a capacity of 1.24 mmol/g at 75% relative humidity. The strong chemisorption interactions between CO_2_ and PEI greatly enhance the efficiency of CO_2_ capture. Furthermore, the modified COF's hydrophobic characteristics minimize water adsorption, making the material easier to regenerate in a more energy‐efficient fashion. The covalent bonding of PEI ensures high stability and resistance to amine loss, even after undergoing 10 adsorption–desorption cycles. In summary, the post‐synthetic amine impregnation of COF‐709 markedly enhances CO_2_ capture performance by boosting the material's affinity for chemisorption. Although there is a trade‐off in the form of reduced BET surface area and pore volume, the stabilizing C−S bonds provide exceptional cyclic stability and moisture resistance [260].

In a different study, the researchers aimed to improve the CO_2_ capture efficiency of a covalent organic polymer (COP) by altering its structure to enhance amine loading. Despite being inherently microporous and selective for CO_2_ due to its nitrogen‐rich composition, COP‐1 has a limited pore volume and small pore size, which restricts effective amine incorporation. To overcome these challenges, the team employed a complex templating method using 12 nm silica nanoparticles to create a mesoporous variant of COP‐1, known as SiCOP‐1. The templating procedure involved polymerizing cyanuric chloride and piperazine in the presence of silica particles, followed by the removal of silica through chemical etching with hot sodium hydroxide (NaOH). This technique significantly enhanced the material's surface area and pore volume, particularly within the mesoporous range (∼30 nm), while maintaining its nitrogen functionality and structural stability. After synthesis, several amines, such as triethylenetetramine (TETA), pentaethylenehexamine (PEHA), and polyethylenimine PEI were physically loaded into both COP‐1 and SiCOP‐1 to create chemisorptive CO_2_‐binding sites. The investigation highlighted notable differences in the textural and functional characteristics of COP‐1 compared to the silica‐templated SiCOP‐1, particularly following amine impregnation. COP‐1 displayed a comparatively low BET surface area of 57.8 m^2^/g, a pore volume of 0.13 cm^3^/g, and an average pore size of about 4 nm. This limited porosity restricted effective amine loading, resulting in moderate CO_2_ capture capabilities of 8.12 mg/g at 298 K and 18.92 mg/g at 273 K under a 0.15 bar pressure. In contrast, SiCOP‐1, produced using silica nanoparticles as a rigid template, exhibited significantly enhanced mesoporosity. The BET surface area more than doubled to 131.8 m^2^/g, and the pore volume nearly tripled to 0.38 cm^3^/g. The pore size distribution changed notably, introducing dominant mesopores around 28–32 nm, which are conducive to amine diffusion and loading. As a result, even prior to amine addition, CO_2_ uptake increased to 12.76 mg/g at 298 K and 26.88 mg/g at 273 K. When both materials were impregnated with 100 wt.% TETA, a decrease in surface area and pore volume was observed due to pore filling. However, this compromise was offset by a substantial increase in CO_2_ capacity. The amine‐loaded SiCOP‐1 (SiCOP‐1‐TETA100) retained a high surface area of 74.1 m^2^/g and a pore volume of 0.35 cm^3^/g while achieving a CO_2_ uptake of 32.97 mg/g at 298 K and 46.18 mg/g at 273 K, representing a fourfold increase compared to the unmodified COP‐1 at room temperature. In comparison, COP‐1‐TETA100, although improved, only reached 17.61 mg/g at 298 K, indicating that mesoporosity is vital for efficient amine incorporation and enhanced CO_2_ chemisorption. Overall, the conversion from COP‐1 to SiCOP‐1 led to a material with significantly larger pores and a greater capacity for amine uptake, which directly resulted in improved CO_2_ capture performance under conditions relevant to flue gas [262].

The development of a low‐cost and efficient CO_2_ sorbent based on a nanoporous COP‐19 has led to a polymer that was further impregnated with PEI to enhance CO_2_ capture performance under realistic conditions, as reported by Patel et al.; COP‐19 was produced through a one‐pot polycondensation reaction of melamine and terephthalaldehyde in dimethyl sulfoxide at 180°C under a nitrogen atmosphere for 72 h. The outcome was a thermally stable polymer with micro‐ and mesoporous characteristics and a high surface area of 640 m^2^/g, making it well‐suited for amine loading. To enhance chemisorption, branched PEI with molecular weights of 800 and 25 000 was physically incorporated into COP‐19 at different loadings ranging from 24% to 46 wt.%. Although the unmodified COP‐19 demonstrated a high BET surface area of 640 m^2^/g and a mixed microporous‐ and mesoporous structure that allowed significant CO_2_ physisorption, increasing the amount of PEI resulted in a marked decrease in BET surface area due to pore blockage caused by the larger polymer chains. Nevertheless, despite this reduction in surface area, the CO_2_ capture performance significantly improved after amine impregnation, particularly at lower pressures. The modified COP‐97B, with a 36.4% PEI loading, achieved a CO_2_ uptake of 69 mg/g at 298 K and 0.15 bar, nearly four times greater than the unmodified COP‐19 under identical conditions. Notably, the study showed that CO_2_/N_2_ selectivity increased significantly post‐amine loading, reaching values as high as 2511 at 298 K for COP‐97C. This underscores the importance of amine functionalization in boosting CO_2_ capture performance, more so than simply optimizing surface area. Additionally, under conditions with humid gas, the CO_2_ uptake was even more pronounced, reaching up to 100 mg/g at 313 K, which was attributed to the formation of bicarbonate in the presence of water vapor [263].

## 5 Applications of POPs

### 5.1 CO_2_ Capture and Storage/ Separation of Complex Gas Mixtures

#### 5.1.1 CO_2_ Capture

POPs' structural tunability and functional variety have made them stand out as materials in CO_2_ adsorption research [264, 265]. Recent developments in COFs [266, 267, 268, 269, 270, 271, 272], HCPs [273, 274, 275], and CMPs [276, 277] have all made significant contributions to increasing CO_2_ capture efficiency. By analyzing these materials, trends and tactics that improve CO_2_ adsorption capacity, a crucial component for energy and environmental applications, can be found.

In research by Yin et al. [271], the amino‐functionalized imidazole ionic liquid‐modified COFs ([AeImBr]_X%_‐TAPT‐COFs) had the greatest CO_2_ adsorption capacity among the several COFs investigated, reaching 2.67 mmol/g at 273 K and 1 bar. The addition of amino groups and imidazole cations has been credited with increasing the CO_2_ affinity by promoting catalytic CO_2_ conversion in addition to increasing adsorption. After eight cycles, this material produced a 95% yield of cyclochloroallyl carbonate, demonstrating its stability and versatility. In contrast, other COF materials, such as Lyu's study [267], have been proven in a prior study to enhance CO_2_ absorption by 1360 times to 1.5 mmol/g; nevertheless, they were still unable to match the higher capacity and catalytic performance seen in the work. This material is thought to be less effective for large‐scale CO_2_ collection than the NH_2_‐IL functionalized COF described in the current work, despite reports of considerable enhancement in humid circumstances. Similarly, another study Qiu [269], developed fluorinated COFs for direct air capture (DAC) applications. However, it was discovered that the NH_2_‐IL functionalized COFs performed better than the fluorinated COFs, with a CO_2_ adsorption capacity of 2.13 mmol/g at 400 ppm. Yang et al. [278] recently presented NUT‐105@COF‐OCH_3_, a photo‐responsive composite material for controlled CO_2_ adsorption. This composite offers a major improvement in the control of light‐induced adsorption by combining a (COF‐OCH_3_) with a metal–organic cage (NUT‐105). The CO_2_ adsorption capacity was 33.3 cm^3^/g under visible light and dropped to 25.8 cm^3^/g under UV light, indicating a 29.2% change, as opposed to just 1.5% for bulk NUT‐105. The free isomerization of azobenzene (AZO) groups, which modifies the pore architectures and adsorption sites, is responsible for the improvement in adsorption. The light‐triggered adsorption mechanism was validated using grand canonical Monte Carlo (GCMC) simulations, demonstrating the material's potential for energy‐efficient CO_2_ capture. This breakthrough signifies an exciting change in CO_2_ capture technology by incorporating light‐responsive characteristics into conventional COF frameworks.

The hydroxyl‐functionalized ionic polymers created by Liao et al. [274] are shown to have the highest CO_2_ adsorption capacity when HCPs are compared, with a capacity of 3.58 mmol/g at 273 K and 1 bar. With surface areas of up to 1480 m^2^/g and functionalized ionic sites that are known to greatly increase CO_2_‐philicity, the material's vast microporous architecture is responsible for this performance. The dual functionality of CO_2_ capture and conversion is highlighted by the polymer produced by Liao, which is demonstrated to catalyze CO_2_‐epoxide reactions with a 99% yield in addition to its exceptional adsorption capacity. In terms of pure CO_2_ adsorption, this outstanding performance is believed to set HCPs ahead of COFs. This material is thought to be a perfect fit for applications that need to absorb CO_2_ and convert it catalytically because of its large surface area and functional sites. In general, CMPs have lower CO_2_ adsorption capabilities than COFs and HCPs. The nitrogen‐ and oxygen‐rich CMPs examined by Mohamed et al. [277] had a CO_2_ adsorption capacity of 1.85 mmol/g at 273 K and 1 bar. Despite having a lesser capacity than both COFs and HCPs, the CMPs showed outstanding thermal stability (Td10 of 505°C) and multifunctionality, which makes them useful for energy storage and catalysis in addition to CO_2_ capture. Although CMPs might not be able to meet the greatest CO_2_ adsorption capabilities, their resilience and high‐temperature tolerance make them appropriate for use in severe situations where stability is crucial.

On the other hand, Baig et al. [279] recently synthesized three conjugated POPs (C‐POP1−3) using a [3+2]. cyclocondensation procedure. Only at temperatures as high as 485°C did these polymers show a 10% weight loss, demonstrating their remarkable thermal resilience. The polymers' CO_2_ adsorption capacities differed; at 273 K, C‐POP1's CO_2_ absorption was 83.0 mg/g, while C‐POP2's BET surface area was 560 m^2^/g. The recyclability of these polymers was remarkable, with steady performance noted over seven cycles, despite their decreased CO_2_ uptake in comparison to COFs. Furthermore, C‐POP3 demonstrated exceptional iodine adsorption properties, attaining 2220 mg/g, underscoring the materials' adaptability for a range of uses. The comparative performance of these materials is evident, showing that HCPs, like those developed by Liao et al., achieve higher pure CO_2_ adsorption capacity, while COFs, especially the NH_2_‐IL functionalized COFs developed by Yin et al. [271] offer excellent CO_2_ adsorption capacities with catalytic benefits. With its large surface area and ionic functionalization, Liao's material demonstrates the tremendous potential of HCPs for CO_2_ collection, outperforming COFs in terms of adsorption efficiency. Although CMPs follow behind in CO_2_ adsorption, their benefits in thermal stability and multifunctionality make them appropriate for some applications where both CO_2_ collection and thermal resistance are needed.

Several studies have investigated novel POPs that show outstanding results not only for CO_2_ adsorption but also for separation and conversion, indicating the variety and ability of these materials in addressing CO_2_ emissions. These studies go beyond the specific categories of CO_2_ capture materials. TAPPy‐TFP, the best‐performing material among the β‐ketoenamine POPs synthesized by Zong et al. [280], achieved a CO_2_ uptake of 3.87 mmol/g at 273 K and 1 bar. In addition to having outstanding thermal stability and a high CO_2_/N_2_ selectivity of 27, this material‐maintained efficiency throughout 10 cycles and up to 400°C. Its potential for CO_2_ extraction from flue gas was demonstrated by breakthrough simulations, demonstrating its usefulness in real‐world situations where thermal stability and excellent selectivity are essential. Like this, Wu et al. [281], created pyridine‐based POPs with a dual‐functionality strategy that can be used for both CO_2_ capture and catalytic conversion. At 273 K, these materials' CO_2_ adsorption capacity was 2.95 mmol/g, and their CO_2_/N_2_ selectivity was 94. The material's scalability for CO_2_ capture is improved by a noteworthy pilot‐scale breakthrough experiment that showed a working capability of 20 L of flue gas per kg POP per hour. Furthermore, with a space‐time yield of 9.6 g/LPOP/day, the captured CO_2_ was successfully transformed into oxazolidinone, highlighting the POPs' potential for both CO_2_ capture and CO_2_ utilization in industrial applications. However, Yang et al. [282], emphasized on POPs modified with ethylenediamine (POP‐3), which had the greatest CO_2_ adsorption heat (Q_st_) of 54.8 kJ/mol of all the materials they examined. In dynamic breakthrough studies, POP‐3 showed an effective separation of CO_2_ from both N_2_ and CH_4_, as well as a CO_2_/N_2_ selectivity of 202.4. This material's remarkable reusability is demonstrated by the fact that it retained its adsorption capacity for at least 10 cycles. As demonstrated by in situ FTIR and DFT analysis, the higher performance was ascribed to the synergistic effect of primary and secondary amine groups, indicating that functionalization can be crucial in improving both CO_2_ adsorption and selectivity.

#### 5.1.2 Selectivity

Diverse approaches to enhance selectivity and permeability have demonstrated recent developments in CO_2_ separation membranes, with each study contributing distinct advantages and disadvantages. Zhang et al. [283], set a new standard for membrane technology when he used hybrid alkylamine‐modified PAF‐1 membranes to obtain a CO_2_ permeability of 10 790 Barrer and a CO_2_/N_2_ selectivity of above 1000. With a CO_2_/N_2_ selectivity of 28.5 and a CO_2_ permeability of 797 Barrer, the nanoporous fluorinated molecular sieve membranes in Yang's study [284] were demonstrated to surpass the Robeson upper bound, complementing this ground‐breaking study. The adaptability of molecular sieve designs has been highlighted, especially for applications that need a compromise between selectivity and permeability, even if Zhang's study's selectivity outperforms this one. Jia et al. [276], has also exhibited advancements in CMP membranes, reporting a CO_2_ permeance of 1134 GPU and a CO_2_/N_2_ selectivity of 22.5. The cost‐effectiveness of CMP membranes has been cited as a major benefit, even though their selectivity is lower than the values were reported in Zhang's and Yang's work. Qiu et al. [285] made a significant advancement by developing thin‐film nanocomposite membranes made of UiO‐66‐NH_2_ nanoparticles and carboxylated PIM‐1 (cPIM‐1). With stability over 63 days highlighted as a crucial attribute for real‐world applications, these membranes obtained a CO_2_ permeance of 2504 GPU, a CO_2_/N_2_ selectivity of 37.2, and a CO_2_/CH_4_ selectivity of 23.8. Additionally, Wu et al. [286] and Wang et al. [287] have made noteworthy contributions. While the COF‐316 membranes in Wang's study showed a CO_2_/N_2_ selectivity above 50 and a permeance of 500 GPU, the knitted microporous polymer membranes in Wu's study were reported to attain a CO_2_/CH_4_ selectivity of 27 and a permeability of 4651.2 Barrer. The customized qualities of these solutions were acknowledged as important for applications needing a balance of performance characteristics, even if they fell short of Zhang's revolutionary thresholds. Sekizkardes et al. [288] also has shown incremental improvement with PIM‐based membranes that showed a permeability of 1764 Barrer and a CO_2_/N_2_ selectivity of 39.

Chen et al. [289] proposed hyper‐crosslinked porous ionic polymers (HCPIPs), which resulted in CO_2_ adsorption capacities of 2.68–3.01 mmol/g and CO_2_/N_2_ selectivity of 166–237. Nevertheless, in contrast to hybrid and molecular sieve membranes, the absence of permeability optimization was identified as a drawback. Comparably, Li et al. [273] created polymeric membranes with a selectivity of 45 and permeabilities of 3500 GPU, offering competitive performance without going above and beyond the standards established by previous research. The adaptability of POPs in CO_2_ collection and conversion has been proved by recent advancements; numerous studies have focused on improving selectivity and catalytic activity to allow more effective CO_2_ control. These experiments demonstrate how POPs can bridge the gap between capture and usage by efficiently separating CO_2_ and facilitating its conversion into valuable products. Youm et al. [290], for instance, synthesized a series of porphyrin‐based azo‐POPs (PBPOP1, PBPOP2, and PBPOP3) that demonstrated notable CO_2_/N_2_ selectivity, with PBPOP3 exhibiting the highest selectivity of 534 at 313 K. What distinguishes this work, however, is the additional catalytic functionality of the polymers: the study demonstrated that these materials could not only adsorb CO_2_ but also catalyze its conversion into styrene carbonate through CO_2_ fixation processes with styrene oxide and phenyl glycidyl ether. Similarly, ionic (MCTFs, BCTFs, and YCTFs) were created by Wen et al. [291] and demonstrated outstanding CO_2_ adsorption selectivity and enrichment capacity. With a 96% yield and 99% selectivity in CO_2_‐propylene oxide coupling without the need of a solvent or cocatalyst, the MCTF‐10 version was particularly noteworthy. Surprisingly, MCTF‐10 retained strong catalytic activity and stability even in the face of more difficult circumstances, including gentler temperatures (80°C, 0.1 MPa) and low CO_2_ concentrations (15% CO_2_, 85% N_2_). Similar to the study of Youm et al., this work expands on the concept of multifunctionality by optimizing the material for CO_2_ conversion in addition to concentrating on CO_2_ adsorption, showcasing its potential for large‐scale applications in practical settings. By adding palladium nanoparticles to their POPs (POP‐n‐Pd) for the conversion of CO_2_ into oxazolidinones, Liang et al. [292] furthered the trend toward multifunctional POPs. Particularly, POP‐1‐Pd demonstrated exceptional catalytic performance, with a 96% yield in ambient settings. This catalyst performed exceptionally well in processing CO_2_ from lime kiln exhaust gas and showed extensive substrate application as well as recyclability over six cycles. POP‐1‐Pd's imidazole moieties and Pd NPs worked in concert to enable effective CO_2_ activation, highlighting POPs' potential for CO_2_ conversion. Like the research by Youm et al. and Wen et al., this study demonstrates that POPs can be used as both extremely effective catalysts for CO_2_ transformation and as efficient CO_2_ adsorbents. These investigations demonstrate how POPs' function in CO_2_ capture and conversion is changing. These materials provide a holistic strategy to tackling CO_2_ emissions by combining catalytic capability with high selectivity for CO_2_ adsorption. Every study advances our knowledge of how POPs can be customized for particular uses, offering a viable future for CO_2_ management plans that integrate capture and utilization.

#### 5.1.3 CO_2_ Conversion

Effective CO_2_ collection and catalytic conversion, with an emphasis on converting CO_2_ into useful chemicals such as cyclic carbonates, have been shown to depend on the design of porous materials. Numerous performance‐enhancing techniques have been brought to light by recent studies, with a focus on elements including surface area, adsorption capacity, recyclability, and catalytic efficiency.

There has been promise in the development of HCPs. Qu et al. [293] synthesized phenylpyridine‐ and phenyl‐based HCPs by one‐step Friedel‐Crafts polymerization. At 273 K and 0.1 MPa, it was shown that these materials' enormous specific surface area of 1133 m^2^/g could adsorb 3.1 mmol/g of CO_2_. For cycloaddition reactions between epoxides and ambient CO_2_, the high adsorption capacity led to efficient catalytic performance, producing remarkable quantities of cyclic carbonates. The utilization of tetrabutylammonium iodide (TBAI) as a co‐catalyst was shown to further increase the reaction efficiency. Remarkably, these catalysts remained active after five cycles, suggesting their durability and potential for use in industrial settings.

Li et al. [273] improved on this basis and achieved notable advancements in the field by developing the dual‐ionic system HIP‐COOH‐TMG specifically for CO_2_ cycloaddition. At 110°C and 1 MPa CO_2_ pressure, this catalyst produced a remarkable 99% chloropropene carbonate (CC) yield in just 8 h, demonstrating both high efficiency and mild reaction conditions. The catalyst's ease of separation and recycling demonstrated its promise for industrial application and further supported its feasibility for recurrent use. This system's focus on catalytic precision and rapid reaction kinetics provided an alternative perspective to Qu's study employing an adsorption‐oriented technique, even if it did not disclose adsorption capacities. These advancements are a part of a broader trend to combine high‐surface‐area materials with advanced functionalization to enhance adsorption and catalytic properties.

Representative catalytic mechanisms and structural schemes of these POP‐based systems are illustrated in Figure 18a–f. Figure 18a shows the geometrical optimization and minimum energy path profile for the TPA‐HCP‐Py/TBAI‐catalyzed cycloaddition reaction between epichlorohydrin and CO_2_, where the color‐coded atoms highlight the active interaction sites. Figure 18b illustrates the detailed reaction mechanism of the TPA‐HCP‐Py/TBAI synergistic catalysis, emphasizing the cooperative role of TBAI in lowering the activation barrier. Figure 18c presents the calculated interactions of the HIP‐COOH‐TMG pair with CO_2_, including (a) the N‐to‐CO_2_ interaction of TMG and (b) the N‐to‐CO_2_ interaction of imidazole, which clarify how multiple active centers enhance CO_2_ binding. Figure 18d depicts the plausible reaction mechanism for CO_2_ cycloaddition to epoxide over HIP‐COOH‐TMG, highlighting the role of ionic and hydrogen‐bonding sites in stabilizing intermediates. Figure 18e shows the proposed reaction pathway for the cycloaddition of CO_2_ and styrene oxide catalyzed by the dual hydroxyl‐functionalized H‐[BnDHPIm]Br/BPh_4_‐FDA + TBABr system, where hydroxyl groups and ionic moieties synergistically promote the reaction. Finally, Figure 18f displays the synthetic scheme of natural‐abundance and carbonyl‐^13^C‐labeled COFs, which enables isotopic tracking for mechanistic investigation. In addition, the schematics highlight how dual functionalization, and cooperative catalytic sites can simultaneously enhance both adsorption strength and conversion selectivity, ensuring efficient utilization of captured CO_2_. Qu's method demonstrated how material properties may be changed to provide a variety of advantages, whereas Li's system focused on catalytic speed and product yield. Notably, these materials' durability and reusability are critical to their possible uptake. The endurance of these catalysts under operating conditions is demonstrated by the fact that Qu's HCPs have retained their performance across five cycles, while Li's dual‐ionic system has demonstrated the capacity to be recycled. The findings collectively show the significance of creating porous materials which provide a compromise between high absorption of CO_2_ and effective catalytic conversion, opening the door to new ecologically friendly CO_2_ use techniques. Table 4 summarizes the structural characteristics, CO_2_ uptake capacities, and catalytic efficiencies of various porous materials, offering a comparative perspective on their potential for practical application.

FIGURE 18(a) Geometries optimization and the minimum energy path profile for the TPA‐HCP‐Py/TBAI‐catalyzed cycloaddition reaction between epiohydrin and CO_2_. (C—green, H—gray, O—red, N—blue, Br—purple, I—pink. Reproduced with permission from ref. [293]. Copyright 2023, Elsevier. (b) Reaction mechanism of TPA‐HCP‐Py/TBAI synergistic catalysis [293]. (c) The calculated interaction of the HIP‐COOH‐TMG pair CO_2_ a: N to CO_2_ interaction of TMG and, b: N to CO_2_ interaction of imidazole. Reproduced with permission from ref. [273]. Copyright 2024, Elsevier. (d) Plausible reaction mechanism for the CO_2_ cycloaddition to epoxide over HIP‐COOH‐TMG [273]. (e) Proposed reaction mechanism for the cycloaddition reaction of CO_2_ and SO catalyzed by the dual hydroxyl‐functionalized H‐ [BnDHPIm]Br/BPh4‐FDA + TBABr. Reproduced with permission from ref. [294]. Copyright 2024, Royal society of chemistry. (f) Synthetic scheme of natural abundance and carbonyl‐^13^C‐labeled COF. Reproduced with permission from ref. [267]. Copyright 2022, American Chemical Society.

**Figure:**
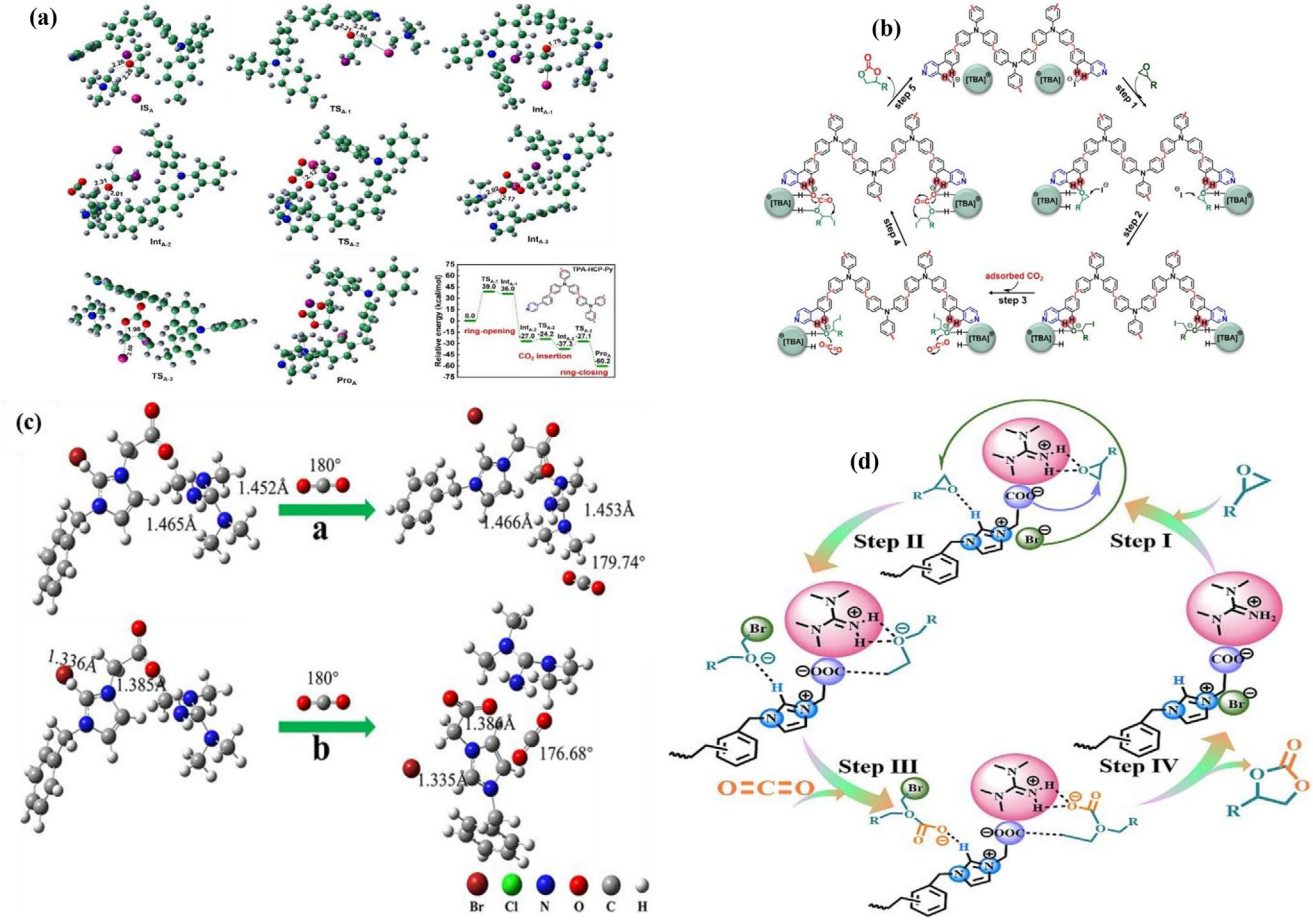

**Figure:**
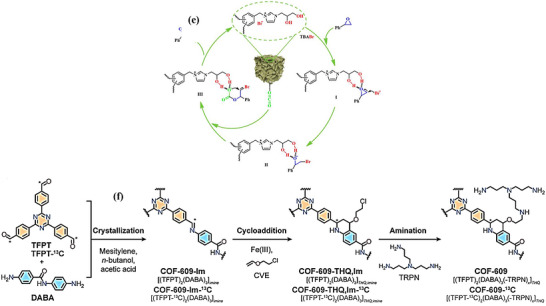

**TABLE 4:** Performance comparison of porous materials for CO_2_ capture and catalytic conversion.

Polymer ID	S_BET_ (m^2^.g^−1^)	V_tot_ (cm^3^.g^−1^)	V_micro_ (cm^3^.g^−1^)	D_ave_ (nm)	CO_2_ uptake @273K (mg. g^−1^)	ΔH° (kJ.mol^−1^)	Yield of EC (%)	Selectivity of EC (%)	Refs.
HSPC‐12H‐6	705	0.34	0.20	—	95.06	16.83‐29.11	—	—	[295]
HSPC‐24H‐6	825	0.40	0.23	—	89.98	22.42–29.11	—	—	[295]
LSPC‐12H‐6	423	0.21	0.12	—	72.30	14.70–26.58	—	—	[295]
LSPC‐24H‐6	469	0.22	0.13	—	70.39	22.06–49.25	—	—	[295]
PDVB	653	1.21	—	—	—	—	—	—	[296]
POP‐1	214	0.47	0.03	8.6	—	—	>99	—	[296]
POP‐2	305	0.25	0.01	4.0	—	—	>99	—	[296]
POP‐3	234	0.72	0.02	30.5	95.06	54.8	>99	—	[296]
POP‐3′	474	0.87	0.01	—	89.98	—	>99	—	[296]
POP‐4	298	0.83	0.01	42.6	72.30	—	>99	—	[296]
POP‐5	168	0.19	0.02	3.50	70.39	—	>99	—	[296]
POP‐5′	505	1.41	0.03	—	—	—	>99	—	[296]
HX‐BP (1:3)	1460	1	0.67	4.25	157.1	−17.115	99	>99	[274]
HX‐BP (1:2)	1487	1.48	0.67	4.91	—	—	89	>99	[274]
HX‐BP (1:1)	1329	1.08	0.60	3.83	—	—	95	98	[274]
BI‐BP (1:3)	1399	1.40	0.65	5.51	—	– 17.045	71	>99	[274]
HX‐DCX (1:3)	804	1.00	0.38	8.20	—	−17.621	73	>99	[274]
HX‐DBX (1:3)	558	0.74	0.27	9.13	—	−14.791	58	>99	[274]
HBP1	1254	1.03	0.59	4.69	—	−16.622	<1	—	[274]
HCIP–1	639	0.83	0.19	—	95.06	−13.51	89	>99	[297]
HCIP‐2	1448	1.37	0.35	—	117.08	−15.46	76	>99	[297]
HCIP‐3	659	1.00	0.17	—	81.42	−12.98	87	>99	[297]
HCIP‐4	1435	1.55	0.37	—	121.03	−15.99	22	>99	[297]
Polymer 1	606	0.52	0.14	—	98.2	28.15–23.85	—	—	[275]
Polymer 2	942	0.66	0.22	—	121.2	28.21–25.85	—	—	[275]
Polymer 3	1367	0.84	0.34	—	146.1	27.68–25.28	—	—	[275]
P(M1/TEB1:1)	832	0.90	0.29	0.9	—	—	—	—	[298]
P (M1/B 1:1) H	911	0.60	0.35	0.7	99	26	—	—	[298]
P (M1/B 3:1)	313	0.19	0.12	—	—	—	—	—	[298]
P (M1/B 3:1) H	534	0.28	0.22	0.7	87.6	29	—	—	[298]
P (M2/B 1:1)	393	0.71	0.13	1	—	—	—	—	[298]
P (M2/B (1:1) H	794	0.76	0.29	0.7	72.6	26	—	—	[298]
P (M2/B 3:1)	149	0.11	0.05	1.1	—	—	—	—	[298]
P (M2/B 3:1) H4	409	0.22	0.17	0.7	75.2	27	—	—	[298]

Comparing with conventional porous adsorbents is useful for placing the CO_2_ capture performance of POPs in a meaningful context. Activated carbons are low‐cost and typically have good surface areas, but their weakly functionalized surfaces can limit CO_2_ selectivity. Zeolites usually have a high affinity for CO_2_ due to their polar structures. However, their performance may be compromised by water adsorption in humid environments. MOFs exhibit very high porosity and well‐defined pore environments, but the stability of some MOF structures remains a concern in gas streams containing moisture. POPs have some advantages, such as covalent framework structures, flexible pore design, and relatively facile functionalization. These features allow them to combine competitive CO_2_ uptake and selectivity with good chemical, thermal, and hydrothermal stability. Their generally moderate adsorption enthalpies are also advantageous, as they can help lower the energy required for regeneration during cyclic CO_2_ capture [61].

### 5.2 Catalysis and Photocatalysis

Conjugated porous polymers (CPPs) are being rapidly advanced as key materials in heterogeneous catalysis and photocatalysis, with targeted applications such as pollutant degradation, hydrogen production, and CO_2_ reduction being enabled by their diverse structures. At the forefront, exceptional efficiency in CO_2_ reduction has been demonstrated by the Ni–N_2_O_2_ single‐atom catalyst (SAC) framework studied by Dong et al. [299], achieving a CO generation rate of 7.77 mmol g^−1^ with 96% selectivity over H_2_. This precision has been attributed to enhanced ^*^COOH adsorption and charge transport properties, and a benchmark for carbon capture and utilization has been set. While unparalleled performance for CO_2_‐specific applications is shown by this material, a lack of versatility is highlighted in contrast with the sulfone‐based conjugated porous organic polymer (CPOP) (APS‐TFP _Flow_), in which pollutant degradation and hydrogen production have been combined by Singh et al. [300]. Methylene blue (MB) degradation of 96% and hydrogen production of 77 mmol/g_cat_/h from PET waste using a continuous flow synthesis method have been achieved, showcasing scalability and adaptability. However, hydrogen efficiency in Singh's study has been outperformed by Shu's work [301], where a D–π–A copolymer (PyBS‐3) has achieved a hydrogen evolution rate of 1.05 mmol h^−1^ with a quantum efficiency of 29.3% at 420 nm. The donor‐to‐acceptor ratio has been focused on in the precise molecular design, allowing light‐to‐hydrogen conversion to be optimized, but multifunctionality to be sacrificed. Interestingly, while hydrogen production has been targeted by the materials were used in Shu's and Singh's work with differing priorities—efficiency vs. versatility—the gap has been bridged by Kumar et al. [302] using the heptazine‐based HEP‐BTET polymer, which addresses sustainability. A CO evolution rate of 8.83 mmol g^−1^ h^−1^ with a quantum yield of 14% at 500 nm has been achieved by this material, and metals and sacrificial agents have been avoided, making it an eco‐friendly alternative to the resource‐intensive framework studied in Dong's work. Together, a cohesive narrative of advancing catalytic science is formed by these CPPs, where innovations in design and synthesis are allowing tailored solutions to complex environmental and energy challenges to be pushed, and the boundaries of functionality, efficiency, and eco‐consciousness are being extended.

The versatility of COFs in photocatalytic applications is highlighted by studies, and a closer analysis is provided to reveal how their designs, modifications, and resulting efficiencies are interrelated, offering insights into the approaches that hold the most promise. At the core of these advancements is the ability of COFs to be used as stable, tunable, and efficient platforms for light‐driven reactions, including CO_2_ reduction, water splitting, and hydrogen evolution [303]. The performance of COFs is determined by their structural design and functional modifications. For example, thermal stability and effective CO_2_ capture for methanol production under visible light without sacrificial agents are leveraged in azine‐based COFs studied by Fu et al. [304]. A benchmark for sustainable and metal‐free CO_2_ reduction is set by this work. In comparison, CO_2_ reduction is further advanced by the introduction of single‐atom MoN_2_ sites into COFs by Kou et al. [305], enabling selective hydrocarbon production, including CH_4_ and C_2_H_4_, with enhanced catalytic activity confirmed by theoretical calculations and in situ FT‐IR. Emphasis is placed on CO_2_ capture and light‐driven reduction in both studies, but differences in target products—methanol vs. hydrocarbons—highlight the flexibility of COF platforms to achieve different goals depending on the application. Bipyridine‐based COFs (Bp‐COFs) in Chen's work [306] and donor‐acceptor imine‐linked COFs by Yang et al. [307] illustrate the significance of energy band alignment and structural changes in hydrogen evolution. According to Chen's findings, coordination with Co^2+^ leads to high oxygen evolution rates, which are explained by light‐harvesting and charge transfer. Yang's research shows that protonating Schiff‐base bonds causes light absorption to redshift, which greatly accelerates the development of hydrogen. Although Chen's study exhibits higher adaptability due to their simultaneous generation of hydrogen and oxygen, these methods highlight the significance of chemical modifications to improve light usage and charge separation. The octupolar conjugated structure of Xu's study [308], tricyanomesitylene‐based COFs, enhances charge separation and semiconducting characteristics, resulting in remarkable performance for water‐splitting applications. This performance is like that of Wan's study [309], nitrogen‐linked COFs, which have bandgaps adjusted for best alignment with water redox potentials. Although the efficiency of both systems is well known, COFs in Xu's study are demonstrated to have higher rates of O_2_ and H_2_ evolution, which makes them more viable for large‐scale water‐splitting applications. Another important issue is the function of composites and heterostructures. Wang et al. [310] integrates Tp‐Tta COFs with nitrogen‐vacancy‐rich g‐C_3_N_4_ to create a van der Waals heterojunction, which maximizes charge transfer and enhances CO selectivity. Similarly, Lu's study [311], combination of polyoxometalates (POMs) with COFs allows selective CO_2_ photoreduction with about 100% selectivity. These findings demonstrate the synergistic enhancement of catalytic performance by hybrid systems, where Lu's strategy excels in selective hydrocarbon synthesis, and Wang's design provides a channel for CO_2_‐to‐CO conversion. Scalability and post‐synthesis changes are also highlighted. Yang's [312], conversion of N‐acylhydrazone‐linked COFs into stable π‐conjugated structures quadruples hydrogen evolution performance, and Zhou's [313] mesopore stabilization with PEG preserves charge transport pathways, significantly increasing hydrogen evolution rates. Lv's [314] mechanochemical synthesis method introduces a sustainable methodology that achieves high photocatalytic activity while requiring less solvent and time. These developments bridge the gap between lab‐scale performance and industrial scalability, improving the usefulness and efficiency of COFs. The benefits of integrating metal nanoparticles for visible‐light‐driven CO_2_ reduction are demonstrated by Ru‐functionalized COFs, such as Guo's study [315], Ru/TpPa‐1, where higher charge carrier lifetimes and activity are achieved compared to bare COFs. This aligns with the work of Zhong [316], where metalloporphyrin‐based COFs exhibit 98% selectivity for CO_2_‐to‐CO conversion, illustrating that catalytic efficiency is consistently improved by metal integration, particularly for CO_2_ reduction.

In the meantime, a number of advances are being made to investigate the potential of hyper‐crosslinked ionic polymers (HIPs) in CO_2_ conversion. According to Jiang et al. [317], these polymers may support CO_2_ cycloaddition processes with high yields (93%) and outstanding stability across several cycles. These HIPs are particularly appealing for environmentally friendly and sustainable CO_2_ collection and conversion applications due to their high surface area of 542.2 m^2^/g and lack of metals and solvents. Using a one‐pot Friedel‐Crafts alkylation and quaternization procedure, Wang et al. [318] produced amino acid‐based HIPs, producing polymers with high nitrogen functionality. The most efficient form, HIP‐His‐1, demonstrated 99% yield and selectivity for CO_2_ cycloaddition to epoxides under moderate, solvent‐ and metal‐free conditions. These HIPs had a very high CO_2_ absorption capacity of 25.17 cm^3^/g. Even in a combination of 15% CO_2_ and 85% N_2_, the polymer demonstrated exceptional performance. Additionally, the polymer showed outstanding reusability after five catalytic cycles, maintaining 91% activity and good selectivity. The effectiveness of the material was further explained by theoretical investigations that emphasized the critical role that nucleophilic anions and hydrogen‐bond donors play in the catalysis.

Conversely, the discovery of PAFs, such PAF‐6, shows promise for nonmetallic catalysts with great recyclability. PAF‐6's steady catalytic activity over ten cycles and its exfoliation into nanosheets demonstrate the adaptability of porous organic materials in hydrogenation processes. In contrast, Zn‐POP and Co‐POP, two metalated POPs created by Paul et al. [319], exhibit promise in CO_2_ reduction processes. Zn‐POP favors hydrogen synthesis, whereas Co‐POP promotes CO creation, demonstrating the significance of metal coordination in adjusting catalytic activity.

Finally, CTFs have become a powerful platform for a number of uses, such as CO_2_ fixation and photocatalytic water splitting. For example, Wang et al. [320] created amide‐functionalized nanosheets that demonstrated improved absorption of visible light and high hydrogen evolution rates of 512.3 mmol·h^−1^, highlighting the significance of structural optimization in photocatalytic applications. This illustrates how CTFs can be used in sustainable processes and energy conversion. Another study by Ping et al. [321] investigated squaramide‐functionalized covalent triazine framework (SCTFs) modified with squaramide moieties specifically for low‐concentration CO_2_ fixation, expanding on the idea of functionalized CTFs. These SCTFs showed remarkable surface areas (605–833 m^2^/g) and a significant affinity for CO_2_ after being manufactured utilizing a ZnCl_2_‐mediated trimerization process. At 273 K and 1.0 bar, the SCTFs' CO_2_ adsorption capacity reached 2.5 mmol/g, demonstrating their efficacy in CO_2_ capture. Additionally, in mild, metal‐/solvent‐free conditions, the SCTF‐CF_3_ in conjunction with KI allowed the conversion of CO_2_ into bromopropene carbonate with a 96% yield and 99% selectivity. Additionally, the technology demonstrated outstanding reusability. The squaramide moieties were found to be essential to the reaction mechanism by in situ FT‐IR and DFT investigations, demonstrating how functionalization can greatly improve CO_2_ collection and conversion.

Advancements in catalytic and photocatalytic systems are represented by these materials, with each offering specific advantages depending on the desired application. Visible‐light‐driven hydrogen generation is particularly strong in MOFs, while hyper‐crosslinked ionic polymers lead in CO_2_ conversion. Recyclability and nonmetallic efficiency are offered by PAFs, tunable catalytic activity for CO_2_ reduction is provided by metalated POPs, and excellent photocatalytic properties for water splitting are presented by CTFs. The particular catalytic or photocatalytic need, whether energy production, CO_2_ conversion, or environmental remediation, determines the choice between these materials. Each material's unique set of strengths can be leveraged for different sustainable technologies, making the ongoing exploration of these frameworks crucial for advancing clean energy and environmental solutions.

The promise of asymmetric photocatalysis with heterogeneous nanomaterials for the sustainable and ecologically friendly synthesis of enantiomerically pure compounds is presented by Qiu et al. [322]. Two primary categories of nanomaterials are discussed in this review paper: the development of porous materials, including MOFs, POPs, and organic cages, as well as conventional inorganic semiconductors like TiO_2_ and quantum dots. The benefits of heterogeneous catalysis, such as its ease of separation, recovery, and reuse, are outlined, alongside the difficulties and upcoming advancements in this field. The adaptability and potential for effective photocatalytic CO_2_ reduction and other associated applications are highlighted by recent developments in porphyrin‐based POPs. A variety of metal‐free POPs were created by Boruah et al. [323], and after 2 h of light illumination, POP‐1 showed the greatest CO_2_‐to‐CO conversion rate at 518 µmol g^−1^ h^−1^. While in situ FT‐IR and ultrafast transient absorption spectroscopy provided deeper insights into reaction kinetics and surface interactions, DFT calculations were also used in the work to identify the reaction mechanism. By concentrating on the impacts of keto‐enol tautomerization, this structural engineering approach improved the efficiency of CO_2_ photoreduction in metal‐free systems and helped to clarify the links between structure and properties. Chakrabortty et al. [324] concentrated on copper‐loaded catalysts deposited on porphyrin‐based POPs (TpTph/TpPOP) for CO_2_ reduction to methanol and photocatalytic hydrogen evolution. Without the use of a cocatalyst, the system demonstrated a high output of 598 µmol of hydrogen and a CO_2_‐to‐methanol conversion rate of 1.8 mol/g_cat while employing 15 W LED light at ambient temperature and atmospheric pressure. The sustainability and effectiveness of this convergent photocatalytic system were highlighted by the insightful mechanism revealed by both DFT and experimental investigations. Zhang et al. [325] presented hollow microspheres functionalized using porphyrin moieties for continual and sustained photocatalysis, thereby expanding the adaptability of porphyrin‐based materials. The positively charged spots on these microspheres successfully drew in anionic reactants, facilitating effective adsorption, reaction, and releases cycling. Methyl orange (MO) was completely degraded by this system in 40 min, and following six cycles under one sun's light, there was no more performance deterioration. Significant sustainability gains are offered by the self‐renewing photocatalytic system, which also holds promise for large‐scale industrial photocatalysis, photosynthesis, and photodegradation applications.

Recent developments in CO_2_ conversion indicate a variety of creative approaches to increase sustainability and catalytic efficiency. From metal‐free photocatalysts to metal‐coordinated systems and multifunctional catalysts, researchers have investigated a variety of strategies, all of which have helped to create more effective and ecologically friendly CO_2_ usage techniques. The application of porphyrin‐based POPs in metal‐free photocatalytic CO_2_ reduction, for example, was the subject of one study. Under light irradiation for 2 h, the study achieved an outstanding CO_2_‐to‐CO conversion rate of 518 µmol g^−1^ h^−1^ by optimizing the structure of the material [323]. Ultrafast transient absorption spectroscopy and in situ FT‐IR offered insightful information about the reaction kinetics, emphasizing the potential of structural engineering to improve photocatalytic efficiency through processes like keto‐enol tautomerization. However, another study investigated Zn@BP‐POP, a metal‐coordinated POP that demonstrated exceptional CO_2_ photoreduction to CO in ambient settings. By suppressing hydrogen evolution (HER) and improving CO selectivity, the addition of zinc sites to the framework increased catalytic activity. The critical function of zinc in CO_2_ activation was validated by X‐ray absorption spectroscopy (XAS) and time‐dependent DFT analysis, highlighting the potential of metal‐active sites in enhancing photocatalytic systems [326]. Additionally, investigating multifunctional catalysts has shown promise as a means of combating CO_2_ conversion and environmental contamination. Bifunctional photocatalysts made from POPs based on cyclophosphazene and covered with GO and silver nanoparticles were shown in a work by Wang et al. [327]. These catalysts produced notable CO_2_ photoreduction yields and demonstrated outstanding antibiotic removal. Interestingly, combining pollutant degradation and CO_2_ reduction increased CO yield even more, showing how multifunctional catalysts can use pollutant‐derived electron donation to boost performance. The effect on the environment of NiAl‐LDH and Co‐ZIF‐9 catalysts for CO_2_ photocatalysis was compared in Wang's study in terms of environmental sustainability [328]. According to the findings, Co‐ZIF‐9 is a more sustainable option for CO_2_ conversion operations because it has a smaller environmental impact. A sensitivity analysis gave valuable insights for industrial‐scale application by highlighting the significance of catalyst recyclable in optimizing process efficiency. Finally, Wu et al. [329] reviewed the ecologically friendly substitute for the conventional phosgene process, the cycloaddition of CO_2_ with propylene oxide (PO) to create propylene carbonate (PC). Prioritizing the importance of multifunctional catalysts for activating PO and CO_2_ adsorption sites, the review examined the function of both homogeneous and heterogeneous catalysts in the process. The future of CO_2_ usage technologies was thoroughly examined, with issues including equipment, CO_2_ source, and catalyst/product separation were included. These studies demonstrate the advancements in CO_2_ conversion research and the variety of approaches that are enhancing scalability, sustainability, and efficiency. The continuous development of CO_2_ conversion technology has considerable promise for tackling global environmental concerns, whether through multifunctional methods, metal‐coordinated catalysts, or metal‐free systems.

**FIGURE 19:**
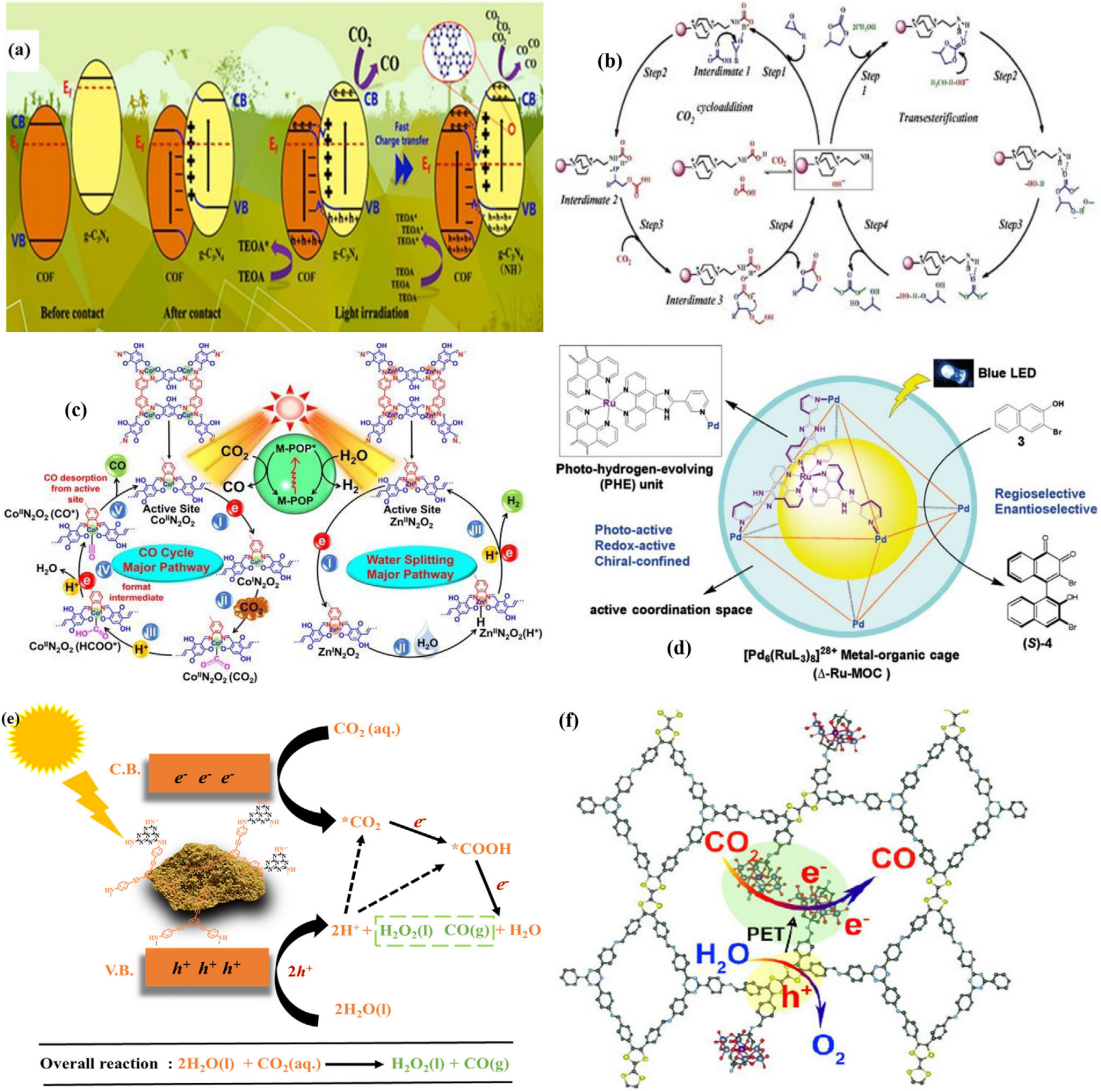
(a) Schematic illustration of the relative band energy positions and S‐scheme charge transfer mechanism between Tp‐Tta COF and g‐C_3_N_4_ (NH). Reproduced with permission from ref. [310]. Copyright 2022, Elsevier. (b) The proposed mechanism of CO_2_ cycloaddition and transesterification. Reproduced with permission from ref. [317]. Copyright 2023, Elsevier. (c) Proposed mechanism for CO_2_ reduction to CO and H_2_O splitting to H_2_ by Co‐POP & Zn‐POP, respectively based on DRIFTS and DFT studies. In the mixed catalyst, both the process can be activated, and syngas can be obtained with a desirable ratio from the same photo‐reactor upon light irradiation, just in the presence of water. Reproduced with permission from ref. [319]. Copyright 2023, Wiley‐VCH. (d) Schematic representation of homochiral photoactive metal–organic cage (MOC‐16) and the photoinduced biaryl coupling reaction. Reproduced with permission. Copyright 2017, Wiley‐VCH [322]. (e) Proposed reaction mechanism for the photocatalytic CO_2_ reduction in water. Reproduced with permission from ref. [302].Copyright 2024, Royal society of chemistry. (f) Schematic of the mechanism of TCOF‐MnMo6 for CO_2_RR coupled with H_2_O oxidation. Reproduced with permission from ref. [310]. Copyright 2022, Elsevier.

The mentioned studies demonstrate how advances in material design, hybridization strategies, and structural modification are driving improvements in efficiency, selectivity, and sustainability across catalytic and photocatalytic systems. To provide a clearer understanding of these developments, Figure 19 presents representative schematic illustrations of the underlying mechanisms. Figure 19a shows the relative band energy positions and the S‐scheme charge transfer mechanism between T_p_‐T_ta_ COF and g‐C_3_N_4_ (NH), which demonstrates how band structure engineering enhances charge separation and suppresses recombination, thereby improving photocatalytic efficiency. Figure 19b illustrates the proposed mechanism of CO_2_ cycloaddition and transesterification, highlighting how cooperative catalytic sites facilitate the activation of CO_2_ and epoxides, lowering the reaction barrier and enabling efficient product formation. Figure 19c depicts the dual role of Co‐POP and Zn‐POP in driving CO_2_ reduction to CO and H_2_O splitting to H_2_, respectively. DRIFTS and DFT analyses confirm that in the mixed catalyst both processes can occur simultaneously, yielding syngas with a tunable ratio — a promising route for integrated fuel production. Figure 19d presents a homochiral photoactive metal–organic cage (MOC‐16) that drives photoinduced biaryl coupling reactions. The stereochemical control in this design shows how molecular chirality and cage geometry influence photocatalytic selectivity. Figure 19e demonstrates the proposed pathway for photocatalytic CO_2_ reduction in water, emphasizing how aqueous‐phase photocatalysis can exploit water as both a medium and an electron donor, thus enhancing sustainability. Finally, Figure 19f illustrates the mechanism of TCOF‐MnMo_6_ for CO_2_ reduction reaction (CO_2_RR) coupled with H_2_O oxidation, showcasing the multifunctionality of porous COF frameworks in integrating reduction and oxidation processes within a single photocatalyst.

### 5.3 Energy Storage and Conversion

#### 5.3.1 POPs in Ion‐Based Batteries Cathode/Anode

Rechargeable batteries have become increasingly important for energy storage, yet there remains considerable potential for enhancing their efficiency, particularly energy density and capacitance [330]. Advancements in electrode materials are essential to improving performance, with a focus on durability, cost‐effectiveness, and sustainability [331]. The core mechanism governing rechargeable battery function hinges on the transport of ions through the electrolyte between the electrodes during charge–discharge transitions [332]. This ion movement drives redox processes at the interface between electrodes and the electrolyte, enabling energy conversion between chemical and electrical forms [333]. In lithium‐ion batteries (LIBs), energy storage is regulated by reversible redox reactions occurring at both the anode and cathode. During the charging phase, lithium ions migrate from the positive electrode to the negative electrode via the electrolyte, while electrons traverse an external pathway. During discharge, the directional flow of ions and electrons is inverted, facilitating energy release. The overall redox reactions involved in a lithium‐ion battery can be represented by Equations (1) and (2) [330].

(1) $$\mathrm{Li}C_{6}↔Li^{+}+e^{-}+C_{6}$$

(2) $$Li_{x}\mathrm{Co}O_{2}+Li^{+}+e^{-}↔Li_{x+1}\mathrm{Co}O_{2}$$

**FIGURE 20:**
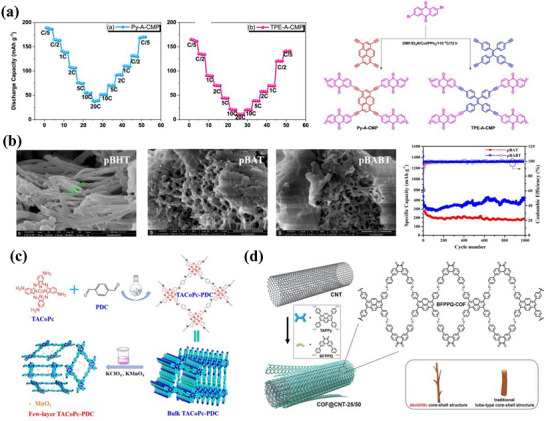
(a) A schematic representation of the synthetic route for Py‐A‐CMP and TPE‐A‐CMP. The rate performance of assembled cells incorporating these materials was evaluated across a range of current densities from 0.2C to 20C, along with Nyquist plots depicting their electrochemical behavior. Reproduced with permission from ref. [335]. Copyright 2021, American Chemical Society. (b) SEM images displaying the morphological features of pBHT, pBAT, and pBABT. The long‐term stability of pBAT and pBABT electrodes was investigated at a 500‐mA g^−^^1^ current density over 1000 cycles, alongside an assessment of their rate capability. Reproduced with permission from ref. [337]. Copyright 2020, Elsevier. (c) A graphical depiction of the synthesis process and exfoliation strategy employed for TACoPc‐PDC. Reproduced with permission from ref. [341]. Copyright 2024, Elsevier. (d) A schematic overview illustrating the in situ growth strategy for synthesizing BFPPQ‐COF and its COF@CNT composite. Reproduced with permission from ref. [339]Copyright 2023, Wiley‐VCH.

The electrochemical stability of POPs is primarily attributed to their strong covalent frameworks (C–C, C–N, C–O), which improve structural resilience and inhibit bond cleavage during charge–discharge cycles. This structural integrity ensures long‐term stability and preserves electrochemical performance [120]. Despite these advantages, POP‐based electrode materials face challenges, particularly the dissolution of organic molecules in electrolytes during cycling [334]. Two COFs, Py‐A‐CMP and TPE‐A‐CMP, were developed incorporating anthraquinone‐functionalized structures as electrochemically active sites [335]. These CMPs, defined by active C = O groups, low solubility, and extensive conjugation, exhibited specific capacities of 196.6 and 164.7 mAh/g under a 0.1C rate, as illustrated in Figure 20a, along with remarkable rate performance in Li‐ion cells. Their porous architectures and robust covalent frameworks enhance structural integrity by minimizing dissolution.

High specific surface area is often considered beneficial for battery performance [336]; however, Xu et al. challenged this assumption [337]. They synthesized conjugated polymers based on benzene‐thiophene (pBHT) and benzene‐ethynyl‐thiophene (pBAT and pBABT) frameworks with a star‐like configuration. The pBHT polymer, despite featuring an extensive surface area (∼1139 m^2^/g) and a distinct stacked hollow‐tube morphology, exhibited inadequate electrochemical performance, delivering a discharge capacity below 100 mAh/g with rapid capacity degradation and restricted cycling stability. This behavior was attributed to restricted lithium‐ion release, resulting in the development of “dead lithium,” polymer structure collapse, and inadequate solid electrolyte interface formation. In contrast, pBABT, with a notably smaller surface area (12.5 m^2^/g), demonstrated a discharge capacity of 442 mAh/g at 500 mA/g after the second cycle and maintained consistent performance over 1000 cycles (401 mAh/g at 500 mA/g) (Figure 20b). These results indicate that the morphology of the polymer and the mechanisms of ion transport are pivotal factors in influencing electrochemical performance, extending beyond surface area alone.

To develop cost‐effective and practical electrode materials from POPs, strategic material design and structural enhancements are necessary. In contrast to their 2D and 3D equivalents, 1D COFs are created by chemically bonding structural units along a single axis, forming porous nanobelts through a combination of weak van der Waals interactions, *π*–*π* interactions, and hydrogen bonding [338]. These non‐covalent interactions, being significantly weaker than covalent bonds, facilitate the dispersion of 1D COFs and the exposure of active sites, rendering them promising for electrode applications [147]. Jia et al. [339]. A redox‐active 1D COF and its composite with 1D CNTs were synthesized through in situ growth, as illustrated in Figure 20c. When utilized as cathode materials for LIBs, the 1D COF@CNT composites, characterized by a distinctive dendritic core–shell configuration, offered numerous readily available redox‐active sites, facilitating lithium‐ion transport and improving specific capacity This well‐integrated structural design resulted in exceptional electrochemical properties, including 95% redox site utilization, excellent rate capability (81% capacity retention at 10C), and long‐term cycling stability (86% capacity retention after 600 cycles at 5C).

COFs often exhibit slow lithium‐ion diffusion, limited electrical conductivity, and inefficient utilization of active sites, restricting their real‐world applicability [340]. To address these limitations, Liu et al. [341] developed TACoPc‐PDC/MnO_2_ composites were synthesized using a strong oxidant intercalation method to exfoliate 2D COFs (TACoPc‐PDC) effectively. During this process, MnO_2_ nanoparticles were formed in situ, serving as spacers to inhibit the re‐aggregation of TACoPc‐PDC and maintain its thin‐layered architecture throughout repeated charge–discharge cycles. The exfoliated TACoPc‐PDC/MnO_2_ exhibited a reduced‐layer lamellar configuration, which increased the density of accessible active sites and enhanced electrochemical properties. Compared to bulk TACoPc‐PDC, the exfoliated composite demonstrated significantly greater capacity, superior cycling stability, and enhanced rate performance. In LIBs, the initial capacities of TACoPc‐PDC and TACoPc‐PDC/MnO_2_ were recorded at 194 and 932 mAh/g, respectively, under a current density of 100 mA/g. After 400 cycles, these values stabilized at 648 and 1100 mAh/g. Even at an elevated current density of 5 A/g, the TACoPc‐PDC and TACoPc‐PDC/MnO_2_ electrodes retained specific capacities of 67 and 307 mAh/g, respectively. (Figure 20d). The design and synthesis strategies significantly influence the electrochemical performance of POPs in cathode and anode ion batteries. Recent research has introduced novel POPs alongside advanced synthesis and design methodologies aimed at enhancing their electrochemical properties. Table 4 provides a detailed comparison of these newly developed POPs and their corresponding electrochemical performance improvements.

#### 5.3.2 Solid‐State Electrolytes Based POPs

Solid‐state electrolytes (SSEs) are essential for high‐energy, long‐cycle rechargeable batteries. However, they face challenges such as capacity fading and safety risks caused by inhomogeneous lithium deposition [342]. Polymer electrolytes improve electrode contact and reduce interfacial impedance, making them a promising alternative [343]. Nevertheless, their low ionic conductivity and poor mechanical stability limit practical applications, necessitating advanced polymer chemistries for stable battery performance [344]. COFs provide an ideal platform for single Li‐ion conduction, leveraging their highly ordered one‐dimensional channels and tailorable chemical structures. Zhao et al. [345] introduced the first instance of a polyarylether‐linked COF functionalized with carboxylic acid sodium (referred to as NaOOC‐COF), designed as a next‐generation Na‐ion quasi‐solid‐state conductor film. Their research reveals that NaOOC‐COF, owing to its well‐structured ion pathways, achieves a remarkable Na^+^ conductivity of 2.68 × 10^−4^ S cm^−1^ at room temperature, exhibiting a low activation energy (E_a_ = 0.24 eV), and an impressive transference number of 0.9. Furthermore, its long‐term cycling stability in a quasi‐solid‐state battery system is demonstrated, effectively inhibiting dendrite formation through interface modulation. The study also presents a detailed analysis of the Na^+^ diffusion dynamics within the entire cell system, underscoring its applicability in solid‐state sodium‐ion battery technologies.

Dendrite growth is a critical issue in SSEs that compromises battery safety and performance [346]. Dendrites are needle‐like lithium structures that form during repeated charge and discharge cycles, particularly at the electrolyte/anode interface [347]. Their uncontrolled growth may extend into the electrolyte, resulting in internal short circuits, diminished cycle life, and even thermal runaway, heightening the risk of battery failure and potential safety hazards [348]. In conventional liquid electrolytes, lithium dendrites grow rapidly due to uneven ion deposition and localized current densities. While SSEs are designed to suppress dendrite formation by providing a mechanically rigid barrier, they are not entirely immune to this issue. Factors such as grain boundaries, interfacial instability, and insufficient ionic conductivity can still promote dendritic growth. Adding additional polymer to the initial solid polymer electrolyte has also been reported to be an effective way to prevent dendrite growth and has been shown to exhibit high mechanical strength (12 GPa) [346]. Zhao et al. [349]. A thorough investigation was carried out on three Li‐carboxylate COFs (denoted as LiOOC‐COFn, where n = 1, 2, 3) as single Li‐ion conductors. With well‐defined ion transport channels, the LiOOC‐COF3 conductor demonstrates impressive ion conductivity of 1.36 × 10^−5^ S cm^−1^ at ambient temperature and a high transference number of 0.91. It also displays exceptional electrochemical performance, including prolonged cycling stability, excellent capacity retention, and the prevention of dendrite growth in a quasi‐solid‐state organic battery. This battery employs cyclohexanehexone (C_6_O_6_) as the cathode and lithium metal as the anode, effectively mitigating organic electrode dissolution caused by the liquid electrolyte. In another study, Liu et al. [350] presented an electrospinning strategy to prepare scalable, self‐supporting COF membranes (COMs). These membranes feature a rigid COF skeleton bonded with flexible, lithiophilic (PEG) chains, forming an ion conduction network for Li^+^ transport. The resulting PEG‐COM electrolytes demonstrate improved dendrite suppression and an elevated ionic conductivity of 0.153 mS cm^−1^ at 30°C. Improved Li^+^ conduction in PEG‐COM electrolytes arises from loose ion pairing within the structure and an increased free Li^+^ content, as confirmed by solid‐state ^7^Li NMR experiments. These changes in the local microenvironment facilitate directional Li^+^ movement within the COM pores. Consequently, solid‐state symmetrical Li|Li, Li|LFP, and pouch cells demonstrate excellent electrochemical performance at 60°C. This strategy offers a universal method for developing scalable COM‐based electrolytes, expanding the practical applications of COFs in all‐solid‐state lithium metal energy storage devices.

Quasi‐solid‐state polymer electrolytes (QSSEs) represent a state between liquid and solid electrolytes, offering both high ionic conductivity at room temperature and substantial mechanical strength. These characteristics allow QSSEs to maintain stable operating conditions in batteries, making them highly suitable for practical use in solid‐state sodium batteries. COFs have attracted significant attention as polymer hosts for QSSEs in energy storage due to their well‐ordered one‐dimensional channels, tunable pore sizes, and a variety of functional groups [351]. Based on Na^+^/K^+^ ion conduction mechanisms observed in biological membranes, Yan et al. [352] reported a (–COO–)‐A modified COF was designed as a Na‐ion QSSE, featuring sub‐nanometer Na^+^ transport channels (6.7–11.6 Å) created by adjacent –COO– groups and COF walls, facilitating selective Na^+^ movement through electronegative regions. This QSSE achieves a Na^+^ conductivity of 1.30 × 10^−4^ S cm^−1^ and exhibits high oxidative stability up to 5.32 V (vs. Na^+^/Na) at 25 ± 1°C. In Na||Na_3_V_2_(PO_4_)_3_ coin cell tests, it shows fast reaction kinetics, low polarization voltage, and remarkable cycling stability for over 1000 cycles at 60 mA g^−1^, with only a slight capacity decay of 0.0048% per cycle and a final discharge capacity of 83.5 mAh g^−1^ at 25 ± 1°C. Wu et al. [353]. A novel class of single‐ion electrolytes was developed by synthesizing a series of crystalline COFs modified with sulfonimide groups. These COF‐based electrolytes create a directional pathway for lithium transport while providing numerous lithium‐ion binding sites, achieving an ionic conductivity of 4.3 × 10^−4^ S cm^−1^ at 298 K. Additionally, they exhibit a high lithium transference number (∼0.90) and a low activation energy of 0.24 eV. The study further highlights that electron‐withdrawing and delocalized substituents can enhance lithium‐ion transport. In Li||LiFePO_4_ (LFP) cells, the sulfonimide‐functionalized COF (SF‐COF) electrolytes demonstrate excellent rate performance and long‐term cycling stability, with 99% capacity retention after 100 cycles at 0.2C. These findings underscore the potential of crystalline COFs as essential components for solid electrolytes in lithium metal batteries.

These breakthroughs emphasize the substantial promise of COFs and polymer‐based electrolytes in addressing the challenges of solid‐state and quasi‐solid‐state batteries. By leveraging tailored ion transport pathways, structural flexibility, and interfacial stability, these materials exhibit promising electrochemical performance, including high ionic conductivity, long‐term cycling stability, and effective dendrite suppression. Future research should focus on optimizing polymer‐COF hybrid structures, improving mechanical integrity, and scaling up production for practical applications. Table 5 provides a comprehensive overview of recent research on advanced electrolytes and their electrochemical performance, offering insights into their feasibility for next‐generation energy storage systems.

**TABLE 5:** Summary of POPs battery materials, highlighting their working ions/parts, compound names, synthesis methods, surface areas, discharge capacities, and cycle stability.

Battery working ion‐part/supercapacitor	Name of compound	Synthesis method	Surface area (m^2^/g)	Discharge capacity (mAh/g)/capacity retention	Cycle stability	Refs.
Lithium‐ion battery Cathode	BFPPQ‐COF@CNT‐50	The Schiff reaction between TAPPy and BFPPQ facilitated the in situ growth of COF on CNTs.	296 (BFPPQ‐COF), 498 (COF@CNT‐25), 435 (COF@CNT‐50)	44.2 mAh/g (BFPPQ‐COF, 0.2C) 87.5 mAh/g (COF@CNT‐50, 0.2 C)	86% retention after 600 cycles at 5C (COF@CNT‐50)	[339]
Lithium‐ion battery Cathode	Poly (2,2,6,6 tetramethylpiperidinyloxy‐4‐yl methacrylate) and reduced GO‐PTMA/rGO composite	Dissolution‐assembly‐depositing method	Not‐Mentioned	153 mAh/g at 20 mA/g	Exceptional rate capability, outstanding utilization efficiency (98.8%)	[366]
Lithium‐ion battery Anode and Cathode	HcPPy (Hyper‐crosslinked Pyrene‐based POP)	One‐step Friedel–Crafts reaction from pyrene	723	683 mAh/g at 1000 mA/g (Anode), 110 mAh/g at 100 mA/g (Cathode)	200 cycles (Cathode), excellent rate performance and long‐term stability	[367]
Lithium‐ion battery Cathode	TPPDA‐PICOF	Imide polymerization of TPPDA and PMDA in Nmethylpyrrolidone/mesitylene/isoquinoline	342	Initial: 40 mAh/g, after 200 cycles: 98% retention	After 200 cycles, 98% retention	[368]
Lithium‐ion battery Cathode	COF‐TRO	Condensation reaction between truxenone (TRO) and 1,3,5‐triformylphloroglucinol (TFP) under solvothermal conditions	156	268 mAh/g at 0.1C	99.9% capacity maintenance after 100 cycles at 0.1C	[369]
Lithium‐ion battery Cathode	COFTPDA‐PMDA@50%CNT	Water‐assisted polyimide synthesis with solvent mixture at 200°C for 5 days	2669	233 mAh/g at 0.5 A/g; 80 mAh/g at 5.0 A/g after 1800 charge–discharge cycles.	80 mAh/g at 5.0 A/g after 1800 cycles	[370]
Lithium‐ion battery Anode	SPP (Salen‐based Porous Framework Polymer)	Schiff‐base polymerization between DAB and HBC	89.3	337 mAh/g at 0.1C after 100 cycles	Consistent performance with 155.7 mAh/g at 8C after 4000 charge–discharge cycles.	[371]
Lithium‐ion battery Cathode	LFP with SP	Mechanofusion method, carbon coating	Not‐Mentioned	165.6 mAh/g (at 0.1C), 59.8 mAh/g (at 10C)	The charge/discharge test demonstrates a capacity of 59.8 mAh/g at a 10C rate.	[372]
Lithium‐ion battery Cathode Material	Dual‐porous COF / USTB‐6	Polymerization of hexa(4‐formylphenyl) diquinoxalino[2,3‐a:2′,3′‐c]. phenazine and 2,7‐diaminopyrene‐4,5,9,10‐tetraone in the presence of graphene	328 (for USTB‐6), 384 (for USTB‐6@G)	285 at 0.2C, 188 at 10C	All the LFP/C‐SP electrodes exhibit better capacity retention compared to LFP/C, attributed to the SP coating on LFP/C‐S.	[373]
Potassium‐Ion Batteries Cathode	Polyimide@Ketjenblack (PIM@KB)	In situ polymerization	82.6 (PIM@KB), 18.7 (PIM)	143 mAh/g at 100 mA/g, 125 mAh/g at 1000 mA/g, 105 mAh/g at 5000 mA/g	A capacity retention of 76% was observed after 1000 cycles at a current density of 2000 mA/g.	[374]
Lithium‐ion battery Anode and Cathode	POP‐1, POP‐2, POP‐3	Azo coupling reaction of phloroglucinol and diarylamines	POP‐1: 294, POP‐2: 374, POP‐3: 5	POP‐1: 441.6 mAh/g (first cycle), POP‐2: 187.3 mAh/g (second cycle), POP‐3: 338.7 mAh/g	POP‐1: 74% (1000 cycles), POP‐2: 91.0 mAh/g (1000 cycles), POP‐3: 79% (1000 cycles)	[375]
Lithium‐ion battery Cathode	Py‐A‐CMP, TPE‐A‐CMP	Synthesis via Pd‐catalyzed Sonogashira coupling reaction, deprotected by K_2_CO_3_	123	196.6 (0.1C)	Maintained 140 mAh/g after 50 cycles, 120 mAh/g after 400 cycles	[335]
Lithium‐ion battery Anode and Cathode	DAAQ‐COF	Schiff‐base reaction via solvothermal method	73.1 m^2^/g	787 mAh/g after 500 cycles at 1 A g^−1^	High cycle stability with "activation" behavior; capacity increases after 50 cycles; enhanced ionic diffusion and redox processes during cycling.	[376]
Lithium‐ion battery Anode and Cathode	pBHT, pBAT, pBABT	FeCl_3_‐catalyzed oxidative coupling (pBHT); Pd‐catalyzed cross‐coupling (pBAT, pBABT)	1139 (pBHT), 32.5 (pBAT), 12.5 (pBABT)	<100 (pBHT), 442 (pBABT, second cycle, 500 mA/g)	401 mAh/g after 1000 cycles (pBABT, 500 mA/g)	[337]
Sodium‐Ion Batteries Cathode	Exfoliated NDI‐TFP Polymer	Modified solvothermal with exfoliation using methanesulfonic acid (MSA)	64 (after exfoliation), 230 (before exfoliation)	92 mAh/g (initial), 82 mAh/g (after 100 cycles at 20 mA/g)	98% Coulombic efficiency; retains 82 mAh/g after 100 cycles at 20 mA/g	[377]
Sodium‐ion batteries Cathode	Aza‐COF	Condensation reaction of hexaketocyclohexane and 1,2,4,5‐benzenetetramine using H_2_SO_4_ catalyst	240	523	87% retention after 500 cycles	[378]
Sodium‐ion batteries Anode and Cathode	PICOF‐1	Linker exchange with imine‐linked COF as template	924	230	A Coulombic efficiency of 99% was maintained over 175 cycles at a rate of 0.3C.	[379]
Sodium‐ion batteries Cathode	Conjugated porous polyimide poly(2,6‐diaminoanthraquinone) benzamide (CP‐PDAB)	One‐step polycondensation	337	100	200 cycles, more stable capacity	[380]
Sodium‐ion battery Cathode	Nanostructured K1–xFe_0.5_Cu_0.5_ O_2_@CNTs	Hydrothermal method	580 for PIA, 760 for PIB, 990 for PIC, and 1430 for PID	221.2 mAh/g (0.1C)	Retains 82.5% capacity after 1000 cycles at 1C	[381]
Sodium‐ion batteries Cathode	NaFePO4@rGO composites	Solvothermal method	389.8	82.5 mAh/g at 0.1C	Retained 70% of the initial capacity after 100 cycles.	[382]
LIBs Electrolytes	C‐COF@ (Tetra(p‐amino‐phenyl) porphyrin (TAPP, C_44_H_34_N_8_) with C‐based linkers) N‐COF@ (Tetra(p‐amino‐phenyl) porphyrin (TAPP, C_44_H_34_N_8_) with N‐based linkers), F‐COF@(Tetra(p‐amino‐phenyl) porphyrin (TAPP, C_44_H_34_N_8_) with F‐based linkers)	C‐COF, N‐COF, and F‐COF were synthesized by solvothermal condensation of TAPP and linkers, followed by heating, filtration, and vacuum drying.	C‐COF: 497, N‐COF: 196, F‐COF: 147	115 mAh/g at 1C after 100 cycles	90.8% for 300 cycles at 5C	[383]
LIBs Electrolytes	EB‐COF‐TFSI, PEO (C_2_H_4_O) n, LiTFSI (LiN(SO_2_CF_3_) _2_), Nano Al_2_O_3_	EB‐COF‐TFSI is synthesized by reacting EB and TFP in mesitylene/dioxane, followed by treatment with LiTFSI. For SSE, PEO, LiTFSI, Nano Al_2_O_3_, and EB‐COF‐TFSI are mixed, dried, and pressed into a membrane	748.7	167.9 mAh/g at the 263rd cycle	167.9 with a nearly 100 % coulombic efficiency even at the 263rd cycle	[384]
Supercapacitors	DAAQ‐COFs/graphene composite aerogel	GCF was made by carbonizing GO‐coated MF at 800°C. DAB‐COF was synthesized by reacting DHA and TAB at 200°C for 3 days. DAB/GCF was formed by polymerizing monomers on GCF at 200°C.	1174.2	Capacitance retention 100% @ 20 000 cycles	In addition, the DAB/GCF‐based SSC exhibits exceptional cycling durability, maintaining almost full capacitance retention after 20 000 charge–discharge cycles at a current density of 10 A/g.	[365]
Supercapacitors	COF/carbon composite foam (DHA‐TAB COF on GCF from MF).	DAAQ‐COFs were synthesized via solvothermal methods and modified with CTAB, then electrostatically assembled with GO nanosheets to form the DAAQ‐COFs/graphene composite aerogel.	1017.9	Capacitance retention 88.9% @ 20 000 cycles	The fabricated ASC demonstrates outstanding cycling endurance, retaining 88.9% of its capacitance after 20 000 charge/discharge cycles at a current density of 5 A/g.	[363]
Supercapacitors	DBTh‐TPA and DBTh‐TPT CMPs	DBTh‐2Br was synthesized by dimerization with n‐BuLi in tetrahydrofuran, and DBTh‐TPA and DBTh‐TPT CMPs were prepared using Suzuki coupling in DMF at 150°C.	DBTh‐TPA:551, DBTh‐TPT:376	Capacitance retention of DBTh‐TPA 80% @ 10 000 cycles, Capacitance retention of DBTh‐TPT 85% @ 10 000 cycles	Following 10 000 cycles, the symmetric device retained 73.2% of its initial capacitance.	[360]
Supercapacitors	Py‐BDT and Py‐Ph‐BDT CMPs	Py‐BDT and Py‐Ph‐BDT CMPs were synthesized via Suzuki–Miyaura coupling with yields of 80% and 81%, respectively.	Py‐BDT:208, Py‐Ph‐BDT CMPs:427	Not‐Mentioned	Cycling durability assessments revealed that 72.87% of the original capacitance was preserved after 5000 cycles.	[385]
Supercapacitors	PNOC‐800‐3@NiS‐a‐T	NiS‐based composites were hydrothermally synthesized, optimizing nickel‐sulfur ratios and temperatures for enhanced electrochemical performance in ASC's.	1006.31	Capacitance retention of PNOC‐800‐3@NiS‐2‐180 72.63% @ 5000 cycles	The ASC maintains 72.63% of its capacitance after 5000 cycles at 1 A g^−^^1^, showcasing outstanding stability.	[386]
Supercapacitors	Sulfonated hyper‐crosslinked polymer	SHCPs were synthesized via a one‐pot reaction by dissolving 4,4‘‐bis(chloromethyl)‐1,1‘‐biphenyl in DCE, cooling, adding chlorosulfonic acid, refluxing at 80°C for 22 h, followed by methanol washing and vacuum drying.	715 ± 32	The capacitance retention was approximately 50% of the initial value after 483 cycles.	while cycling between ±0.5 V at 21°C and 58% RH retained 96% capacitance after 3500 cycles	[387]

#### 5.3.3 POPs as Electrode in Supercapacitors

SCs commonly referred to as ultracapacitors, provide a unique balance between batteries and traditional dielectric capacitors, providing high power and energy densities [354]. Their ability to deliver rapid charge and discharge cycles makes them ideal for applications such as electric vehicle acceleration, emergency door mechanisms in aircraft, uninterruptible power supplies, airbag deployment, pacemakers, and gantry cranes [355, 356].

POPs have attracted interest for their adjustable pore structures, low density, large surface areas, and strong chemical stability [11]. In research by Basit et al. [357]. A supercapacitor electrode was developed by synthesizing an octavinyl silsesquioxane (OVS)‐fluorenone‐based polymer (OVS‐FO‐POIP) through a Heck coupling reaction between OVS and 2,7‐dibromo‐9H‐fluoren‐9‐one (DBFO). The chemical structure of OVS‐FO‐POIP was confirmed using solid‐state 13C and 29Si NMR, alongside Fourier transform infrared spectroscopy. Following carbonization and KOH activation at 900°C, the resulting porous carbon material (OVS‐FO‐POIP‐900) exhibited a substantial enhancement in specific capacitance, reaching 776 F g^−1^ at 1 A g^−1^, compared to 271 F g^−1^ for the unmodified polymer. Additionally, OVS‐FO‐POIP‐900 showed exceptional cycling stability, retaining 79% of its capacitance after 6000 cycles.

Despite their advantages (e.g. high surface area, tunable porosity, chemical stability, and lightweight nature), CMPs and COFs often suffer from low electronic conductivity and limited cycling durability, restricting their practical applications, CMPs and COFs often suffer from low electronic conductivity and limited cycling durability, restricting their practical applications [358, 359]. To overcome these challenges, conductive additives, pyrolysis techniques, and redox‐active moieties have been incorporated into CMP structures. One approach involved the synthesis of two redox‐active benzo[1,2‐b:4,5‐b’]. dithiophene‐4,8‐dione‐based CMPs, namely DBTh‐TPA and DBTh‐TPT, through a one‐pot Suzuki coupling polymerization [360]. These materials displayed exceptional thermal stability (T_d10 ≈ 608.4°C) and high surface areas (∼551 m^2^/g). The incorporation of redox‐active DBTh structures improved conductivity, charge transfer, and faradaic energy storage. Noteworthy, DBTh‐CMPs demonstrated an exceptional three‐electrode specific capacitance of 1823 F/g at 0.5 A/g, along with impressive cycling stability, retaining 81.75% of capacitance after 10 000 cycles—the highest value reported for CMPs to date. Additionally, a two‐electrode symmetric supercapacitor device incorporating DBTh‐TPT CMP achieved a specific capacitance of 609 F/g and a peak energy density of 84.5 Wh/kg at a power density of 500 W/kg, operating at 1.0 V.

Hybrid electrodes combining COFs with conductive materials have been explored to address electron transfer limitations and enhance the effective utilization of active sites [361, 362]. A recent investigation presented the development of an anthraquinone‐based COF/graphene composite aerogel (DAAQ‐COF/GA) electrode, formed by electrostatic assembly of negatively charged GO nanosheets with positively charged nanoflower DAAQ‐COFs [363]. This 3D crosslinked conductive network exhibited a notable specific capacitance of 378 F g^−1^ at 1 A g^−1^ and a rapid charge storage mechanism, as evidenced by a 93.4% capacitive contribution at 3 mV s^−1^. In an asymmetric supercapacitor (ASC), the binder‐free DAAQ‐COF/GA electrode demonstrated an energy density of 30.5 Wh kg^−1^ at a power density of 700 W kg^−1^.

Developing large‐scale, self‐sustaining 3D COF‐based materials with mechanical strength is crucial for their application in wearable energy storage devices [364]. One method involves directly growing COF crystallites on a carbon network through a Schiff‐base reaction, resulting in a COF/carbon composite foam with a highly interconnected structure [365]. This architecture promoted efficient electron transfer, outstanding mechanical characteristics (including high compressibility and resistance to fatigue after 50 cycles at 70% strain), and improved access to active sites. Serving as a self‐supporting electrode for SCs, the COF/carbon composite foam achieved a specific capacitance of 129.2 F g^−1^ at 0.5 A g^−1^ and demonstrated exceptional cycling durability, retaining nearly 100% of its capacitance after 20 000 cycles at 10 A g^−1^. Its flexibility and durability allowed it to retain capacitive performance even under extreme bending and compression, positioning it as a promising material for future flexible and wearable SCs. These advancements, particularly the demonstrated flexibility, durability, and cycling stability under extreme bending and compression, highlight the significant potential of POPs in advancing the development of high‐performance supercapacitor electrode materials. By leveraging their tunable porosity, high surface area, and structural versatility, electrodes have been engineered to exhibit remarkable capacitance, improved charge storage capabilities, and enhanced cycling stability. The incorporation of redox‐active units and conductive additives has further improved electron transport, overcoming the intrinsic limitations of traditional porous frameworks. The ability of POP‐based materials to form flexible, free‐standing architectures also positions them as key candidates for advanced wearable energy storage applications. Moving forward, the strategic design and functionalization of POPs will be instrumental in bridging the performance gap between batteries and capacitors, driving the evolution of high‐energy‐density SCs with superior mechanical and electrochemical properties.

Advancements in POPs have improved supercapacitor performance by enhancing capacitance, cycling stability, and electron transport. Functionalized POPs, including OVS‐based materials and redox‐active CMPs, offer superior thermal stability and charge storage. Integrating COFs with graphene aerogels and carbon composite foams has further increased conductivity and mechanical durability [139]. These innovations position POP‐based materials as promising candidates for flexible, high‐energy‐density SCs.

### 5.4 POPs in Energy Harvesting

Wireless energy‐responsive platforms provide a crucial foundation for advancing intelligent systems. However, the complexity of modern electronic components creates challenges for their seamless deployment in real‐world environments. This limitation hinders the efficient integration of essential functions such as energy harvesting, storage, sensing, and communication, emphasizing the need for innovative solutions to enhance their adaptability and performance [388].

To overcome these issues, the development of electronic devices has increasingly prioritized miniaturization, multifunctionality, portability, flexibility, high computational efficiency, and low‐power communication [389]. Harnessing environmental energy sources, including mechanical vibrations, heat, fluid movement, electromagnetic radiation (such as light and radio waves), and biological energy, offers a renewable approach to powering diverse applications. These span from wireless sensor systems to portable electronics, along with wearable or implantable medical devices [390].

**FIGURE 21:**
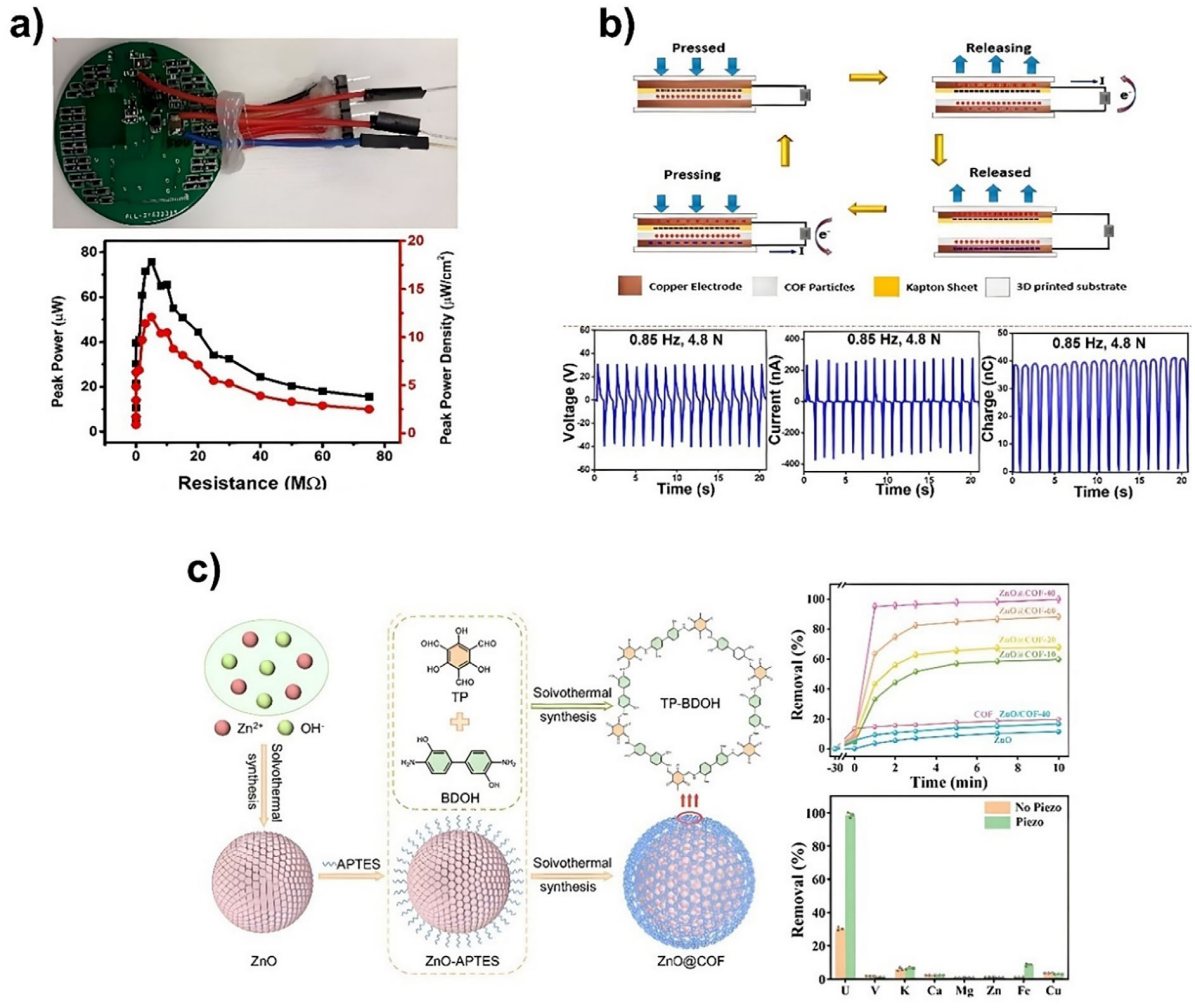
(a) Electrical and electrochemical characterization of a copper‐based electrode system. (Top Left) Photograph of a printed circuit board (PCB) with connected wires, showcasing the experimental setup. (Bottom Left) Graph plotting peak voltage (V) against resistance (MΩ), with a sharp decline followed by stabilization, indicating the electrode's response under applied conditions. (Right) Schematic representation of the electrochemical setup, highlighting current density and cycling behavior. Reproduced with permission from ref. [395]. Copyright 2020, American Chemical Society. (b) Fabrication process and voltage profiles of a layered electrode material. (Top) Schematic illustrating the pressing and releasing steps of the electrode preparation process, with annotations for Cu electrode, Ni foam, and Cu/CuO layers (scale bars: 20 µm). (Bottom) Voltage‐time profiles during charge–discharge cycles, showing stable voltage oscillations at 0.8 V and 0.4 V, indicative of reversible electrochemical reactions over time (Time (s)). Reproduced with permission from ref. [394]. Copyright 2022, Elsevier. (c) Synthesis and structural analysis of zinc‐based nanomaterials. (Top Left) Schematic of the solvothermal synthesis process, depicting the transformation of Zn^2+^/OH^−^ precursors into ZO‐APTES and ZO‐COF structures via TPBDAH and solvothermal conditions. (Top Right) SEM images of spherical ZO‐APTES and porous ZO‐COF morphologies (scale bars: 1 µm). (Bottom) Graph plotting particle size or crystallinity against reaction time (hours), comparing ZO‐APTES and ZO‐COF with reference materials (e.g., ZnO), illustrating growth kinetics and structural evolution. Reproduced with permission from ref. [397]. Copyright 2021, Nature.

Batteries commonly used in portable electronic devices face inherent limitations, including environmental concerns related to disposal, restricted lifespan, and accessibility challenges [391]. To address these drawbacks, nanogenerators have been explored as a promising alternative for sustainable energy harvesting, enabling the conversion of irregular mechanical energy into electrical power suitable for driving micro‐ and nano‐watt electronics [392, 393]. Given the widespread presence of mechanical vibrations in natural and engineered environments, various nanogenerator technologies have been developed to efficiently convert mechanical energy into electrical output. These systems leverage distinct transduction mechanisms, including piezoelectric, triboelectric, pyroelectric, and electromagnetic effects, to enhance energy conversion efficiency and broaden their applicability [394]. Masud et al. [395] introduce an innovative method to enhance structural health monitoring (SHM) in aerospace by creating a self‐powered wireless sensing system utilizing a Polyvinylidene Fluoride (PVDF‐based) piezoelectric nanogenerator (PENG). Their research investigates the combination of flexible PVDF with zinc oxide (ZnO) nanoparticles, resulting in a substantial improvement in output performance, leading to an 11‐fold increase in current and an 8‐fold rise in voltage compared to pure PVDF devices. The device produces a peak power density of 41.02 µW/cm^2^, enabling wireless data transmission every 4 min by harvesting energy from small vibrations (Figure 21a). This work explores the potential of porous PVDF‐based Piezoelectric nanogenerators (PENGs) as power sources for SHM sensor networks in aircraft.

Piezoelectric catalysis has garnered considerable attention as an effective method for converting mechanical energy into chemical energy. This process is triggered by external mechanical vibrations that induce polarization, resulting in the generation of a piezoelectric potential and the creation of an internal electric field. This mechanism not only facilitates the generation of active free radicals but also improves charge separation, decreasing the dependence on sacrificial agents. Among the various piezoelectric materials, ZnO has garnered significant attention due to its distinctive properties, which stem from its non‐centrosymmetric fibrillar zincite structure. However, ZnO faces notable challenges, including its wide bandgap and high resistance to electron transfer, both of which hinder its performance. To enhance the piezoelectric efficiency of ZnO, researchers have explored creating heterojunctions by combining ZnO with materials that have compatible band structures, such as CuI/ZnO, FTC‐ZnO, and GQDs/ZnO. However, these combinations often exhibit limited tunability and instability, which constrains their practical applications [396]. Jia‐Xin et al. fabricated ZnO@COF core–shell hybrids through the in situ deposition of COFs onto the aminated surfaces of ZnO. This design utilizes the ZnO core for improved piezoelectric performance, while the COF shell enhances electron transport and catalytic activity. The ZnO‐COF junction creates an interfacial electric field, minimizing metal edge shielding and promoting efficient charge separation, leading to superior piezoelectric properties compared to conventional materials. The hybrid material exhibited high efficiency in uranium extraction from seawater, achieving a rate of 7.55 mg g^−1^ d^−1^ under wave‐driven conditions as shown in Figure 21c. This study investigates the use of ZnO@COF composites for piezocatalytic uranium extraction from seawater, utilizing mechanical energy from ocean waves. It also examines the material's performance in energy harvesting and environmental applications [397].

After exploring the mechanisms of converting mechanical energy into electricity through piezoelectricity and piezocatalysis, both of which require mechanical activation, it is natural to transition toward triboelectricity. Similar to piezoelectricity, triboelectricity is a physical phenomenon that also converts mechanical energy into electrical energy. However, it differs significantly in terms of operational principles and the materials used. In piezoelectricity, electrical charge is generated through changes in the crystal structure of materials, whereas in triboelectricity, electrical charges are transferred through friction between two distinct surfaces. This fundamental difference offers unique opportunities for energy harvesting and various other applications, setting triboelectricity apart from its counterparts in terms of material requirements, efficiency, and versatility [398]. The discussion effectively introduces triboelectricity while clearly highlighting its distinctions from piezoelectricity, creating a smooth and engaging transition for a review. Subsequent sections can further explore triboelectric applications and ongoing research [399].

POPs are instrumental in advancing the performance of triboelectric nanogenerators (TENGs), which convert mechanical energy from environmental sources like body movements and wind into electrical power [400]. With their high surface area, tunable porosity, and structural versatility, POPs serve as effective triboelectric layers or substrates, enhancing charge generation and retention by maximizing contact area and improving charge trapping efficiency [401]. TENGs exhibit diverse operational mechanisms and offer several benefits, including cost‐effectiveness, minimal weight, extensive material compatibility, and high energy conversion efficiency, even at low operational frequencies. The effectiveness of TENGs is largely influenced by the selection of materials positioned at opposite ends of the triboelectric series, as their distinct charge affinities contribute to optimizing device [402]. This allows for the extraction of micromechanical energy, positioning it as a sustainable and eco‐friendly energy source alongside other renewable sources like solar, wind, geothermal, and tidal power. TENGs are preferred due to their straightforward design, affordability, flexibility, high‐energy output, safety, small form factor, and simple manufacturing process. The extensive range of materials available for TENG components further enhances their appeal for energy storage applications [403]. Despite these advantages, challenges persist in improving current densities and output potentials for practical use. The performance of TENGs is influenced by factors like friction during motion, environmental conditions, and the specific properties of the triboelectric materials. Although a broad range of inorganic and organic materials can be employed in TENG construction, achieving continuous and efficient energy conversion through the deliberate molecular design of these materials remains a substantial challenge [404]. Recent advancements in energy harvesting technologies have established TENGs as a viable and environmentally friendly solution for transforming mechanical energy into electrical power. These devices exploit triboelectric effects, where mechanical movement induces an electrical charge through the interaction and detachment of materials [405]. Huijie et al. developed a metalated (Cu‐COF) using a post‐metalation strategy, incorporating transition metal ions into a CF_3_‐substituted COF to enhance TENG performance. The Cu‐COF demonstrated exceptional triboelectric characteristics, achieving a peak short‐circuit current of 104.0 µA and an output voltage of 1107 V. Compared to CF_3_‐COF, the Cu‐COF outperformed it by factors of 1.47 and 1.39, respectively. This improvement is ascribed to increased charge density, enhanced conductivity, and a reduced band gap., optimizing charge generation and device stability. The Cu‐COF‐based TENG demonstrated high efficiency, successfully powering 1440 commercial LEDs, underscoring its potential for sustainable energy harvesting. This study highlights metalation as a powerful tool for COF engineering, paving the way for next‐generation TENG materials with enhanced performance and scalability. Hajra et al. investigated the potential application of COFs in TENGs by synthesizing a triazine‐based nitrogen‐rich COF and evaluating its triboelectric properties. Using Kelvin probe force microscopy, they measured a notable positive surface potential of 2.03 V, representing a significant step forward in COF‐based TENG research. A single COF‐based TENG unit produced an electrical output of 70 V, 0.6 µA, and 38 nC, as illustrated in Figure 21b, when four units were connected in parallel, the peak‐to‐peak current increased to 6.3 µA, while a series connection resulted in a high peak‐to‐peak voltage of 175 V. This study highlights the potential of N‐rich COFs for efficient, cost‐effective energy harvesting, especially in self‐powered hand‐strengthening devices [394].

Low‐grade heat (below 100°C) from industrial and natural sources accounts for over 30% of global primary energy consumption, with approximately 85 PWh/year lost due to inefficient utilization [406, 407]. Harnessing this heat can improve energy efficiency and reduce carbon emissions, making it a promising solution to the global energy crisis. Advances in ionic thermoelectric systems, such as thermo‐ionic capacitors and thermo‐galvanic cells, facilitate the conversion of thermal energy into electrical power [408, 409]. Permselective membranes drive ion movement along temperature gradients, generating an electric flux that is collected by electrodes [410]. The efficiency of this thermoelectric conversion is primarily determined by the membrane's permselectivity, particularly in high‐salinity environments, where maintaining optimal ionic conductivity becomes a significant challenge. Among the various materials being explored for their ionic conductivity, electrically charged COF membranes have emerged as a promising solution. Their tightly linked nanopore structures enable superior ionic conductivity and selectivity by enhancing ion transport and screening [411]. The unique structure of COFs not only supports high ionic conductivity but also allows for the efficient separation and transport of charged particles, making them ideal for application in ionic thermo electrics. Further research into optimizing these COF membranes could potentially lead to significant improvements in the performance of thermoelectric systems designed for harvesting low‐grade heat. Yin et al. investigated the potential of COF membranes to transform low‐temperature waste heat into electricity by modifying their linkages with β‐ketoenamine bonds to enhance ion‐membrane interactions and electrostatic potential distribution. This resulted in a charge‐neutral COF membrane that maintains strong permselectivity, even under high‐salinity conditions. Experimental tests with a 3 m KCl solution and a 50 K temperature gradient yielded a notable power density of 5.70 W m^−2^, underscoring its efficiency in thermoelectric energy conversion. The study also introduced a localized ionic screening process that allows adjustable permselectivity through controlled ion adsorption, optimizing ion transport pathways. These findings establish COF membranes as a promising material for sustainable energy harvesting, emphasizing their potential for scalable and stable integration into real‐world thermoelectric applications [412].

### 5.5 Biomedical Applications

POPs have made significant strides in drug delivery, bioimaging, and biosensing; yet, a number of significant obstacles still stand in the way of their effective clinical translation. Their long‐term biological destiny and degradability are among the most significant issues. Many POPs have highly crosslinked and chemically stable frameworks that offer superior structural integrity during biomedical applications; however, this stability may also lead to slow degradation, extended tissue retention, and possible accumulation in reticuloendothelial system organs, especially the spleen and liver. There is still few thorough research on in vivo metabolic routes, breakdown products, long‐term biodistribution, and excretion processes, despite a number of studies showing low acute toxicity and favourable short‐term biocompatibility [36].

Another major challenge is the insufficient understanding of the immunological responses elicited by POP‐based nanomaterials. When nanoparticles are repeatedly administered, their interactions with proteins, immune cells, and biological barriers may cause complement activation, inflammatory reactions, or faster clearance. Moreover, there are presently few chronic toxicity studies assessing long‐term organ accumulation, repeated‐dose exposure, and possible negative effects of breakdown byproducts. It is challenging to provide the standardized safety profiles needed for clinical implementation and regulatory approval because of these information gaps [36, 58].

The creation of biodegradable and bioresponsive POP platforms has received more attention in an attempt to get around these restrictions. Under physiological or disease‐specific circumstances, the addition of cleavable linkages, such as imine, hydrazone, disulfide, ester, and azo bonds, might expedite framework disintegration and eventual removal from the body. Furthermore, surface engineering techniques such as PEGylation, zwitterionic modification, and biomimetic membrane coating have demonstrated significant potential in improving circulatory behaviour, decreasing nonspecific protein adsorption, increasing colloidal stability, and diminishing immune recognition. Particle size, shape, and surface charge optimization may enhance cellular absorption and facilitate hepatobiliary or renal clearance [36, 413, 414].

#### 5.5.1 Drug Delivery

One of the foremost uses of POPs, a perennial focus of research, is their deployment as smart drug‐delivery vehicles. Properties such as unrivaled molecular precision, architected porosity, and engineered resilience aid the therapeutic encapsulation and release. These supramolecular scaffolds transcend traditional carriers by offering programmable host‐guest interactions, enabling spatiotemporal control over drug liberation [415]. A defining trait that has emerged as a research priority is the inherently robust frameworks of POPs, which resists biological degradation under harsh conditions such as gastrointestinal (GI) conditions, while maintaining structural integrity, ensuring zero residual toxicity—a critical advantage over metabolically labile alternatives (e.g., capsules, liposomes, MOFs). For instance, Enpeng Xi et al., produced PAF‐1 loaded with Famotidine (Famo) for gastric disorders and Mesalazine (Mesa) for ulcerative colitis as an oral drug delivery carrier [416]. The system achieved multi‐organ drug release without degradation and was fully excreted within 12 h without leaving any toxic residual nanomaterials in vivo. The PAF‐1 was functionalized with amino groups through treating with concentrated HNO_3_ and further reduction to PAF‐1‐NH_2_ (PNH) to create anchor points for covalent conjunction of both drugs. Dual‐drug loading (PFM) enables stomach‐intestine compartmentalized release without cross‐interference, where Famo was linked via the amide bond formation between Famo's carboxyl group and PNH's –NH_2_, (which hydrolyzes in gastric acid (pH 1.2)) and enzyme‐sensitive azo bond (–N = N–) formation between Mesa and PNH's –NH_2_, (which cleaves via azoreductase enzyme in the intestine). Moreover, clinical test on mice, were conducted to investigate the efficiency of the PFM nanocarrier further. The experiment results indicate the group who received PFM nanocarrier showed restored colon length, reduced diarrhea, and fecal blood, despite the disease control group, implying the efficiency of this novel system. In order to direct the drugs to the exact spot, decrease its accumulation in healthy organs, maximize drug release while minimizing the side effects, a dual stimuli‐responsive system, named as TA‐COF‐P@CT with AZO groups within its structure, was prepared, which combined the advantages of photodynamic therapy and chemotherapy at the same time.^3^ In tumor microenvironments, the low concentration of oxygen (a condition known as hypoxia) triggers overproduction of enzymes (reductases), causing cleavage of AZO bonds and fragmentation of TA‐COF‐P@CT, leading to the release of Chlorin e6 (Ce6) and hypoxia‐activated prodrug tirapazamine (TPZ). The second step of the drug delivery mechanism, is followed by laser exposure of tumor cells, where Ce6 generates ^1^O_2_ (singlet oxygen) and exacerbates the tumor hypoxia, leading to acceleration of COF breakdown and more TPZ/Ce6 liberation. The in vitro applicability of this nanocargo was confirmed by the size redistribution of particles from unimodal to trimodal when sodium hydrosulfite (Na_2_S_2_O_4_) was utilized as a well‐established chemical surrogate for azo reductase, revealing the deintegration of the COF structure.

In another group effort leaded by Yu Zhao, a novel 3D COF with stp (stitched triptycene‐porphyrin) topology named as TUS‐64 with record‐breaking pore size of 4.7 nm and ultra‐low density (0.106 g/cm^3^) was produced [417]. The synthesis of this ultralight COF relies on two building blocks, including a D_3h_‐symmetric triangular prism node and a C_4_‐symmetric square planar linker via a Schiff‐base condensation. The enormous pore size of the TUS‐64 sorts it in the class of suitable carriers for different therapeutic agents, which in this case the high surface area of 1632 m^2^/g faced a tremendous decreased after encapsulation by Captopril, Ibuprofen, Isoniazid, 5‐Fluorouracil and Brimonidine to 78.4 m^2^/g (95.2% reduction), 869.3 m^2^/g (46.7% reduction), 610.0 m^2^/g (62.6% reduction), 378.7 m^2^/g (76.8% reduction) and 501.8 m^2^/g (69.3% reduction) respectively. Notably, the retention of crystallinity (via PXRD) and unchanged morphology (via SEM) further corroborate that drug molecules are confined within the pores rather than adsorbed superficially. The release profiles of drugs indicated that captopril releases quickly fast (86% in 12 h, 92% in 2 days) while isoniazid has the slowest releasing (16% in 24 h, 22% in 10 days) comparing to all of them. Not only in the drug delivery, but also in gene delivery, POPs have proven to be very useful as promising non‐viral vectors, as demonstrated by Yaqi Cao et al., in a study where functionalized CTF nanosheets achieved superior transfection efficiency and lower cytotoxicity. After exfoliating layered CTF into 2D nanosheets (∼8 nm thick), the original −NO_2_ groups on the surface were converted to −COOH to prepare it for subsequent PEGylation and grafting of PEI polymer. The resulted CTF‐PEG‐PEI with a brush‐like hierarchical structure (thickness: 20–26 nm) has superior gene transfection efficiency in 293T and HeLa cells comparing to pure PEI at low doses (0.5–1 µg) and lower toxicity. Modifying the carrier with PEG helps to enhance colloidal stability in biological media, while easier endosomal escape and balanced DNA binding are the advantages of PEI conjunction [418].

#### 5.5.2 Bioimaging

**FIGURE 22:**
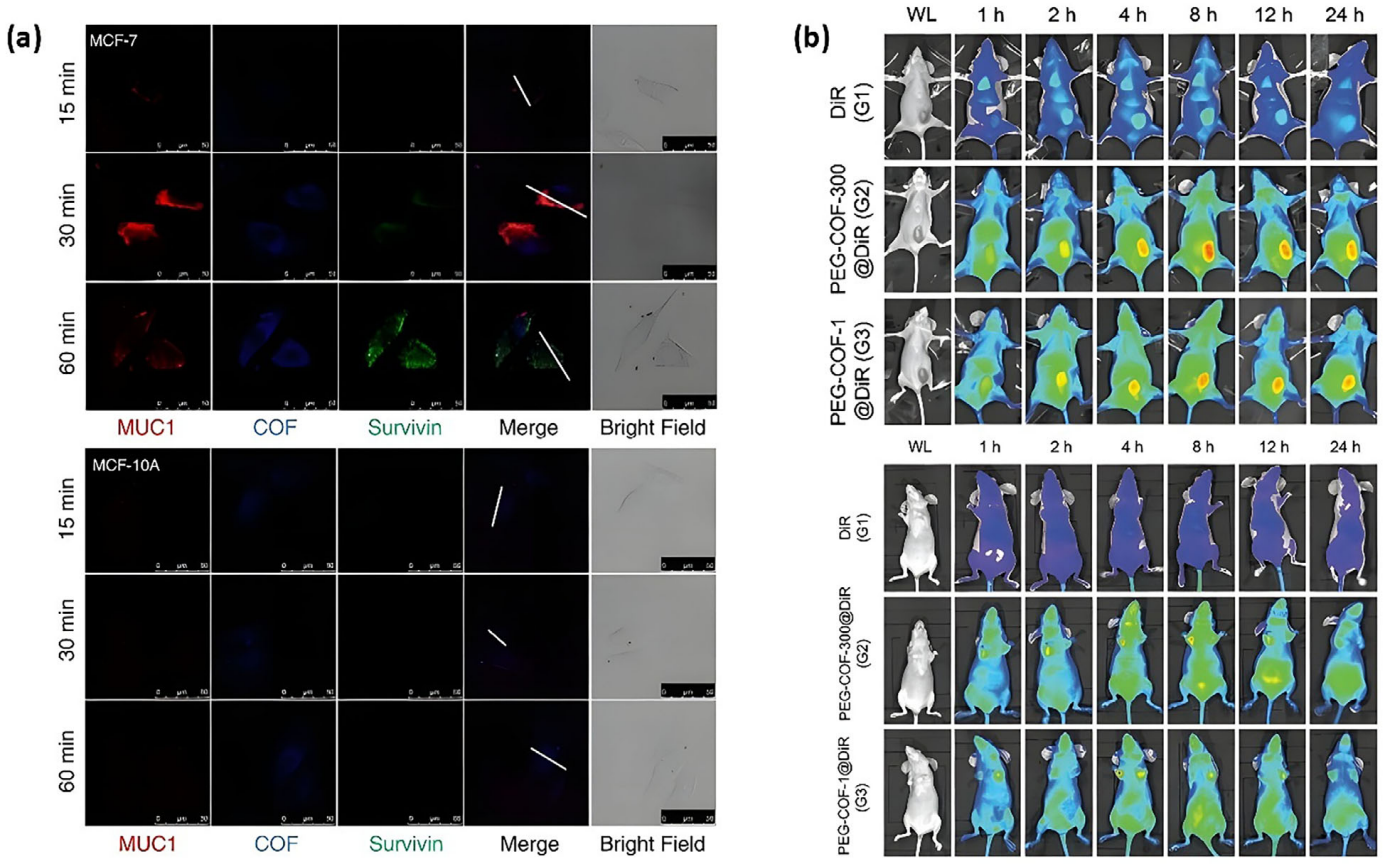
(a) Confocal imaging of cancer and normal cells with the nanoprobe. Confocal laser scanning microscopy (CLSM) images of MCF‐7 (top) and MCF‐10A (bottom) incubated with the nanoprobe for different times. Reproduced with permission from ref. [421]. Copyright 2021, American Chemical Society. (b) in vivo fluorescence imaging of the tumor‐bearing mice at 1, 2, 4, 8, 12, and 24 h (top) and healthy mice(bottom) following injection with PEG‐COF‐300@DiR and PEG‐COF‐1@DiR. Reproduced with permission from ref. [422]. Copyright 2024, Wiley‐VCH.

The fluorescence emission of POPs, much like other optical characteristics, can be precisely tuned to specific spectral ranges to apply them as potential imaging probes in the field of diagnosis. Their hierarchical nanostructuring enables precise control over optical properties, allowing for high‐contrast imaging in deep tissues while resisting photobleaching, which is counted as a critical advantage over organic dyes. By integrating π‐conjugated fluorophores (e.g., pyrene, porphyrins) into their rigid frameworks, POPs achieve intrinsic, aggregation‐resistant emission, where the need for external labels will be eliminated. COFs are attracting growing interest in bioimaging due to their intrinsic fluorescence (e.g., pyrene‐based COFs), which enables the label‐free tracking of tumors. However, bulk COFs are limited by poor water dispersibility (due to strong *π*–*π* stacking), aggregation‐induced fluorescence quenching (reducing imaging sensitivity), and limited bioavailability (large particle size hinders cellular uptake). These obstacles motivated Seokmin Kang and his lab to design ultrathin covalent organic nanosheets (CONs) from the exfoliation of 3D pyrene‐based COFs. Liquid exfoliation processing (LEP) is a method of disintegrating of bulk COFs using physical (in this case, ultrasonication for 5 h) or chemical forces to disrupt interlayer bonds, causing layered materials (like graphite or COFs) to separate into nanosheets. Here, the TpPy COF was synthesized from Tp (1,3,5‐triformylphloroglucinol)‐a trifunctional aldehyde‐ and Py (2,7‐diaminopyrene)‐ a fluorescent diamine‐ units through a Schiff‐base condensation under solvothermal conditions. The in vitro confocal imaging indicated the confirmed internalization of TpPy CONs inside the cells and red fluorescence from pyrene units allowed label‐free tracking. Additionally, the in vivo studies on MDA‐MB‐231 tumor‐bearing mice indicated strong fluorescence at tumor sites within 1 h after injection, which prolonged for 24 h [419]. Another example of preventing the fluorescence quenching is a dual‐mode theranostic system namely COF‐980, which serves as both a NIR‐II imaging probe and a photosensitizer for PDT in 4T1 tumor‐bearing mice. the COF‐980 was constructed from covalent bonds between L‐BBTD (benzobisthiazole‐based linker, provides NIR‐II emission and generates ROS simultaneously) and TAPP units, with strong absorption/emission at 808 nm (excitation) / 980 nm (emission). Shifting the imaging window from visible/NIR‐I (700–900 nm) to NIR‐II (1000–1700 nm) offers deeper tissue penetration and reduced scattering, which detectable signals up to 8 mm depth in tissue‐mimicking phantoms proved it. In vivo, COF‐980 NPs accumulated maximally in tumors at 36 h post‐injection, enabling clear NIR‐II imaging with high tumor‐to‐background contrast. Furthermore, when combined with laser irradiation, the NPs achieved near‐complete tumor suppression (∼90% inhibition) and extended survival up to 45 days (vs. rapid progression in controls), with no systemic toxicity (no weight loss or organ damage in H&E staining) [420]. Instead of using single‐color probes to track only one biomarker, Peng Gao et al., assembled a tricolor nanoprobe to visualize three distinct signals (typically three different fluorescence colors) in a biological system and eliminated the need for sequential staining or separate probes. This nanoagent contained Cy5‐labeled MUC1 aptamer to detect MUC1 protein on cell membranes and TAMRA‐labeled survivin antisense DNA, which binds to survivin mRNA in the cytoplasm. The COF's extended π‐conjugated structure broadly absorbs light, quenching the fluorescence of attached dyes (Cy5/TAMRA) via Förster resonance energy transfer (FRET) or π‐stacking interactions when DNA is adsorbed. Upon binding to targets (MUC1/survivin), the dyes are released from the carrier, leading to the restoring dye fluorescence (“turn‐on” signal). Besides, The COF's intrinsic blue signal acts as an internal control to track NP localization independently from biomarker signals, providing a third channel for imaging. The results of applying this nanocarrier for both normal (MCF‐10A) and cancer cells (MCF‐7) are depicted in Figure 22a, showing higher COF fluorescence intensity in MCF‐7, suggesting MUC1‐mediated enhanced uptake and no false‐positive signals in normal cells [421]. In a report by Hao Ye and collaborators, the cellular uptake of nano‐sized COFs (nCOFs), cytotoxicity mechanisms (apoptosis, ferroptosis, autophagy, necroptosis), and in vivo biodistribution in healthy and tumor‐bearing mice was tested by two set of imine‐based COFs including 2D COF‐1 and 3D‐COF‐300 [422]. In biomedical applications, to maximize cellular uptake, avoid rapid kidney clearance and splenic filtration, the size of the particles are usually optimized to fit in the 10–200 nm range, and in this experiment, characterization revealed monodisperse particles with dimensions tightly clustered near 35 nm (±5 nm). The cellular uptake of each COF from different cell lines involving normal and cancerous cells demonstrated the rapid entrance of nCOFs into the cells after only 30 min of injection and higher uptake of spherical nanoparticles comparing to their nanorods counterparts (1–2 µm), as they are too large for endocytosis. The biodistribution of nCOFs containing near‐infrared fluorescent dye, 1,1′‐dioctadecyl‐3,3,3′,3′‐tetramethylindotricarbocyanine iodide (DiR) in both healthy and tumorous mice is illustrated in Figure 22b. Following intravenous injection, the nanoparticles spread throughout the body within 1 h, with sustained detection for up to 24 h. In healthy mice, nCOFs were primarily localized in the liver, spleen, and lungs, whereas the concentration of free DiR dye increased mainly in the lungs and liver. The situation of tumor‐bearing mice was observed different, as both PEG‐COF‐1@DiR and PEG‐COF‐300@DiR showed significant and preferential accumulation in osteosarcoma tumors, with peak concentrations observed at 4 h post‐injection and retention for 24 h.

#### 5.5.3 Biosensing

**FIGURE 23:**
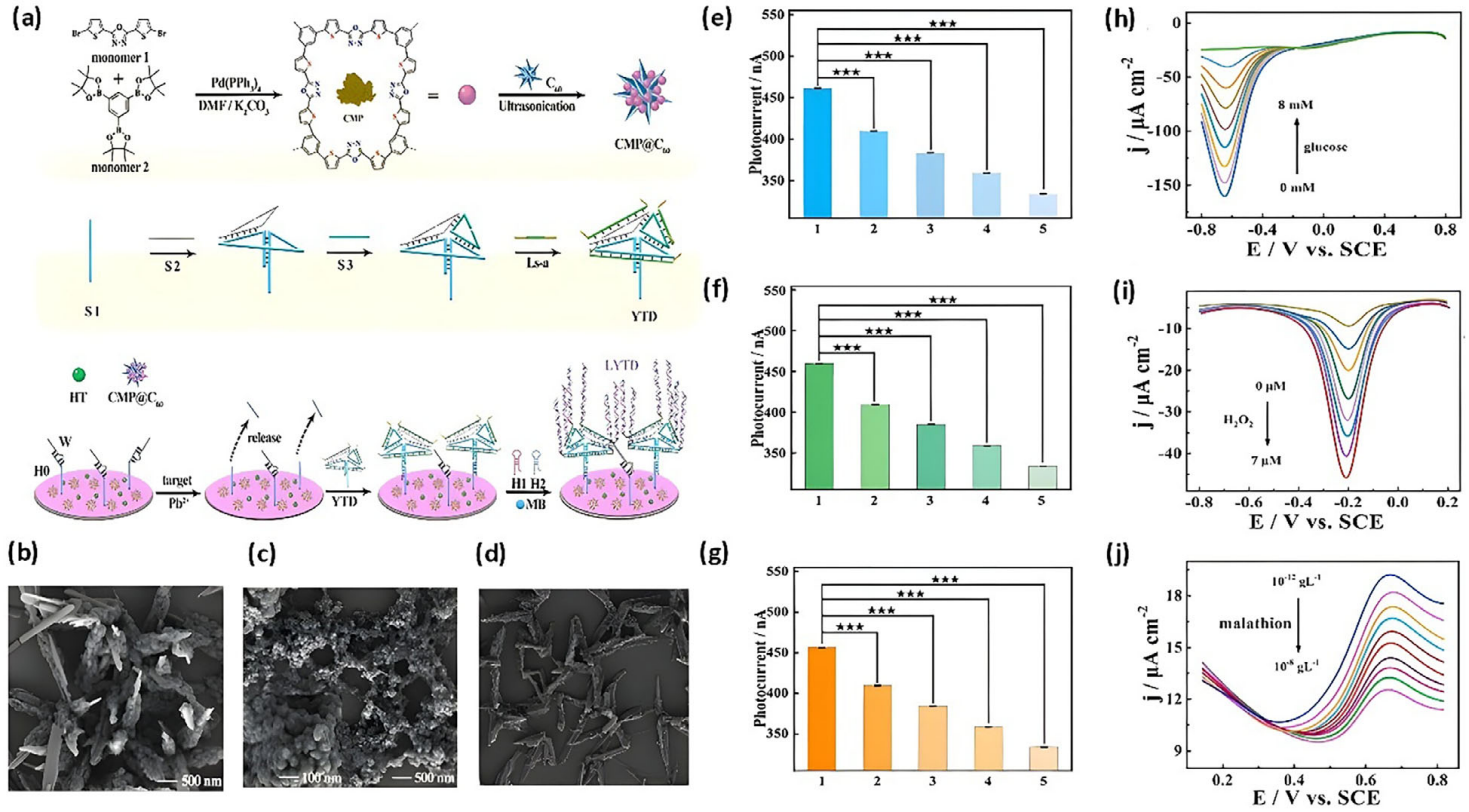
(a) Schematic illustration of the construction of PEC sensor for Pb^2+^ including the synthesis of CMP@C60, preparation of YTD and assembling the PEC biosensor; (b–d) SEM images of C60 (b), CMP (c) and CMP@C60 (d); (e‐g) recovery experiments of proposed biosensor in (e) tap water: 1, blank; 2, 1 fM; 3, 10 fM; 4, 100 fM; 5, 1 pM; (f) lake water: 1, blank; 2, 1 fM; 3, 10 fM; 4, 100 fM; 5, 1 pM; and (g) clinical serum: 1, blank; 2, 1 fM; 3, 10 fM; 4, 100 fM; 5, 1 pM. Reproduced with permission from ref. [423]. Copyright 2023, Elsevier. (h–j) DPV of enzymes@COF/COFTFPB‐DBD/GCE in 0.2 M O2‐saturated PBS (pH 7.0) with continuous addition of glucose (h), H_2_O_2_ (i), and malathion (j). Reproduced with permission from ref. [424]. Copyright 2021, Elsevier.

Unlike conventional electrochemical biosensors, which rely solely on redox reactions at an electrode, photoelectrochemical biosensors (PEC) sensors benefit from lower background noise due to the separation of excitation (light) and detection (current). Researchers under Ying Jiang's direction formulated a state‐of‐the‐art sensor which consists of a glassy carbon electrode (GCE) modified with CMP@C_60_, to detect lead ions (Pb^2+^) in water at fM levels [423]. The CMP@C_60_ is a photoactive combination, responsible for generating electricity when exposed to light, but because of the fast energy loss of CMP (electrons and holes recombine), the fullerene (C_60_) was integrated to trap electrons and prevent energy loss. As it can be seen in Figure 23a, AuNPs are electrodeposited to immobilize a hairpin DNA probe (H_0_). They also fabricated a Y‐shaped DNA scaffold named long‐tailed Y‐triangular DNA (LYTD) structure with long tails, which loads MB, a quencher that competes with CMP@C_60_ for light absorption and reduces the photocurrent in a “signal‐off” mechanism. When Pb^2+^ is present, it cleaves H_0_, exposing a sequence that triggers hybridization chain reaction (HCR) and opens the way for the attachment of LYTD. The “light shadowing effect” of the quencher results to an electrical current drop which correlates with Pb^2^^+^ concentration, enabling sensitive detection. Figure 23e–g, show the photocurrent results of real‐sample applications, including tap water (e), lake water (f) and clinical serum (g), validating the biosensor's accuracy in complex matrices. Among the electrochemical biosensors, enzyme‐based electrochemical biosensors are particularly valuable because enzymes offer high catalytic efficiency and specificity. Huihui Liang and colleagues attempted to engineer an enzyme@COF microcapsule‐based electrochemical biosensors, to overcome the common drawbacks, such as receptor detachment from electrode surfaces, through spatially confined yet conformationally liberated enzymes, for organophosphorus pesticides (OPs), glucose monitoring, and neurotransmitter analysis [424]. The enzymes@COF/COFTFPB‐DBD/GCE biosensor integrates three enzymesglucose oxidase (GOD), horseradish peroxidase (HRP), and acetylcholinesterase (AChE), within a COF microcapsule, which is immobilized on a glassy carbon electrode (GCE). The COF plays the roles of shielding the enzymes from harsh environments, allowing enzymes to move freely, preventing enzyme leakage, and facilitating analyte diffusion through the pores. Glucose was detected under the mechanism of oxidation by GOD to gluconic acid, showing a decrease in current at −0.65 V as depicted in Figure 23h. Meanwhile, in the presence of H_2_O_2_, the Fe^2+^ in the HRP was converted to Fe^3+^, accompanied by a reduction peak of Fe^3+^ at − 0.21 V (Figure 23i). Normally, AChE hydrolyzes acetylthiocholine (ATCl) to thiocholine (TCl) and acetate, with a measurable oxidation current at the electrode. The irreversible binding of malathion as a typical OPs in this work, to AChE prevents TCI production and thus, causing a drop in the electrochemical signal proportional to its concentration (Figure 23j). OPs like methyl parathion, paraoxon, and malathion are highly toxic pesticides that can cause severe poisoning even at trace levels, making their detection crucial for food safety and environmental monitoring.

The broad range of applications discussed above highlights the remarkable versatility of POPs across environmental, catalytic, energy, biomedical, and separation technologies. However, the practical advantages of POPs are best appreciated when compared with other established porous materials, including MOFs, COFs, activated carbons, zeolites, and conventional functional materials. To provide a concise overview of their relative strengths and limitations, a comparative summary is presented in Table 6.

**TABLE 6:** A comparative summary of POPs, MOFs, and COFs, vs. traditional materials, such as activated carbons, zeolites, in various functional applications.

Application area	POPs	MOFs	COFs	Traditional materials (activated carbon/zeolites, etc.)	Key comparative insight	Refs.
CO_2_ Capture & Gas Separation	High chemical stability, tunable pore functionality, strong resistance to humid conditions, moderate‐to‐high selectivity	High surface area and uptake capacity, but moisture‐sensitive in many systems	High crystallinity and tunable channels, but scalability/processability remain challenging	Activated carbons: cheap but low selectivity; Zeolites: strong adsorption but humidity‐sensitive	POPs offer a balance between stability, tunable chemistry, and industrial robustness	POP [96] COF [266, 267, 268, 269, 270, 271, 272] MOF [285]
Catalysis	Robust covalent frameworks, tunable active sites, excellent recyclability	Highly accessible metal centers and high catalytic activity	Ordered active channels and controllable electronic structures	Homogeneous catalysts often show poor recyclability and stability	POPs provide superior structural durability and catalyst recovery	POP [319] COF [303]^,^ [304]
Photocatalysis	Broad functionalization capability, adjustable donor–acceptor structures, improved stability	Efficient charge separation but possible metal leaching	High π‐conjugation and charge transport	Conventional semiconductors often suffer from low tunability	POPs enable rational electronic structure engineering with enhanced long‐term stability	POP [326] MOF [322] COF [311]
Energy Storage (LIBs/Supercapacitors)	Flexible redox‐active design, low structural collapse during cycling	High porosity but limited conductivity and framework stability	Ordered ion‐transport channels but difficult scalable processing	Traditional inorganic electrodes suffer from volume expansion	POPs show better structural adaptability and molecular tunability	POP [375] COF [340], [341]
Drug Delivery	High porosity, programmable host–guest interactions, controllable release	High loading capacity but concerns over metal toxicity	Tunable pore size and stimuli responsiveness	Liposomes/capsules often exhibit premature leakage and poor stability	POPs offer superior framework stability and multifunctional delivery platforms	POP [171] COF [417]
Bioimaging	Intrinsic fluorescence tunability, photostability, multifunctional integration	Strong imaging contrast in some metal‐containing systems	Highly optical tunability and NIR responsiveness	Organic dyes suffer from photobleaching	POPs provide aggregation‐resistant fluorescence and structural robustness	COF&MOF [422]
Biosensing	High surface functionality and molecular recognition tunability	Strong electrochemical activity	Highly ordered conductive pathways	Conventional electrodes suffer from lower selectivity	POPs combine porosity with programmable sensing interfaces	COF [423], [424]
Membrane Separation	Good chemical stability and functional tunability	Precise pore apertures but limited processability	Highly selective nanochannels	Polymeric membranes often face permeability/selectivity	POPs provide improved functional adaptability and long‐term stability	POP [425] COF [138] MOF [285]
Scalability & Industrialization	Some systems compatible with green synthesis and continuous‐flow production	Scale‐up often limited by expensive ligands and metal precursors	Crystallinity control and insolubility complicate industrial fabrication	Activated carbons already industrially mature	POPs remain promising but still require improved processability and cost reduction	POP [426], COF [427]

## 6 Recent Trends and Current Challenges of Organic Polymers

### 6.1 Hybrid Organic Frameworks

**FIGURE 24:**
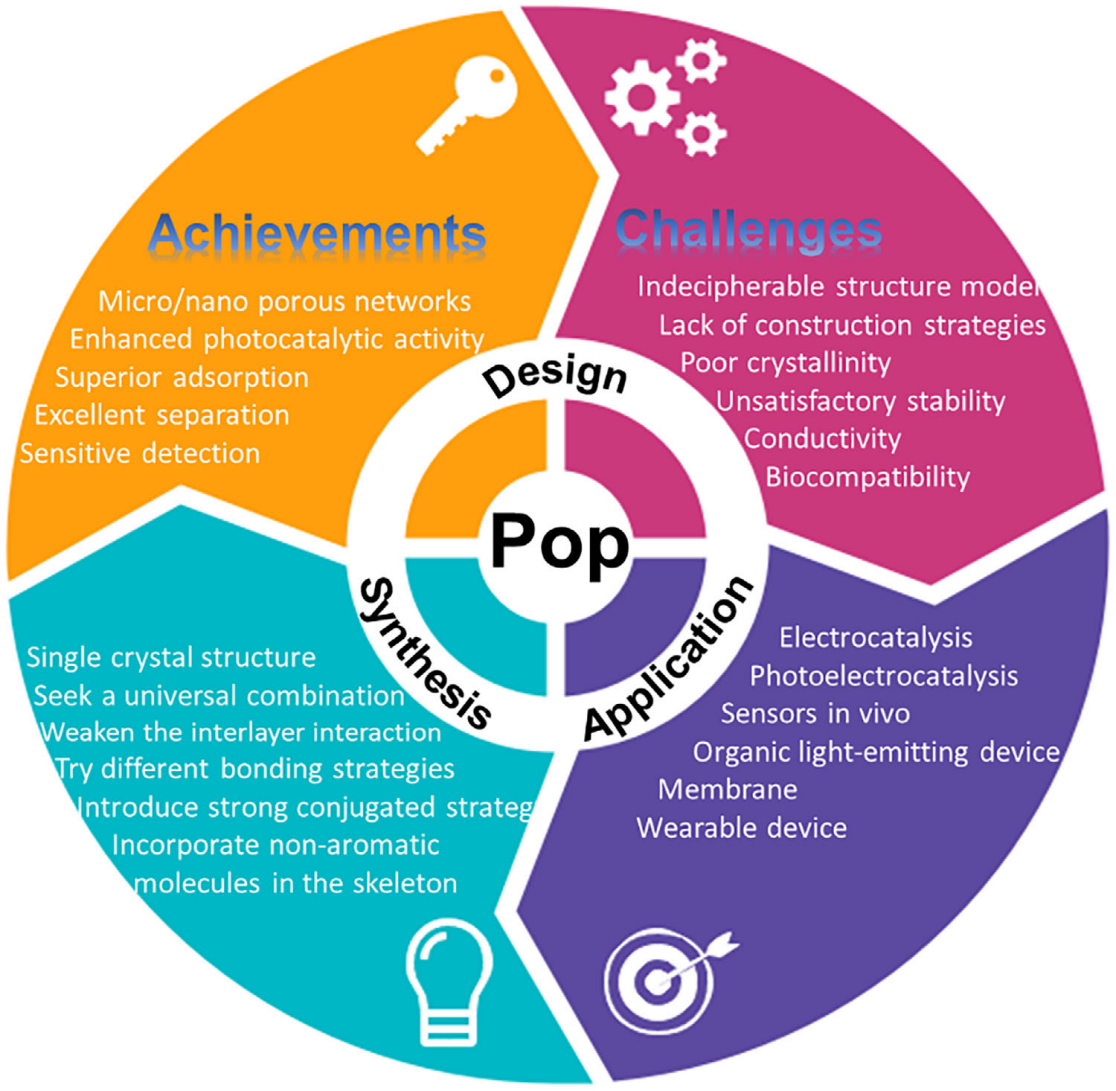
The challenges and potential applications of POP, such as MOF/COF hybrid materials.

Although MOF/COF hybrid materials have advanced the field of porous materials, several challenges remain in their development. First, achieving atomically precise structural models is difficult but essential for guiding the design and understanding of the mechanisms. Single‐crystal structures can be achieved by carefully selecting the building blocks and linkers. Second, the synthesis of MOF/COF hybrids is limited and requires improvement. Developing universal methods that combine their advantages, correct their deficiencies, and enhance their properties is crucial. This may involve strong covalent (C = C and C≡C) linkages, metal coordination, hydrogen bonds, or physical interactions. Third, although MOF/COF hybrids offer promising hierarchical porosity for various applications, they remain underexplored in electrocatalysis, which is a key area for addressing energy and environmental challenges. Moreover, strong interlayer interactions and rigid structures often hinder their biocompatibility. This can be mitigated by adjusting the interlayer spacing, incorporating hydrophilic groups, or using non‐aromatic building blocks (Figure 24) [428].

### 6.2 Structural and Processing Challenges in Advanced POP Architectures

The development of structurally diverse POPs, including porous organic cages (POCs), nanofibrous POPs, and COFs, has opened new possibilities for applications in membranes, catalysis, sensing, and actuation. However, each architecture presents specific hurdles in terms of synthesis, processing, and application. POCs, with their unique solubility and internal cavities, are attractive for solution‐processable membranes, films, porous liquids, and catalysis applications. However, a general synthesis strategy remains elusive. Irreversible linkages yield robust but low‐yield products, whereas dynamic covalent methods offer higher yields but limited stability, which is problematic for industrial use. Additional challenges include difficulties in growing high‐quality single crystals, collapse upon desolvation, and limited application of computational modeling due to their structural flexibility. Real‐time monitoring, and improved synthetic control, are critical for overcoming these challenges [429, 430].

COFs, known for their tunable optoelectronic properties and design flexibility, face limitations owing to their insolubility and microcrystalline nature, which hinder their mechanical performance. Although embedding COFs in polymer matrices, such as PVDF or PMMA, can enhance their structural integrity, these matrices often lack added functionality. For instance, the toughness, as measured by the area under the stress‐strain curves, nearly doubled (from 2.6 MPa in pure PMMA to 5.6 MPa in PMMA‐molecular woven), and the strain at failure of PMMA‐molecular woven composites (3% weight) rose from 0.13 to 0.22 mm/mm. A promising approach involves combining COFs with stimuli‐responsive polymers, such as liquid crystal polymers (LCPs), to create advanced actuators with multifunctional responsiveness [431].

### 6.3 Catalytic Application

The main challenge in the catalytic potential of POPs (especially the metalated POPs, “M‐POPs”) is to create a material that offers structural strength (due to its covalent bonds). It delivers high catalytic activity through its accessible metal sites. Traditional catalysts tend to fall short in this area, often exhibiting low conversion rates. Boro et al. [432] developed a novel Fe‐metalated POP (Fc‐Bz‐POP) by linking benzothiazole to ferrocene to address this issue. They used a simple, cost‐effective, template‐free, one‐pot synthesis that combined multiple components to achieve stability and catalytic functionality. This new material features an extensive network with well‐separated Fe active sites and a unique catalytic pocket where substrates can gather, thereby boosting the reaction efficiency. Fc‐Bz‐POP proved its worth in catalyzing the allylic C–H bond functionalization of cyclohexene in water at room temperature, reaching an impressive 95% conversion. Detailed structural studies using EXAFS and ATR‐IR confirmed that the iron atoms in the material adopted a “sandwich‐like” arrangement, similar to that in ferrocene, which stabilized the catalyst and improved its electronic characteristics. DFT calculations provided insights into the reaction mechanism, revealing key intermediates and demonstrating how the polymer framework fine‐tunes the electronic environment around the Fe sites, resulting in outstanding catalytic performance.

In another approach, by utilizing the Sonogashira alkyne coupling reaction along with coordination of several metals, including Fe^2+^, Co^2+^, and Ni^2+,^ several metal‐coordinated CMPs have been developed for use as contenders in novel electrocatalysts in the oxygen evolution reaction systems, in which the resultant metal‐coordinated CMPs possess superior thermal and electrochemical characteristics in comparison to non‐metal‐coordinated CMPs [433].

In terms of the hydrogen evolution reaction, novel imide‐imine‐based COFs have been developed to overcome the insufficient charge separation challenge in this field. Simultaneous incorporation of electron acceptors and donors into the backbone of the COF facilitates the intramolecular charge transport, which leads to an increase in the charge separation [434].

As a new generation of photocatalysts, pyrene‐benzotiophene‐anthracene‐based CMP has been synthesized via Suzuki coupling polymerization. According to the presence of pyrene and anthracene as electron donors and benzothiophene as electron acceptors, the resultant engineered CMP has a high conductivity as well as significant thermal stability up to around 600°C [435]. These authors also investigates the capability of triphenylamine (TPA) and alkyne groups in Suzuki and Sonogashira–Hagihara coupling reactions between triphenylamine (TPA) and alkyne groups via modulating the acceptor and donor site of the CMP and their results showed that the implementation of alkyne groups significantly suppresses electron–hole recombination, which leads to extension of lifetime of charge carriers and enahnce the overall photocatalytic activity compared to non‐alkyne‐based‐ CMPs [436].

Although amorphous POPs have shown promising results in photocatalysis, they face several challenges, including moderate conversion efficiencies and limited reaction scopes. To address these challenges in visible‐light‐catalyzed organic transformations, several key directions must be pursued as follows [66]:

- Diversifying Amorphous POPs: While CMPs dominate current research, other classes, such as PIMs and HCPs, offer unique properties, such as solubility from PIMs’ rigid pores or milder synthesis conditions from HCPs. A rational design that integrates these features can broaden the photocatalytic applications.
- Develop Greener and Cost‐Effective Synthesis: Current solvothermal methods often involve harsh conditions, hazardous solvents, and long reaction times. Emerging techniques such as ultrasonic, microwave, and light‐assisted syntheses offer faster and more environmentally friendly routes. Additionally, many POP photocatalysts depend on metal co‐catalysts, which increases costs and raises environmental concerns due to potential metal leaching. Therefore, the development of efficient metal‐free organic photocatalysts is crucial.
- Expand Reaction Scope: Present studies focus on sulfide oxidation, oxidative coupling of amines, and tertiary amine dehydrogenation. Broadening the repertoire to include reactions such as the Aza‐Henry reaction and [2+2]. cycloadditions, which are important in pharmaceutical and industrial chemistry, could yield significant advancements, particularly if such reactions can proceed under milder conditions with POPs.
- Enhanced Light Absorption: Many amorphous POPs are limited to specific wavelengths, reducing sunlight utilization efficiency. Strategies to expand the absorption range include extending polymer conjugation and reconfiguring material morphologies, such as converting irregular particles into tubes or thin films, to increase the active surface area.
- Deepen Mechanistic Understanding: Current mechanistic insights rely mainly on experimental capture of reactive intermediates, with limited theoretical modeling. Integrating computational studies could offer a deeper understanding of photocatalytic processes and guide the design of next‐generation amorphous POPs for improved performance.

### 6.4 Environmental Applications

POPs offer tremendous promise for applications such as gas separation, CO_2_ conversion, and water purification, owing to their tunable porosity and functional diversity. However, their industrial deployment remains constrained by key limitations: fragility, poor processability, high production costs, and instability under harsh conditions [58, 437].

To address these challenges, researchers have developed hybrid strategies that combine POP‐inspired functionalities with more robust and scalable polymeric systems. One such approach involves modifying conventional membranes, such as polydimethylsiloxane (PDMS) and polytetrafluoroethylene (PTFE), by introducing CO_2_‐philic functional groups. In this method, the membrane surface is first coated with benzophenone, then polymerized with glycidyl methacrylate (GMA), and subsequently reacted with ethylene diamine (EDA) to anchor amine‐based CO_2_‐attracting sites. These modified membranes achieved a 150‐fold increase in CO_2_ selectivity compared to their unmodified counterparts [425].

In the case of the removal of organic pollutants, metal‐free β‐ketoenamine‐based CMPs were synthesized and developed by mixing the azobenzene (AZO), a phloroglucinol‐derived linker (TFP), and dibenzothiophene dioxide (DBTD). In the resulting CMP, the porosity can be adjusted according to a precise mixing ratio of monomers, and β‐ketoenamine frameworks showed superior thermal properties as well as high efficiency in removing the organic pollutants after three cycles [438].

To further overcome the poor processability of POPs, scientists have developed MMMs by embedding POPs into polymeric matrices.

This integration improves compatibility, mechanical strength, and interfacial integrity, helping to mitigate the formation of defects and expanding POPs’ application potential. Still, significant technical and fundamental barriers remain [18]:

- Incomplete understanding of separation mechanisms: Functionalization with groups like amines and fluorines enhances gas selectivity. One of the approaches to investigate the behavior of polymeric adsorbents is to study isotherm and kinetic models along with adsorption process modeling studies [439]. Molecular dynamics (MD) simulations method allows the study of material structure and adsorption behavior at the molecular scale and enable the analysis of physicochemical parameters [440]. These methods combine the analysis of transport phenomena at the atomic and molecular levels with using physical and chemical theories.
- Structural stability concerns: POPs particularly COFs can undergo pore collapse or degradation due to weak interlayer interactions.
- Scale‐up limitations: Membranes optimized for laboratory‐scale testing often underperform in industrial environments, where long‐term stability, pressure tolerance, and cost‐efficiency are critical.
- Fabrication difficulties: The poor solubility and compatibility of many POPs with processing media necessitate novel synthesis and shaping strategies.
- High production costs and sustainability issues: The use of expensive or toxic precursors and solvent‐intensive protocols limits commercialization. Green alternatives such as mechanochemistry, solvent‐free synthesis, and hydrothermal condensation offer promise, but challenges in purification and scale‐up persist.

Addressing these issues requires a multifaceted strategy, and a more detailed review of this topic is discussed in more detail in Sections 6.7 and 6.8.

### 6.5 Biomedical Applications

In recent years, COF‐based nanoplatforms have emerged as promising tools for cancer treatment. Owing to their customizable architectures, COFs can be engineered to respond selectively to internal tumor cues (e.g., acidic pH, high glutathione, elevated H_2_O_2_ and ROS levels, and hypoxia) or external triggers, such as light and ultrasound. The high porosity and large surface area of COF nanoparticles (NPs) make them effective drug carriers, offering a superior loading capacity compared to traditional porous materials [441]. However, COF‐based systems face several critical challenges. Controlling the particle size during COF synthesis remains difficult, often yielding particles (> 200 nm) that are unsuitable for biomedical use. Techniques such as template synthesis, ultrasonication, ball milling, and chemical exfoliation can reduce size, but ensuring consistent and reproducible COF NPs with optimal sizes (20–200 nm) remains challenging [441, 442]. Even after size reduction, highly stable COFs may resist intracellular degradation, slowing drug release, whereas less stable COFs risk premature breakdown in circulation. Stimuli‐responsive COFs, which disassemble in response to the tumor microenvironment, offer a solution by combining controlled particle size reduction and targeted drug release [443]. Scaling up COF production for commercial use requires reliable methods for producing uniform and stable nanoscale particles [441]. Additionally, COFs’ inherent hydrophobicity and low dispersibility of COFs limit their biomedical applications; post‐synthetic functionalization with hydrophilic groups can improve their stability and dispersion in biological environments [444]. The drug‐loading capacity depends on both the interactions between drug molecules and COF linkers (e.g., hydrogen bonding) and the COF pore structure, which depends on the monomer geometry. Optimizing these aspects could enhance the loading efficiency [445]. Although PSMS are beneficial, they can weaken the interlayer π‐π stacking and facilitate COF exfoliation. The use of diverse motifs (e.g., T_d_) instead of conventional tetragonal or hexagonal geometries can create COFs with larger pores, increasing the drug loading potential. Current COF‐based therapies primarily rely on passive targeting, with limited research on active targeting strategies using functional ligands (e.g., peptides and antibodies). Although COFs have demonstrated good biocompatibility in short‐term studies, their long‐term safety, biodistribution, metabolism, and potential toxicity remain underexplored. Stimuli‐responsive COFs may offer advantages in both targeted drug release and clearance from the body, thereby reducing the risk of long‐term accumulation in organs [441].

Translating COFs and MOFs into medical applications faces challenges, including rigorous international regulations, particularly for imaging agents administered to healthy individuals, which require exceptional safety profiles. Collaboration among regulatory bodies, researchers, and industries is vital for approval. Many promising technologies show early promise but fail in clinical trials, as observed with thermosensitive liposomes. Current nanotechnologies, such as Doxil and Abraxane, although innovative, are based on established principles and face long timelines for approval. Overhyped claims of nanotechnology's benefits, such as the exaggerated Enhanced Permeability and Retention (EPR) effect, can mislead and foster unrealistic expectations. Targeting efficiency in drug delivery remains a challenge, despite the development of improved encapsulation methods. Toxicity concerns regarding MOFs can be mitigated by using biocompatible metals (e.g., Mg, Zn, Ca, and Ti) and biodegradable linkers. However, economic factors and conservative industry priorities may delay the commercial viability of COF‐ and MOF‐based therapies, potentially for decades or longer [58].

### 6.6 Energy Storage

POPs used as electrode materials in LIBs continue to encounter challenges, including low electroactivity and poor electrical conductivity. To address these issues, the incorporation of redox‐active functional groups or the introduction of dual/multi‐active centers can enhance electroactivity, while the integration of conductive carbon materials improves electrical conductivity [446]. SIBs are a promising and cost‐effective energy storage option owing to the abundance of sodium. Traditional anode materials experience significant volume expansion during sodiation, leading to poor electronic contact and capacity fading [447]. In contrast, organic electrode materials undergo minimal structural changes during Na^+^ insertion/extraction owing to bond rearrangement during redox processes. However, these materials face challenges such as low conductivity, limited electroactivity, and dissolution issues. To address these issues, Guo et al. [448] developed a conjugated hierarchical POP with a high rate capability and long cycle life, while Chuo et al. [377] introduced a rigid amorphous POP (NDI‐TFP) as a cathode material, which was exfoliated to improve redox site accessibility and electrochemical performance, marking a pioneering use of exfoliated redox‐active amorphous POPs for SIBs. To improve the overall performance of Lithium‐sulfur batteries (Li‐S), a metalloporphyrin‐based POP has been synthesized as a 2D polymeric ionic liquid framework with the incorporation of lithium polysulfide, and catalytic functions to act as a separator coating, which facilitates the polysulfide diffusion in Li‐S batteries [449].

By leveraging the Schiff‐base reaction and utilizing thiophene as a linker, a pyrene‐thiazole‐based CMP has been developed as an energy‐storage supercapacitor, where the energy‐storage characteristics have been enhanced by combining the conductivity of thiophene with the electronic properties of thiazole [450]. In another similar study, which was conducted by Basit et al. [451], utilizing the Schiff‐base polycondensation reaction and engineering synergy between the anthracene and pyrene‐thiazole‐based CMP, revealed the key role of thiazole in adjusting the redox capability of the developed CMP, which leads to improving the capability of organic polymers for future potential application in energy storage applications.

A novel COP has been developed based on the pyrene monomer (Py‐Ph‐CHO) and 4,4′‐disulfanediyldianiline (DSDA) by Ejaz et al. Different single and multi‐walled carbon nanotubes have been incorporated into the COP's structure, and the results showed that due to lower diameter, better dispersion, and higher conductivity, single‐walled carbon nanotubes have better performance in supercapacitor energy storage in comparison to multi‐walled carbon nanotubes [452].

### 6.7 Scale‐up Production Challenge

One of the main obstacles to commercialization is the inherent conflict between high‐throughput synthesis and industrial‐scale mass production. Currently, most high‐throughput POPs, especially highly crystalline COFs, are produced by solvothermal reactions in batch mode [453]. This method, although excellent for achieving the best material structure, is very challenging on an industrial scale. It is characterized by very long reaction times, significant energy consumption, and a heavy and often unavoidable dependence on large volumes of toxic organic solvents [454].

In this direction, considering green and low‐cost synthesis is a priority for economic necessity. Recent research work has shown that high‐throughput POPs can be synthesized on a sub‐kilogram scale using inexpensive and readily available monomers under green conditions [455, 456]. Overcoming these obstacles requires a deliberate shift from specialized multi‐step precursors in favor of commodity‐scale chemicals. This shift is effectively supported by the adoption of methods such as water‐based synthesis [457]. By eliminating the need for expensive, high‐boiling solvents, researchers can simultaneously reduce operational overhead, simplify downstream purification, and minimize the ecological footprint, each of which is a determining factor for scale‐up and industrial feasibility. In parallel with these chemical optimizations, a shift from traditional batch production to continuous production processes is essential to ensure the throughput and stability required for large‐scale production. A shift from conventional batch processes to continuous production frameworks and processes is essential for industrial levels. Batch systems are inherently prone to internal thermal gradients and mixing inhomogeneities, both of which compromise the uniform environment necessary for reproducible polymer growth. Even subtle fluctuations in these reaction kinetics can propagate through the synthesis, leading to batch‐to‐batch variability that often disrupts quality control in large‐scale operations [458]. Conversely, continuous flow reactor processes offer millisecond‐level precise control over reaction parameters, enabling rapid and highly reproducible synthesis of POPs [459]. Furthermore, in the case of COF industrialization, it can be predicted that the future of this class of materials depends on these automated and continuous processes to ensure market acceptance [427].

The commercialization opportunities for POPs can be divided into two parts. The first is for specialized catalysis and advanced separations, and the second is for large‐scale applications such as water purification and gas storage:

- Commercialization for specialized catalysis and advanced separations POPs provide a robust and heterogeneous platform for catalysis that often surpasses the selectivity and recyclability of traditional homogeneous systems [460]. For example, the exceptional performance of rhodium‐containing POPs in industrial hydroformylation reactions is a clear indication of their potential [461]. Identically, POPs are well suited for the selective removal of contaminants in high‐purity streams, such as the rapid and high‐capacity removal of anionic dyes from water demonstrated by cationic POPs [426].
- Commercialization for large‐scale applications such as water purification and gas storage can create a competitive market. The development of porous sulfur polymers for the effective removal of emerging pollutants from water shows the potential to address major environmental challenges [59]. To compete with established adsorbents such as activated carbon, these materials will ultimately need to be produced at commodity‐scale cost levels, in line with the cost levels of bulk adsorbents used in high‐volume industrial applications. Achieving such goals will require investment in the widespread and sustainable implementation of the green and scalable synthesis routes described above, as these approaches are the only viable route to combining high performance with real cost‐effectiveness on an industrial scale [427, 456].

The path of POPs to industrial implementation faces enormous challenges, each with its own set of obstacles. Cost, complexity of synthesis, solvent use, processability, and reproducibility are some of the issues that require further investigation. Each of these obstacles is assessed separately in the following sections.

#### 6.7.1 Cost

The economic feasibility of POPs is constrained by their high production costs. Specifically, the cost of raw materials presents a major obstacle, as the synthesis of high‐performance POPs often necessitates complex organic monomers. This reliance on multi‐step synthesis renders the material costs impractical for widespread industrial application [427]. As a recent techno‐economic analysis on related porous materials highlights, while the linker itself is a primary cost driver, the subsequent processing steps, including extensive purification and solvent recovery, add significantly to the final price [462].

#### 6.7.2 Synthesis Complexity

The synthesis of many highly ordered POPs, such as COFs, is often a delicate, high‐wire act of chemical precision. It is characterized by multi‐step, sensitive reactions requiring stringent conditions, inert atmospheres, high temperatures, and precise modulator control [463]. The path to industrial acceptance requires a radical simplification of the synthetic protocol. This means prioritizing one‐pot, ambient‐condition syntheses that can be executed without specialized inert‐atmosphere handling [464]. Furthermore, the adoption of green and large‐scale synthesis methods, such as those that accelerate the reaction kinetics and reduce the need for complex post‐synthetic workup, is essential to de‐risk the manufacturing process and ensure it is viable for large‐scale production [465].

#### 6.7.3 Solvent

The heavy reliance on large volumes of organic solvents in solvothermal synthesis is arguably the most obvious industrial drawback in current POP synthesis. Solvents such as DMF or mesitylene are often toxic, difficult to recycle, and require significant energy for removal, disposal, and recovery [466]. One of the most effective solutions is to adopt green synthesis strategies. This strategy can involve the complete elimination of solvents by switching the route from hazardous chemicals to safe alternatives such as water or ethanol. By eliminating the liquid phase, mechanochemical routes offer a rapid and inherently scalable approach that directly reduces the economic and environmental costs associated with traditional synthesis methods [457].

#### 6.7.4 Processability

The structural properties of POPs, such as their high crystallinity and inherent insolubility, are parameters that pose a major challenge for industrialization. These materials are usually synthesized in the laboratory scale as fine powders, which makes their mechanical shaping for commercial applications such as coatings or membranes challenging [467]. Mechanical processing and processability are a challenge on the path to commercialization. To overcome this challenge, one of the most practical approaches is the mixed matrix material (MMM) manufacturing strategy. By implementing high‐performance POPs in a processable polymer matrix, it is possible to produce them on a large scale [427]. Another approach is to convert them into film forms, such as thin films or Electrospun fibers, which transforms a simple, rigid material into a flexible composite for commercialization [468].

#### 6.7.5 Reproducibility

Implementing a laboratory product on an industrial scale presents many challenges. For POPs, the biggest challenge is ensuring that their structure is uniform and reliable on a larger scale. Indeed, their key properties, such as surface area, crystallinity, and pore size, can change dramatically with even minor changes in their fabrication. In fact, the physicochemical properties of these materials, especially their surface area, crystallinity, and pore size distribution, are very sensitive to the synthesis conditions. This major challenge translates into structural incompatibilities that make these materials unacceptable for commercialization [467]. To overcome this major challenge, the path of laboratory synthesis must move toward the integration of precise process automation. Continuous‐flow synthesis is a very powerful tool to overcome this challenge. Using this method, we can continuously monitor the process flow and ensure that the quality of the materials is consistent from batch to batch [465].

The evolution of the mentioned technology development requires fundamental changes in the production of the POPs pathway. The POP production strategy should be defined in such a way that the production of these materials is simple and environmentally friendly. A production pathway that ensures the structure of POP materials remains intact at all stages could be the key to overcoming the aforementioned major obstacles.

### 6.8 AI‐Assisted Discovery and Optimization of Organic Frameworks

In recent years, the discovery and development of MOFs and COFs have surged because of their structural versatility and wide range of potential applications, from gas storage to catalysis and sensing. However, progress in this field is often hindered by persistent challenges, such as poor crystallinity, scalability issues, and the complexity of synthesizing high‐quality frameworks. Traditional trial‐and‐error experimentation requires significant time and expertise, while difficulties in growing large, uniform crystals and understanding the structure–property relationships further hinder advancement. Additionally, frameworks such as MOFs often exist as microcrystalline powders, making it difficult to incorporate them into functional devices without compromising their mechanical or structural integrity [469].

To address these barriers, researchers have begun turning to artificial intelligence (AI), particularly large language models (LLMs) like CahtGPT, to accelerate the design and discovery process [470]. By embedding GPT‐4 into iterative laboratory workflows, researchers have successfully optimized reaction conditions, guided material synthesis, and discovered new MOF structures with improved crystallinity. This human‐AI collaboration has allowed single scientists to work with the efficiency of entire teams. In addition to AI tools, new directions such as multivariate MOFs, hybrid composites, and MOF‐based devices are being explored to boost material functionality and practical deployment [471]. For example, recent research on MOF synthesis using experimental data extracted from scientific literature and published papers on CO_2_ capture applications using LLM has created a specific and practical path in the use of data. A systematic approach to extract synthesis data and analyze the effects of MOF material structure on CO_2_ capture performance has been able to introduce AI as a powerful assistant [472]. Recent studies in the field of computational material structure via atomic and molecular scale for the development of materials such as MOFs for gas adsorption applications have drawn a clear connection between the prediction of material synthesis and atomic structures by ML algorithms. Research studies such as the screening of materials using quantum computational data and their role in the feed of ML algorithms introduce future prospects for the development of novel synthesis routes [473].

Looking to the future, the integration of smart systems, real‐time synthesis monitoring, and predictive modeling will not only accelerate access to advanced materials research, but will also revolutionize the discovery, scaling, and application of organic frameworks in upcoming technologies [474].

To synthesize and manufacture a new polymer, several factors will play a role. At the first, using key concepts from the results of experiments that originate from the achievements of expert scientists and engineers, using repeated experiments and molecular simulation methods that require specialized skills, budget, and time; a huge exploration is carried out in a vast space of materials. The new polymer must meet various criteria in terms of properties, performance, and safety, as well as environmental and cost considerations, which are very important, and if all the desired considerations are met, the scalability of production and all possible barriers must be assessed for marketing.

Today, with the help of AI and using experimental and computational vast data, this long path can be shortened. The first step is to encode the structure of polymers, which has been strengthened to some extent by the Materials Genome Initiative [475] and acts to complement, strengthen, and increase the impact of experimental or computational materials research. Various indicators suggest that this view is gaining traction in the industry [476], driven by the expectation that such AI‐based knowledge systems can significantly reduce the number and time of iteration cycles before new materials are deployed. Although a significant number of polymeric materials have been discovered, developed, and commercially deployed over the past century, the process has taken many years to commercialize.

**FIGURE 25:**
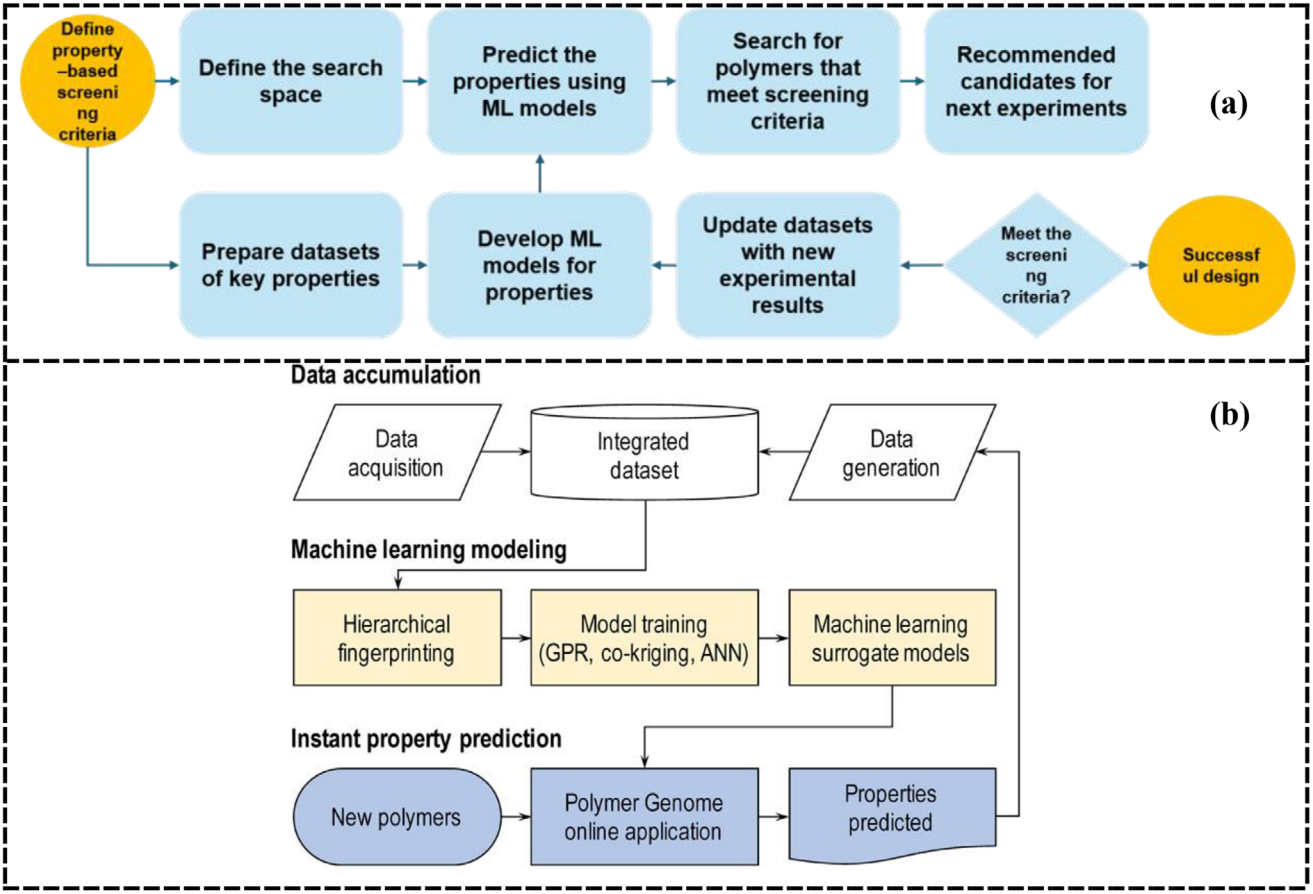
Schema view of (a) novel polymers design flowchart using AI, which is based on the application for polymer design, has common key steps. Adapted with permission from ref. [476]. Copyright 2024, Springer Nature Limited, (b) Schema flowchart of the architecture of Polymer Genome. Reproduced with permission from ref. [478]. Copyright 2020, AIP Publishing.

A robust strategy for application‐inspired, application‐based, and synthetically accessible polymer design involves several steps (see Figure 25). This flowchart begins with defining a set of screening criteria based on the desired property values. A broad search space is then defined, potentially including a big dataset library of synthetically and experimentally produced polymers. In parallel, ML prediction models for these key properties are developed using sufficiently large and diverse datasets of experimental values ​​or computational data [477]. In effect, the models predict key properties of polymers in the search space based on the desired features, and structures that satisfy the desired features are selected as potential candidate structures. The design loop is “closed” by testing the candidate structures through physical experiments. If the candidate structures meet the required property or performance criteria, the candidate is declared a success; otherwise, the design loop cycle is repeated.

Given the time‐consuming process of traditional and experimental methods to determine the properties of new polymers, in parallel, ML models can be used to quickly and accurately predict the properties of desired polymers for the desired application. Computational data, such as the results of DFT or MD simulations, require long computational time and supercomputers, and this is a major challenge in this direction. The development of ML models using experimental or computational datasets makes it possible to reproduce the polymer structure [476]. In this way, the properties of polymers can be categorized and screened, and after screening, samples that require experimental tests and validation can be selected. This valuable data will allow the avoidance of difficult and long experimental paths based on truth and error and will be used to reproduce the polymer structure in the design cycle. This cycle will be repeated to get closer and closer to the target polymer, and based on the prediction results, decisions can be made to continue this path. In the Polymer Genome Platform and Algorithm, it can be used as a practical tool for the design of new polymers. In fact, the polymer genome is the ability to collect and generate polymer data, which is introduced in a coded form, such as a fingerprint, which can be used to represent polymers numerically in a format readable by learning algorithms. Learning algorithms have good potential for using and developing polymer property predictions to solve some practical problems related to property prediction and design [478].

The maturation of new AI algorithms based on materials will enhance researchers' capabilities and scope, accelerating discoveries. Accordingly, with the help of learning algorithms in the field of polymeric materials, it can offer a future in which all the experiences of researchers that have been collected can be used for rapid learning and progress in the field of discovery and customization of various applications of POPs will be developed.

**Nomenclature**
AI: Artificial Intelligence
Ace: Acenaphthene quinone
ASC: Asymmetric Supercapacitor
AAC: Azide‐alkyne cycloaddition
ACAC: Acetylacetonate
Apt: Aptamers
AMP: Ampicillin
AZO: Azobenzene
ATC: Acetylthiocholine
AChE: Acetylcholinesterase
AIBN: Azobisisobutyronitrile
AuNPs: Gold nanoparticles
BET: Brunauer–Emmett–Teller
Bp‐COF: Bipyridine‐based covalent organic framework
BB: Bandrowski's base
BD: Benzidine
BDF: Bonding defect functionalization
BNCT: Boron neutron capture therapy
CEA: Carcinoembryonic antigen
Ce6: Chlorin e6
CPOP: Conjugated porous organic polymer
CPP: Conjugated porous polymer
CMP: Conjugated microporous polymer
CMT: Conjugated microporous thermoset
COF: Covalent organic framework
COP: Covalent organic polymer
CTF: Covalent‐triazine framework
CTCs: Circulating tumor cells
CS: Chitosan
CNTs: Carbon Nanotubes
DAC: Direct air capture
DSC: Differential Scanning Calorimetry
DFT: Density functional theory
DOPA: Dopamine
EC: Ethylene carbonate
EDA: Ethylenediamine
EGDMA: Ethylene glycol dimethacrylate
EMI: Electromagnetic interference
EPR: Enhanced Permeability and Retention
FDA: Formaldehyde dimethyl acetal
FO: Fluorenone
GPU: Gas permeation unit
GPC: Gel permeation chromatography
GO: Graphene Oxide
GOx: Glucose oxidase
GMA: Glycidyl methacrylate
CONs: Covalent organic nanosheets
GCE: Glassy carbon electrode
GI: Gastrointestinal
HCP: Hyper‐cross‐linked polymer
HCR: Hybridization chain reaction
HCPIP: Hyper‐crosslinked porous ionic polymer
HER: Hydrogen evolution reaction
HIP: Hyper‐crosslinked ionic polymer
HPLC: High‐performance liquid chromatography
LIBs: Lithium‐Ion Batteries
LFP: LiFePO_4_ (Lithium Iron Phosphate)
LLM: Large Language Model
MB: Methylene blue
MD: Mo;ecular Dynamic
MCTF: Ionic covalent triazine framework (M‐type)
MOF: Metal–organic framework
MAAM: Methacrylamide
ML: Machine learning
MTX: Methotrexate
NMR: Nuclear Magnetic Resonance
NCOF: Nanoscale covalent organic framework
NIPS: Non‐solvent‐induced phase separation
nCOFS: Nano‐sized COFs
OVS: Octavinyl Silsesquioxane
OPs: Organophosphorus pesticides
PAF: Porous aromatic framework
PBPOP: Porphyrin‐based azo‐porous organic polymer
PDMS: Polydimethylsiloxane
PTFE: Polytetrafluoroethylene
PCNs: Porous carbon nanomaterials
PEG: Polyethylene glycol
PENGs: Piezoelectric Nanogenerators
PEAH: Pentaethylenehexamine
PIM: Polymer of intrinsic microporosity
POM: Polyoxometalate
POP: Porous organic polymer
PVDF: Polyvinylidene Fluoride
PPD: P‐phenylenediamine
PDA: Polydopamine
Pd NPs: Pd nanoparticles
PEC: Photoelectrochemical biosensors
PXRD: Powder X‐ray diffraction
PSMs: Post‐synthetic modifications
QSSEs: Quasi‐Solid‐State Electrolytes
QSPR: Quantitative structure‐property relationship
RA: Rheumatoid arthritis
RED: Reverse Electrodialysis
rGO: Reduced graphene oxide
SAC: Single‐atom catalyst
SCTF: Squaramide‐functionalized covalent triazine framework
SCs: Supercapacitors
SCC: Solar‐to‐chemical efficiency
SDS: Sodium dodecyl sulfate
SSEs: Solid‐State Electrolytes
SHM: Structural Health Monitoring
SE: Shielding effectiveness
SNAr: Aromatic nucleophilic substitution
SIB: Sodium‐ion batteries
TENGs: Triboelectric Nanogenerators
TBAI: Tetrabutylammonium iodide
TETA: Triethylenetetramine
TFTDA: Tetrafluoro‐terphenyl dicarbaldehyde
TFA: Trifluoroacetic acid
TPA: Triphenylamine
TPP: Tetraphenyl porphyrin
TGA: Thermal Gravimetric Analysis
TG: Thermogravimetric
TRO: Truxenone
VOCs: Volatile organic compounds
ZnO: Zinc Oxide

## Conflicts of Interest

The authors declare no conflicts of interest.

## Data Availability

The data that support the findings of this study are available from the corresponding author upon reasonable request.
